# Multiplexed Echo Planar Imaging for Sub-Second Whole Brain FMRI and Fast Diffusion Imaging

**DOI:** 10.1371/journal.pone.0015710

**Published:** 2010-12-20

**Authors:** David A. Feinberg, Steen Moeller, Stephen M. Smith, Edward Auerbach, Sudhir Ramanna, Matt F. Glasser, Karla L. Miller, Kamil Ugurbil, Essa Yacoub

**Affiliations:** 1 Advanced MRI Technologies, Sebastopol, California, United States of America; 2 Helen Wills Institute for Neuroscience, University of California, Berkeley, California, United States of America; 3 Department of Radiology, University of California San Francisco, San Francisco, California, United States of America; 4 Department of Radiology, Center for Magnetic Resonance Research, University of Minnesota Medical School, Minneapolis, Minnesota, United States of America; 5 Oxford Centre for Functional MRI of the Brain, University of Oxford, John Radcliffe Hospital, Oxford, United Kingdom; 6 Anatomy and Neurobiology, Washington University School of Medicine, Washington University, St. Louis, Missouri, United States of America; Cuban Neuroscience Center, Cuba

## Abstract

Echo planar imaging (EPI) is an MRI technique of particular value to neuroscience, with its use for virtually all functional MRI (fMRI) and diffusion imaging of fiber connections in the human brain. EPI generates a single 2D image in a fraction of a second; however, it requires 2–3 seconds to acquire multi-slice whole brain coverage for fMRI and even longer for diffusion imaging. Here we report on a large reduction in EPI whole brain scan time at 3 and 7 Tesla, without significantly sacrificing spatial resolution, and while gaining functional sensitivity. The multiplexed-EPI (M-EPI) pulse sequence combines two forms of multiplexing: temporal multiplexing (*m*) utilizing simultaneous echo refocused (SIR) EPI and spatial multiplexing (*n*) with multibanded RF pulses (MB) to achieve *m*×*n* images in an EPI echo train instead of the normal single image. This resulted in an unprecedented reduction in EPI scan time for whole brain fMRI performed at 3 Tesla, permitting TRs of 400 ms and 800 ms compared to a more conventional 2.5 sec TR, and 2–4 times reductions in scan time for HARDI imaging of neuronal fibertracks. The simultaneous SE refocusing of SIR imaging at 7 Tesla advantageously reduced SAR by using fewer RF refocusing pulses and by shifting fat signal out of the image plane so that fat suppression pulses were not required. In preliminary studies of resting state functional networks identified through independent component analysis, the 6-fold higher sampling rate increased the peak functional sensitivity by 60%. The novel M-EPI pulse sequence resulted in a significantly increased temporal resolution for whole brain fMRI, and as such, this new methodology can be used for studying non-stationarity in networks and generally for expanding and enriching the functional information.

## Introduction

In the last two decades, magnetic resonance imaging (MRI) techniques such as functional magnetic resonance imaging (fMRI) [1], [2] and diffusion weighted imaging for neuronal fiber tractography [3], [4], [5] have revolutionized our ability to investigate the human brain. These techniques mostly rely on echo planar imaging (EPI) [6] for spatial encoding of the magnetic resonance image because of its fast scan time, enabling rapid volumetric coverage over the brain reducing temporal instabilities associated with multi-excitation techniques (e.g. [7], [8]). With contemporary scanner hardware, a single EPI image of a 2D slice can be obtained in tens of milliseconds and is repeated at adjacent positions, requiring 2–3 seconds for whole brain imaging.

Since its initial application, EPI scan time has not substantially decreased. Nearly all the successful efforts to shorten EPI acquisition times have targeted reducing the number of refocused echoes needed for spatial encoding to form an image (by means of partial Fourier [9], parallel imaging [10], [11], [12], or sparse data sampling approaches [13]). Although these approaches decrease scan time for spatial encoding in EPI, with many consequent benefits, they do not necessarily reduce image acquisitions time significantly. This is because a physiological contrast preparation period (i.e. for neuronal activity or water diffusion) must precede the spatial encoding period for each slice and this contrast preparation period can equal or exceed the time employed for collecting the EPI echo train. 3D echo volume (EVI) [14] avoids the repetition of the contrast encoding time by following a single contrast preparation period with subsequent 3D volume coverage in a single echo train. However, this approach has limitations in spatial resolution and image quality due to longer echo trains needed to fully encode the volumetric spatial information in the relatively short acquisition period dictated by T_2_*; the consequence is distortions and blurring on two of the 3D image axes, as well as a loss in signal-to-noise ratio (SNR). Multi-shot (segmented with multiple excitation) 3D EPI approaches that have produced high quality images [15], [16] overcome this limitation albeit at the expense of longer acquisition times than EVI or single-shot 3D GRASE [17]. Echo shifting approaches, PRESTO [18], [19], increase volume coverage efficiency in fMRI by taking advantage of TE delays to apply additional RF pulses, but are SNR limited and run into restrictions at higher magnetic fields when T_2_ and T_2_* become inherently short. Another approach to reducing the scan time per volume in fMRI uses UNFOLD [20]to reconstruct images from undersampled, 3D k-space [21]. Ultimately, the ability to rapidly image the entire human brain with high degrees of precision in space and time is still a major limitation for neuroscience applications. Overcoming such limitations is one of the goals of the recently launched Human Connectome Project (http://www.humanconnectome.org/consortia/) by the National Institutes of Health (NIH). In this paper, we present an approach that accelerates the acquisition of multiple slices in the human brain more significantly than has been previously shown, while not significantly sacrificing spatial resolution or SNR. The method and resulting images are presented together with preliminary data on the application of this approach to resting state fMRI (R-fMRI) and diffusion imaging based tractography.

The pulse sequence we introduce is based on a combination of two techniques for multiplexing signal acquisition, generating several EPI images following the contrast preparation time of a single EPI image. To increase imaging speed, the EPI pulse sequence incorporates temporal (*m*) and spatial multiplexing (*n*) with an increased number of image slices (*mxn*) acquired in a single EPI echo train, thus ***M*** *ultiplexed*-EPI (M-EPI). Time multiplexing is performed by interleaving signals from *m* slices within an EPI echo train, utilizing the simultaneous echo refocused (SER), also known as simultaneous image refocused (SIR) EPI sequence scheme [22]. Spatial multiplexing of signal is performed with multiple receiver coils, each with a distinct sensitivity profile that [23], [24] permit the separation of *n* distinct slices excited simultaneously and acquired in a single EPI train, as we have recently demonstrated for fMRI at 7 Tesla [24]. Incorporating both strategies in a single pulse sequence encodes a relatively large number of images, equal to the product of the two acceleration factors, instead of a single EPI image.

**Figure 1:**
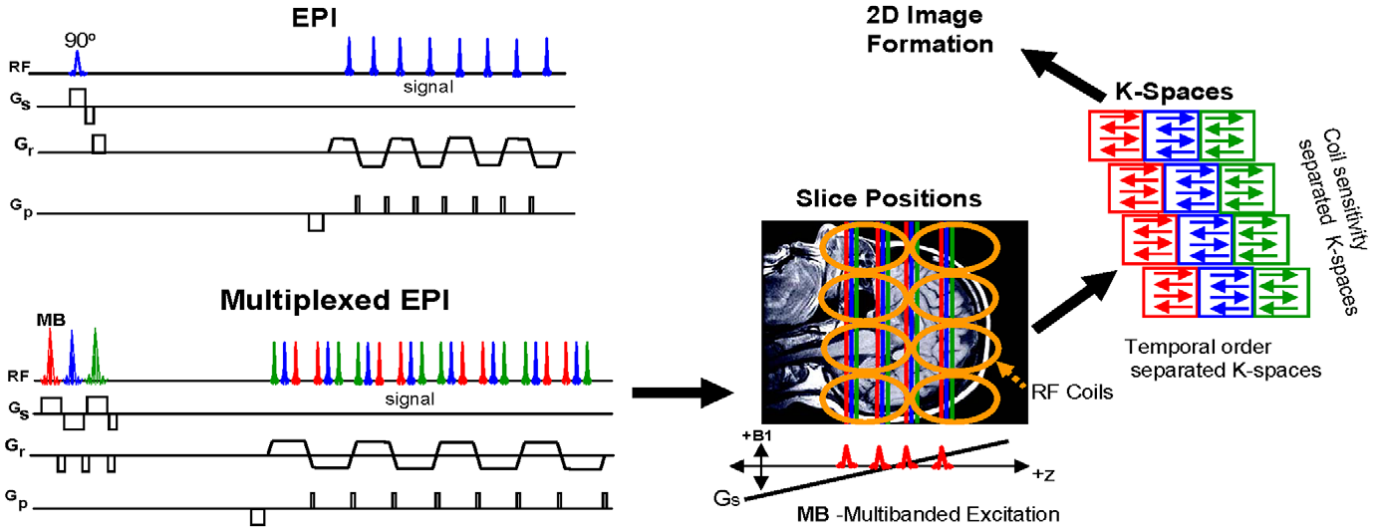
Description of the M-EPI pulse sequence compared with conventional EPI. **Top**) EPI pulse sequence generates a single image during each readout which is repeated by the number of slices to scan the whole brain. **Bottom**) Multiplexed-EPI (M-EPI) pulse sequence generates several images during a single echo train readout and thus requires fewer repeats to scan the whole brain. The multibanded (MB) RF excitation pulses are the sum of *n* frequency offset sinc-modulated pulses which excite slices at widely spaced positions to improve the separation of signal from the different receiver coils. (Slice Positions) show closely spaced SIR images (red, blue, green) and the excitation positions of the *n* sinc pulses of the first MB pulse. The MB pulse is repeated *m* times for SIR excitations and corresponding signals (red, blue, green) are separated (K-Spaces) into individual k-spaces according to their temporal order in the signal readout period. The MB signals (same color) are further separated into k-spaces using the differential coil sensitivity. 2D FT image reconstruction of each individual k-space data set gives *m*×*n* number of M-EPI images.

Figure 1 depicts a diagram of the M-EPI pulse sequence and the de-multiplexing of signal into *m* by *n* k-space data sets each of which subsequently undergoes 2D image reconstruction. The pulse sequence begins with *m* temporally sequential excitation pulses to produce signal in multiple adjacent slice planes. Using dephasing or defocusing pulses between the excitation pulses separates in time the signal refocusing of different slices within each readout period of the EPI echo train, utilizing the SIR EPI technique. The multi-slice acquisition is further accelerated using a multibanded pulse (MB) for each excitation leading to an image that is the composite of the simultaneously excited *n* slices for each of the consecutively applied *m* pulses in the SIR approach. The spatial encoding inherent in the phase array receiver coils allows mathematical separation of the composite image to *n* distinct slices by post-processing. Therefore, each single-shot multiplexed EPI sequence generates *m*×*n* slices for a single contrast preparation period. The overall time reduction, however, is somewhat less than *m*×*n*-fold in time to cover the same volume due to the echo train lengthening by SIR. Echo train shortening achieved by reducing the phase encoding steps by factor *R* using partial parallel acquisition techniques such as SENSE [11] or GRAPPA [10] and their derivatives, which is referred to as acceleration, or the use of partial Fourier sampling [9] can also be incorporated in this sequence and were employed in this study.

## Results

**Figure 2:**
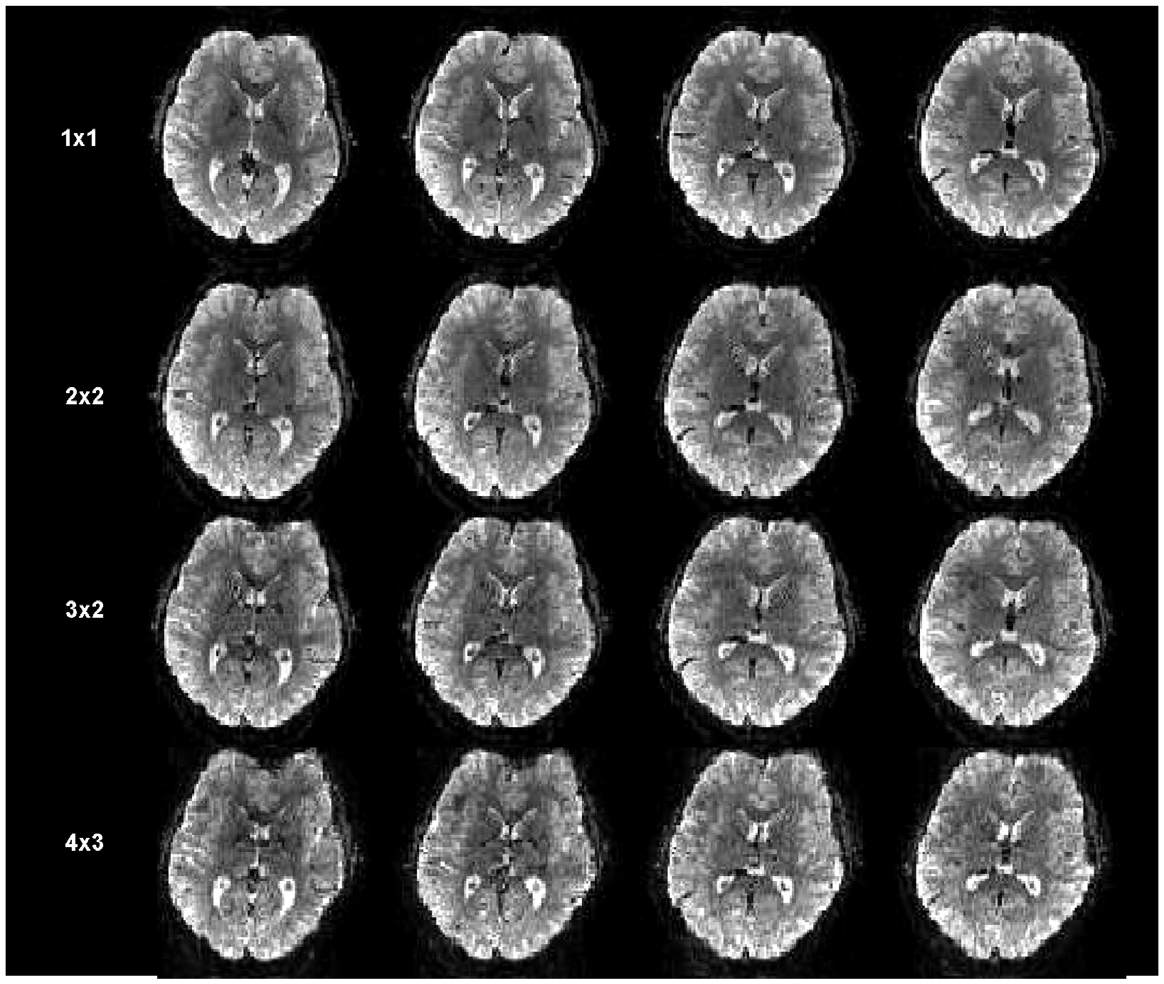
Images at 3 Tesla, comparing 4 adjacent slices out of the total 60 slices at 2mm isotropic resolution covering the entire brain. Each row of images was obtained with a different pulse sequence and slice acceleration, producing 1, 4, 6 and 12 slices from the EPI echo train. The *m*x*n* parameters (SIR× MB) are shown.

Figure 2 shows results for M-EPI performed at 3 Tesla using different “slice acceleration factors” (*m*×*n*) defined as the total reduction in number of echo trains compared to a multi-slice EPI acquisition. Subsets of the complete datasets (60 slices, TE/40 ms, 2 mm isotropic resolution, 96×96 matrix, 1680 Hz/pixel) are shown (for full data sets see Fig. S1a-d). The factor *m*×*n* ranged from 4 to 12. Distortions increased in the frontal cortex near air sinus regions of high susceptibility with the use of higher m-factor as shown in the first column of images in Fig. 2. The average time to acquire an image (for 2 mm acquisitions) is 72 ms (1×1), 23 ms (2×2), 18 ms (3×2), 10.5 (4×3) ms, which is the minimum TR in Table S1 divided by the number of slices. These images were acquired with fully relaxed magnetization to demonstrate feasibility and calculate g-factors from the MB accelerations (see Table S2). However, in practice these acquisitions would be used to reduce the TR, and thus, SNR would also potentially be reduced. Consequently in fMRI studies, when using slice accelerations of 1, 4, and 9 in which TR was shortened from 2.5 s to 0.8 s and 0.4 s, respectively, the estimated relative SNR values were: 146 (1×1), 122 (2×2), 96 (3×3). Here the acquisition times per slice (TR divided by the number of slices) used was: 69 ms, 22 ms, and 11 ms, respectively for the 3 mm isotropic resolution acquisitions. Table S1 shows the minimum possible TRs for the different M-EPI accelerations. Note that all acquisitions used fat saturation pulses, which have a shared effect on multiple images in M-EPI, adding 13 ms, 3.2 ms, and 1.5 ms to the average time per slice for the 1×1, 2×2, and 3×3 accelerations (for the 3 mm resolutions), respectively.

**Figure 3:**
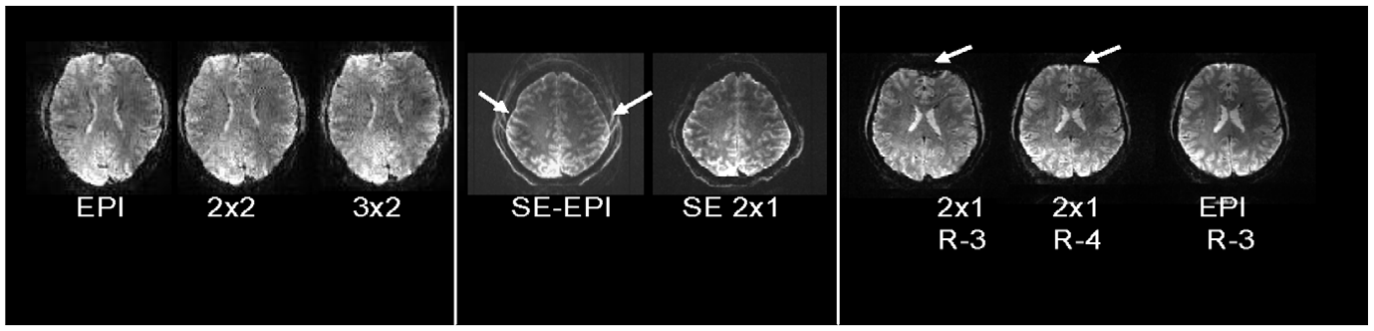
M-EPI images acquired at 7 Tesla. **left**) M-EPI with 1.5 mm isotropic resolution comparing 1, 4 and 6 images per echo train utilizing different multiplexing. **middle**) SE EPI has chemical shift artifact (arrows) that is normally removed with fat-saturation RF pulses or using different pulse lengths for the excitation and the refocusing pulse; the SE M-EPI images have inherent absence of the fat ghost artifact and require half as many refocusing pulses to substantially reduce SAR. **right**) By increasing parallel imaging from R-3 to R-4, the echo train shortened to overcome SIR lengthening to remove the artifact (arrow) with similar appearance to EPI with R-3 and similar echo train length.

Although the SNR decreased with higher slice accelerations and faster TRs (see Table S2), it is significant that the combination of time-multiplexing with SIR and spatial-multiplexing with MB did not result in any *additional* losses than the implementation of each one separately. Further, the SIR acquisition alone did not impose any SNR losses directly, which is attributed to the net signal energy remaining constant in the longer echo trains of SIR at a fixed TE. Echo train lengthening was in part compensated for by reducing the preceding delay time to leave TE of the central k-space point unchanged. Predictably, image geometric distortions occurred with greater off-resonance phase errors in the echo train, which could be corrected by data post processing (e.g. [25]) and echo train shortening methods (e.g. by partial parallel acquisition which directly reduces the distortions). Fig. 3 shows, at 7 T, how off-resonance effects are mitigated by changing the parallel imaging reduction factor, the *R*-factor, from 3 to 4, countering the SIR lengthening effect. Maximal tolerable *R*-factor improves with higher B_0_ [26], [27] and depends on the RF coil design (e.g., [28]).

**Figure 4:**
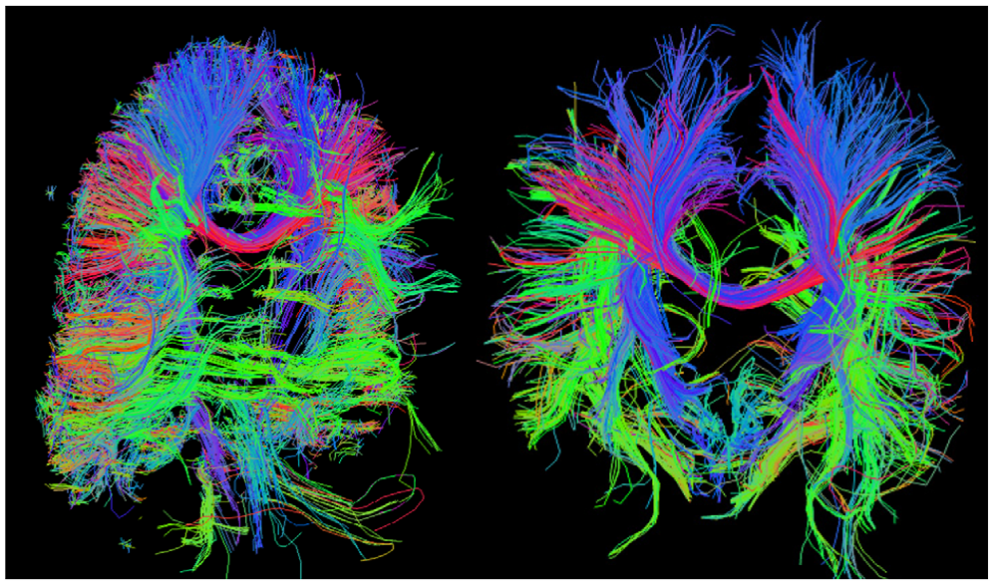
Neuronal Fiber tracks generated using the M-EPI sequence. Two projections of the 3D diffusion spectrum image (DSI) reveal large scale neuronal connections in major white matter pathways. 3T Diffusion acquisition parameters: DSI using b-maximum of 4500 s/mm^2^ and 256 b-value encodings; Imaging parameters: SIR2 × MB2, 3 mm resolution, total acquisition time 8.5 min., TR/2000 ms and signal bandwidth 2604 Hz/pixel.

Figure 4 illustrates an example of the use of this sequence to obtain diffusion spectrum images (DSI) [29] for neuronal fiber tracks, a time-demanding form of high angular resolution diffusion imaging (HARDI) [30]. 3T DSI data were acquired using a maximum b-value of 4500 s/mm^2^ utilizing 256 b-values in the twice-refocused diffusion encoding scheme (48). Imaging parameters: TR/TE 2000 ms/124 ms, *m*×*n* = 2×2, 3 mm resolution and total acquisition time: 8.5 min. To acquire the same diffusion acquisition parameters without slice accelerations would require ∼3.5 times longer scan time. A thorough quantitative evaluation and optimization of the M-EPI sequence is beyond the scope of this manuscript, however, we did compare probabilistic fiber orientation estimation as it depended on the different *m*×*n* accelerations for a HARDI acquisition. Note that we sampled identical diffusion directions for each acceleration and did not match the total acquisition time. We found that although with some accelerations fiber orientation estimation did not perform as well, due to any corresponding SNR decreases in the accelerated acquisitions, the acquisition time reductions (2–4 times faster) may more than offset this by allowing for either shorter scan times or increased coverage of q-space. These results are summarized in Table S3.

Resting state networks (RSNs) are patterns of spontaneous fluctuations that are coherent *within* functional networks and distinct *across* different functional networks [31], [32]. RSNs are seen in BOLD fMRI data through the same neurovascular coupling that allows task- or stimulus-induced brain activity to be imaged, and can be found in all grey matter (any single point in the grey matter being found in one or more RSNs); they are therefore an effective way to investigate the relative merits of the different acquisitions. We identified the same set of RSNs in all 3 acquisitions, and then compared the RSN fluctuation amplitudes (in terms of percent BOLD signal change) and effective SNR (quantifying this as a t-test that divided the amplitude by the standard error of the noise).

**Figure 5:**
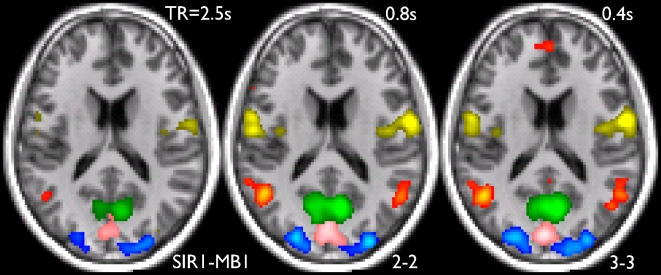
Five example RSNs (resting state networks) from the 3 different 10-minute resting FMRI acquisitions, from subject 2. The color overlays are z-statistic images, thresholded at Z = 4 in all cases. In this central axial slice (Z = +18 mm in MNI152 standard space) the RSNs shown cover visual areas, the default mode network and a sensori-motor network.

Figure 5 shows example results from independent component analysis (ICA) of the resting fMRI datasets. The color overlays show 5 example RSNs (coded in different colors) from the 3 different acquisitions of subject 2, presented as z-statistic images (from a multiple regression against a 100-component cross-TR decomposition of the datasets—see Methods for details), thresholded at Z>4 in all cases. In this central axial slice, the RSNs shown cover visual areas (pink/blue/green), the default mode network (red) and a sensori-motor network (yellow). It is clear that the accelerated sequences display higher functional SNR than the unaccelerated dataset. Additional RSNs from subject 2 are shown in Fig. S3.

**Figure 6:**
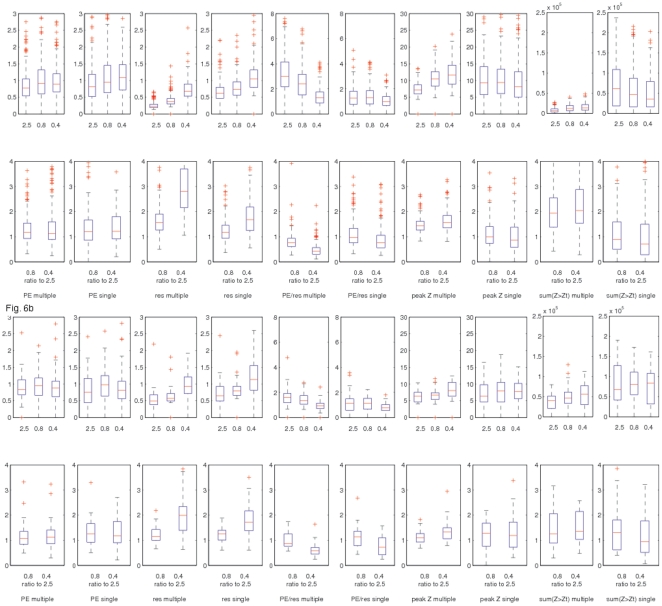
Boxplots over all RSNs (excluding the artefactual ICA components) and over all 3 subjects, quantifying various image statistics. The top row in each sub-figure shows a separate boxplot for each of the three TRs, and the bottom row shows the ratio of the 0.8 s and 0.4 s values to the 2.5 s values, the ratio being calculated separately for each RSN (and each subject) before feeding into the boxplot. Fig. 6a shows the results from a 100-dimensional ICA decomposition across all 3 TRs (separately for each subject) and Fig. 6b shows the results from a 10-dimensional regression of previously published large-scale RSNs from a separate study. For each regression type (multiple or single) and for each RSN map, we have a ‘mixture-model-corrected’ Z-stat image (see Methods). The two main measures we take from that Z-stat image are the peak value and the sum of all Z-stat values over a threshold. For the voxel having the peak Z-stat, we also report the parameter estimate (‘PE’, or RSN amplitude, shown as a % signal change) and the size of the regression residuals. Finally we also show the ratio of these two measures, which contains similar information to the Z-stat (i.e., CNR), but without taking into account the temporal DOF.

These differences are quantified in Figure 6. The boxplots are over all RSNs (i.e., excluding the artefactual components) and over all 3 subjects. The top row in each sub-figure shows a separate boxplot for each of the three TRs, and the bottom row shows the ratio of the 0.8 s and 0.4 s values to the 2.5 s values, the ratio being calculated separately for each RSN (and each subject) before feeding into the boxplot. Fig. 6a shows the results from a 100-dimensional ICA decomposition across all 3 TRs (separately for each subject) and Fig. 6b shows the results from a 10-dimensional regression of previously published large-scale RSNs from a separate study [33].

### Peak Z

In the multiple-regression, 100dim analysis, the median ratio of the 0.8 s and 0.4 s data to the 2.5 s is 1.44 and 1.56 respectively. For single-regressions, these ratios fall to 0.99 and 0.87. In the 10dim analyses, the ratios are 1.10 and 1.34 for multiple-regression, and 1.27 and 1.19 for single-regression. Hence the main result is that for single-regression analyses, the peak Z-stat is quite similar across TRs, but for multiple-regression, the peak Z is 56% higher at the shortest TR compared with the longest. This improvement lessens at lower dimensionality (i.e., 10 RSN regressors instead of 100). We discuss below where this improvement comes from.

### Sum (Z>Z_threshold_)

These results reflect the size (spatial extent) of the estimated RSNs, as well as the statistical significance. In the multiple-regression, 100dim analysis, the median ratio of the 0.8 s and 0.4 s data to the 2.5 s is 1.94 and 2.04 respectively. For single-regressions, these ratios fall to 0.89 and 0.72. In the 10dim analyses, the ratios are 1.26 and 1.35 for multiple-regression, and 1.30 and 0.95 for single-regression. Hence the main result is that for single-regression analyses, the sum-of-Z-stats (size × significance) is quite similar across TRs, but for multiple-regression, the sum-of-Zstats is 100% higher at the shortest TR, compared with the longest (with this improvement lessening at lower dimensionality).

### Amplitude (‘PE’)

These values are expressed as the standard deviation of each RSN's timeseries (estimated at the peak-Z voxel), as a percentage of the mean signal level. As expected, there is not a great variation in RSN amplitude across the different TRs, regression methods and dimensionalities (range 0.75–1.09), with a slight increase in amplitude apparent in low-TR data, when looking at the ratios of the 0.8 s and 0.4 s data to the 2.5 s (range 1.06–1.25).

### Noise (regression residuals)

These values are expressed as the standard deviation of the regression residuals (estimated at the peak-Z voxel), as a percentage of the mean signal level. The residuals are considerably higher (a factor of up to 2.8) in the low-TR data, because the raw SNR per timepoint acquired is reduced with shorter TR. (It is not until the Z-stats are considered, which take into account the increased number of samples in the low TR data, that the low-TR CNR is seen in fact to be as good as or better than the unaccelerated data.) As expected the multiple-regressions have lower residuals than the single-regressions, and the higher-dimensionality has lower residuals than the lower-dimensionality.

In addition to improved sensitivity, the higher temporal resolution allowed for a richer analysis of the temporal dynamics of RSNs than has previously been possible; this will be presented separately in a more complete study and is beyond the scope of this paper.

## Discussion

The effect of simultaneously using two multiplexing schemes in the pulse sequence allowed unprecedented numbers of 2D images to be acquired simultaneously in one echo train without physical limitations from specific absorption rate (SAR) or echo time (TE). Slice acceleration by the SIR approach requires far fewer time consuming readout gradient rise times that can dominate EPI readout trains and thus it achieves greater efficiencies given *m* echoes instead of a single echo are refocused and sampled with each gradient switching. Another efficiency gain is in fMRI where normally EPI sequences have a delay period before the echo train to maximize BOLD contrast and the lengthened SIR echo train extends into this period without increasing TE and signal decay. SIR is ultimately restricted by the obligatory echo train lengthening and eventually the increasing TE delay (see Text S1 for more discussion). The MB technique, on the other hand, does not affect TE but is limited by the ability to encode spatial information by the RF coil array alone, and potentially by SAR, especially at high fields; the SAR limitation arises because MB pulses increase the power quadratically with the number of slices excited relative to a single band pulse, when the same general pulse form is employed to accomplish single *versus* multiple slice excitation *and* the TR is reduced correspondingly by the multiband factor. The limitation of each technique to achieve slice acceleration can thus be significantly ameliorated by combining them; the resultant sequence effectively attains multiplexing in the temporal and spatial dimensions.

We anticipate overall improvements in imaging at higher *mxn* accelerations with several ongoing optimizations, such as different geometry coil arrays with more independent channels, higher amplitude gradients for shortening the echo spacing, pulse sequence optimizations (e.g. ramp sampling, which was not implemented for the current data), and improved image reconstruction algorithms. The SIR lengthening of the EPI echo train increases the sensitivity to distortion, as seen in Fig. 2, which would become intolerable at very high SIR factors; however, the value of using additional SIR acceleration is clearly beneficial in the situation where the length of the echo train (including the effect of parallel imaging along the phase encoding direction) is significantly less than both T_2_* and the inverse of the maximum frequency range over the image FOV. Whether these demands are met depends on factors including spatial resolution, gradient ramp times, available read gradient strength and ramp sampling. The MB slice acceleration does not lengthen the echo train or echo spacing and therefore does not worsen EPI image distortion in regions of susceptibility gradients. However, effects of residual aliasing, as with conventional parallel imaging, could introduce spurious activations. We have previously evaluated this and shown it to be non-significant up to MB factors of 4 [24] (see also Fig. S2). On the other hand, the SIR technique must be used judiciously with higher performance gradients and parallel imaging to shorten the echo train duration, reducing artifacts and distortions. At 7T, higher parallel imaging factors and closer echo spacings, made possible with the stronger and faster switched read gradients, permit encoding of higher spatial resolutions with minimized distortions and susceptibility artifacts, benefiting the SIR technique (see Fig. 3).

In principle the readout train length could have been varied for the different SIR acquisitions, resulting in lower bandwidths for the less accelerated data, while matching the distortions of the data with the higher SIR factors. This would yield a relatively higher SNR for the lower SIR factor acquisitions and potentially better fMRI performance. However, this would also come at the expense of longer TRs and potentially longer TEs, which could adversely affect fMRI performance. Further, given the fact that the highest readout bandwidths were *not* used for the lower SIR factor acquisitions, and because of echo spacing limitations imposed by the software, significant gains in SNR would not have been achieved and would have come at the expense of other parameters.

Higher magnetic fields provide higher image SNR and supra-linear increases in functional signals associated with the microvasculature (e.g. [34], [35]), which can result in major gains in accuracy and functional contrast-to-noise ratio (CNR) in fMRI. However, tissue heating arising from RF energy absorption (SAR) also increases with the higher magnetic fields and imposes limitations, particularly for the MB technique if 180° refocusing pulses are needed for either high accuracy spin echo fMRI [36] or for diffusion imaging. We have found that SAR decreases with time-multiplexing in SE SIR EPI due to the fewer refocusing pulses needed (reduced by the SIR factor), compared to conventional SE-EPI. Additionally, in SIR acquisitions at 7 T, there was an absence of signal from lipid and consequently an absence of chemical shift artifact (Fig. 3), eliminating the need for fat suppression pulses which are often applied in SE EPI. This further alleviated the power deposition problem at 7 T. The absence of fat artifact results from shifting the fat signal out of the image plane by using an *m*-times weaker slice-selective gradient on the 180° refocusing pulse compared to the excitation pulse in order to simultaneously refocus signal in adjacent SIR slices. The weaker slice-selective gradient concurrently causes a greater displacement (D) of fat signal on the slice axis, out of the excitation planar region, by D = δB_0_(Gs)^−1^ where δ is the chemical shift and B_0_ the magnetic field strength and Gs is the slice-selective gradient. Therefore, fat suppression in M-EPI at high field is inherently achieved due to the weaker Gs of the refocusing pulses in SIR, without using fat saturation pulses that increase SAR. In SE EPI sequences, a lengthening of the RF refocusing pulse relative to the excitation pulse [37] is used to similarly eliminate fat signal. Other approaches involve changing the polarity of the slice-selective gradient (Gs) during refocusing [38]. The inherent fat suppression in M-EPI, without the use of fat saturation pulses, reduces SAR which is critical for whole brain scanning at high fields.

A comparison of M-EPI to other fast imaging techniques, particularly 3D acquisitions, can be considered. Since 3D imaging utilizes 2 phase encode directions, reductions in the echo train can be attained by undersampling along the two dimensions, reducing significantly the spatial encoding times. As such, it was shown that 3D acquisition times could be reduced to levels near or faster than what is obtained with 2D multi-slice imaging, greatly improving the performance of 3D functional images over 2D [15], [39]. Such an approach, however, suffers a significant penalty in SNR per image of the volume covered, irrespective of the TR employed, since undersampling the k-space reduces SNR by factor (*R_1_R_2_*)^1/2^ where *R_i_* is the reduction in the k-space points sampled along phase encoding direction *i*. In contrast, an SNR penalty per image of the volume covered at any given TR is not inherent to the slice acceleration approach described in this paper. 3D PRESTO SENSE [18], [19] (a multi-shot 3D EPI technique) has also been proposed to significantly reduce the acquisition using the dead (TE preparation) time between the RF pulse and the subsequent readout to apply the next excitation pulse, and then separating the readouts by echo shifting. The utility of this technique depends on the required TE; at higher fields, however, due to the shorter T_2_* and consequently the shorter TEs used, there is typically very little dead time to obtain the shifted echoes from the previous excitation. Furthermore, lower SNR due to the use of lower flip angles [19], [39], [40], as well as any inherent TE delay in PRESTO [41], would be sub-optimal for HARDI imaging. With a 2D PRESTO sequence run at shorter TRs, smaller excitation flip angles create magnetization components which must be spoiled so that overall SNR is even less competitive with 2D EPI based techniques [19].

There are substantially greater efficiency gains using M-EPI in HARDI as the large time spent on diffusion encoding (typically 60 ms–100 ms) is shared for multiple slices and not repeated for each slice. Consequently, higher spatial and diffusion resolutions that may be prohibited by EPI scan times as long as an hour, could be achieved with M-EPI in much shorter times, tolerable by most subjects. Alternatively, the shorter scanning times for a complete data set can be utilized to acquire several such complete data sets in the time that would have taken to acquire a single EPI based data set. This approach would have the advantage of increasing the probability of achieving a complete data set even if some are rendered useless due to motion. An example of tractography data extracted from a DSI HARDI measurement obtained using the M-EPI sequence is presented as a demonstration of feasibility. A quantitative comparison of gains relative to performance criteria such as resolution of crossing fibers is beyond the scope of this work at this stage and will be pursued and presented separately taking into account trade-offs in SNR, acquisition time and acceptable levels of distortions.

### Higher sampling rate in fMRI

It is known that resting state functional connectivity studies benefit from higher sampling rates to adequately sample undesirable respiration and cardiac effects [42], while for event-related task fMRI, faster sampling could allow for a better characterization of the hemodynamic response. In this study, the faster sampling rate yielded a larger number of total time points, improving the spontaneous neural fluctuations' Z-scores (effectively the SNR for these neural processes) in certain analysis scenarios, despite the lower SNR “per image” due to the faster TR acquisitions. This higher statistical power garnered could in turn be used to substantially shorten the total acquisition time (making clinical applications more feasible) and/or increase the spatial resolution (allowing finer distinctions between different functional regions).

For single-regressions, the increase in noise (i.e. decrease in raw SNR per single image) at low-TR is nearly balanced by the statistical advantage of the increased number of samples (timepoints), resulting in peak Z-stats being similar across TRs. This is as predicted by the Bloch equations assuming thermal noise; the reduction in gradient-echo signal, as a function of TR and T_1_, is well-balanced by the sqrt(N_timepoints_) increase, over this range of TR values, with a predicted improvement of ∼15% in raw peak Z-stats at 0.4 s, compared with 2.5 s.

However, for multiple-regressions, the short TR data performs significantly better than the longer TR. This is partly because of the reduction in residuals when using all regressors together, a factor which benefits the short TR more than the long TR data, with some group-ICA components (and hence the final regressors) modelling some physiological noise processes with better sampling of these effects at lower TR. It is also partly because the reduction in temporal degrees-of-freedom caused by the use of a large number of regressors impacts more on the data with fewer timepoints. Finally, given that statistical significance in a multiple-regression is driven by a regressor's *unique variance* (compared with all other regressors), the result also reflects the fact that the low-TR data contains improved information with which to discriminate the different components from each other. This effect is expected to rise as the dimensionality rises (because of the rise in correlation between regressors).

Both single-session ICA and the application of dual-regression to map group-ICA results into individual datasets are effectively based around a multiple-regression, and hence benefit from the increase in temporal information shown here to be valuable in low-TR data. However, methods related to single-regression, such as seed-based correlation, will not see this advantage, nor will model-based analysis in a task-FMRI experiment (although artefact removal would probably be improved in both scenarios when using lower-TR data). Nevertheless, even in the ‘worst case’ scenario, the Z-stats (effective CNR) of low-TR data are at least as good as higher-TR data (and, in other scenarios, are considerably better).

The M-EPI approach described here may have a significant advantage over conventional segmented 3D EPI approaches in that the k-space data used to form images results from a single RF excitation. The ultimate determinant of functional CNR in most fMRI applications are temporal fluctuations due to physiologic sources and not the thermal SNR of a single image (e.g. [43], [44], [45]) and these fluctuations form the basis of R-fMRI. However, multi excitation schemes such as 3D EPI or segmented 2D EPI introduce additional unwanted perturbations since these temporal fluctuations are encountered while the k-space is being covered, affecting the resultant image in a complicated way. (e.g. [7], [8]). Further, M-EPI could have significant advantages in scan time reduction in other applications (anatomical or functional) that depend on magnetization preparation, such as inversion recovery based sequences.

In conclusion, the novel Multiplexed EPI pulse sequence significantly increased the temporal resolution of whole brain fMRI, and substantially reduced diffusion scan times. As such, this methodology can be used for expanding and enriching the functional and anatomic information obtained from MRI. Further, the reduced scan times may help the clinical acceptance and translation of functional MRI protocols and HARDI neuronal fiber track imaging.

## Methods

The imaging protocol used for human studies was approved by the institutional review board (IRB) at the University of Minnesota. Ten subjects were scanned using this IRB approved protocol. Each of the subjects provided informed written consent prior to participating in the research. Imaging at 7 Tesla was conducted on a Siemens (Erlangen, Germany) system with a 90 cm bore magnet from Magnex Scientific (currently Agilent Technologies), equipped with a head gradient set (AC84, Siemens) operating at up to 70 mT/m with a slew rate of 333 mT/m/ms. The 7T RF coil consisted of a single transmit channel with 16 receiver coils. Imaging at 3 Tesla used a standard commercial scanner (Siemens Trio) equipped with 40 mT/m gradients with a slew rate of 200 mT/m/ms and utilized the 32 channel head receiver coil system.

The separation of multibanded spatially multiplexed signals was performed as previously described [24] with a modified strategy. As calibration data, an acquisition with matched SIR factor, and a MB = 1 excitation was obtained. The sum of the slices that are to be acquired with MB>1 are used to define a GRAPPA-type projection operator (matrix) which is calculated over a 7×7 region [46]. A plethora of different 7×7 regions are selected from the sum of the slices, and each is matched to a single point. This generates a sufficiently large set of data points to estimate the 7×7 number of elements in the matrix needed for separating the frequency-multiplexed signals.

The RF coil employed for the 7T composed of 16 azimuthally-distributed loops dedicated for signal reception. Despite a lack of distinct coil distribution along the z-axis, at 7T, some z-encoding is nonetheless achieved due to the complex 3-dimensional heterogeneities in RF distribution in the human head. A 4-port driven TEM coil surrounding the receiver array was employed for RF transmission. The 3T array coil employed a distributed coil design along the lines presented by Wiggins et al [47] et al previously. The body RF coil was employed for RF transmission. We used a 15-degree tilt on the y-axis to capitalize on the two-dimensional distribution of the coils, improving MB de-aliasing.

The optimization of M-EPI is different at 7T and 3T, given the differences in SAR and parallel imaging performance. At 3T the MB SIR sequence acquiring 2 mm isotropic pixels had the following imaging parameters: 1680 Hz/pixel, matrix size 96×96. All sequences used 6/8 partial Fourier, *R_PE_*  = 2, minimum TE = 40 msec, and 60 slices. The TE varied by 2.5 ms between adjacent SIR images. Identical TEs can be obtained by adding blipped Gp pulses between excitation pulses to equally offset the k_0_ of respective SIR images into different readout periods. The M-EPI sequence was used to acquire resting state fMRI (R-fMRI) data, and compared to standard EPI at 3T. 3 mm isotropic resolution images were acquired with: 2604 Hz/pixel, matrix size 64×64, *R_PE_*  = 2, TE = 40 msec, and 36 slices. The minimum TRs for the different acquisitions are summarized in Table S1. Three sequences were employed for the comparison: standard EPI which is equivalent in our designation to M-EPI with *mxn* of 1×1 at TR 2.5 s, and M-EPI with *mxn* of 2×2 at TR 0.8 s, and 3×3 at TR 0.4 s respectively, corresponding to a maximal 18-fold *m*×*n*×*R* acceleration while using flip angles of 90°, and Ernst angles of 60° and 50°, respectively. The average echo spacing, corresponding to a single k-space line, was 0.47 ms, 1.05 ms, and 1.44 ms for SIR (m-factors) 1, 2, and 3, which sampled 1, 2 or 3 slices, respectively, during a single read period. Ramp sampling was used for the normal EPI acquisitions (m = 1), but not implemented for higher SIR/m factors, which would have resulted in reduced echo spacings and more optimized SIR acquisitions. Concomitant MB accelerations did not alter the timing of the readout. These three sequences were employed on each of 3 healthy subjects (at rest with eyes closed) resulting in 9 datasets. The total time for the R-fMRI time series (10 minutes), the spatial resolution (3 mm isotropic with 36 slices), and the TE (40 ms) were kept constant across all acquisitions. The relative estimated SNRs of the 2 mm and 3 mm isotropic images at 3T were estimated using an ROI measurement of mean signal in gray matter divided by mean of air.

The twice-refocused diffusion encoding sequence, first described by Feinberg and Jacob [48], was incorporated into the M-EPI sequence by methods earlier reported using SIR alone [48], [49]. Imaging parameters for 3T diffusion imaging were as follows: DSI using b-maximum of 4500 s/mm^2^ and 256 samples in q-space, SIR2 × MB2, 3 mm resolution, total acquisition time 8.5 min, TR/2000 ms and signal bandwidth 2604 Hz/pixel. The TE was 124 ms, increased by 12 ms in comparison to an otherwise identical EPI based sequence. Images were reconstructed in the TrackVis program [50].

At 7T, the SE EPI images were acquired with isotropic 1.5 mm voxels, with a 128×128 matrix, BW/2400 Hz/pixel. Both the SE-EPI and SIR EPI sequences utilized 90° pulse durations of 2.56 ms and 180° pulse duration of 5.12 ms, with the refocusing pulse lengthened to reduce SAR.

### Resting FMRI Analysis

Analysis was carried out using FSL (FMRIB's Software Library) [51], [52]. The following analysis methodology was applied separately for each of the 3 subjects.

*Preprocessing*: Each 10-minute resting FMRI dataset was corrected for head motion using FLIRT (FMRIB's Linear Image Registration Tool [53]. Temporal drift was removed using a highpass filter of full width 200 s. The mean (over time) image was brain-extracted using BET [54]. FLIRT was used to align the mean brain image from the two accelerated acquisitions to the unaccelerated one. This was in turn aligned to the brain-extracted structural image (T_1_-weighted, 1×1×1 mm^3^), and this was aligned to MNI152 standard space, again using FLIRT. The various affine transforms were combined and all 4D resting FMRI datasets resampled into 2×2×2 mm^3^ standard space. Spatial smoothing of 5 mm full-width-half-maximum was applied.

### Multi-acquisition RSN analysis

RSNs and structured artifacts in the data were identified using MELODIC (Multivariate Exploratory Linear Optimized Decomposition into Independent Components [55], FSL's implementation of ICA (Independent Component Analysis [56]). In order to define RSN and artifactual components that were equivalent networks/artifacts across the 3 acquisitions, we applied the methodology of a “group-averaged” ICA followed by dual-regression of the group-average spatial maps into each of the 3 separate datasets [57]. First, each dataset was reduced to the top 200 principal components using principal component analysis (PCA), the resulting eigenvectors were scaled to have the same overall variance in the 3 datasets, and then temporally concatenated, giving 600 spatial maps containing the strongest signals in the 3 datasets. This was fed into MELODIC, to identify 100 ICA components. The resulting 100 spatial maps represent structured signal present across the 3 datasets. These were then regressed into the 3 separate datasets, in each case resulting in 100 timecourses associated with the spatial maps. These were normalized to unit variance (so that the output from the following stage contains RSN amplitude information), and then regressed back into the data in order to generate 100 spatial maps, which correspond across the 3 datasets, but which are specific to the dataset from which they are generated (by the two regression stages). Components which were identified as artefactual by virtue of their spatial characteristics (in most cases being clearly driven by vascular pulsation rather than being neuronally-related RSNs) were discarded, leaving an average of 62 RSN components per subject.

From the second multiple regression we can investigate the residuals (which will include thermal noise and any remaining artefacts not modelled in the 100 components), the RSN BOLD fluctuation amplitude (or ‘PE’, i.e., parameter estimate in the multiple-regression), and the Z-stats (basically the amplitude normalised by the residuals). We divide both the residual standard deviation and the amplitude by the original mean signal, so that the first 4 columns in the top row of the boxplots are in units of % signal change.

Although these regressions are able to provide Z-stats for comparing effective CNR across TRs, simple ordinary-least-squares regression does not correct for the true (temporal) degrees-of-freedom (DoF) in the data. This is an important issue if the residuals are temporally smooth (auto-correlated), which will be the case if the residuals contain physiological noise, but not for thermal noise. For the former, the temporal smoothness of the residuals will rise as the TR is reduced. If this is not corrected for, it will yield artificially high Z-stats for the low-TR data. To correct for residual autocorrelation we applied mixture-modeling [55] to each Z-stat image. Here the histogram of any given Z-stat image is modelled by the use of a central Gaussian, and separate gamma distributions for the positive and negative parts of the histogram. The central Gaussian fits the ‘null’ part of the histogram, i.e., the effect of the residual noise in the regression. The gamma distributions model the values of the voxels that are actively involved in a given component (RSN). If all Z-stat values in the image are then shifted and rescaled in order to achieve a central null component having zero mean and unit variance, we have achieved a robust correction for the original temporal DoF (because by definition, if these have been correctly handled, a *true* Z-stat image should have these characteristics for the ‘unactivated’ voxels).

In addition to carrying out the regressions as multiple-regressions (i.e., regressing all 100 timeseries into the data simultaneously), we performed separate analyses using single-regressions (i.e., regressing each timeseries into the data one at a time, independently). This results in less specific spatial maps. For example, if one major resting state network is split across 5 of the 100 ICA components, then multiple-regression will return these 5 sub-regions of the network as 5 distinct maps (as each part of the multiple-regression is driven by the unique component of the variance for the relevant timeseries), whereas single-regression will return 5 very similar spatial maps, each of which looks similar to the complete original network. Thus the latter is quite close to the seed-based-correlation [32] approach, particularly for high dimensionalities (and hence small seed regions).

In addition to carrying out the high-dimensional analyses, we also used a set of 10 spatial maps from another study ([33]; 10 RSNs from 36 subjects) as the dual regression input maps. This is typical of an ICA/dual-regression analysis where one seeks to identify a small number of large-scale RSNs, rather than achieving a detailed decomposition. This allows a ‘cruder’ identification of the major RSNs, though does not have a strong analogy with seed-based approaches (as the ‘seed’ in this case is massively extended across a whole large-scale network) and is not very useful in general for further analyses such as network modelling or identifying the breakdown of the gross networks with pathology.

For each regression type (multiple or single) and for each RSN map, we have a ‘mixture-model-corrected’ Z-stat image. The two main measures we take from that Z-stat image are the peak value and the sum of all Z-stat values over a threshold (this gave similar results to the supra-threshold *voxel count*, so we only report the former). The threshold used is derived from the mixture modeling: it is the Z-stat value where the null part of the modeled distribution crosses the ‘activation’ part, i.e., where the probability of being ‘active’ is equal to the probability of being ‘background’ (noise, or null). For the voxel having the peak Z-stat, we also report the effect size (RSN amplitude, shown as a % signal change) and the size of the regression residuals. Finally we also show the ratio of these two measures, which contains similar information to the Z-stat (i.e., CNR), but without taking into account the temporal DoF.

## Supporting Information

Figure S1 **The full 60 image data sets comparing normal EPI (1x1) and the M-EPI data sets (M×N) acquired at 2 mm isotropic resolution (where M is the SIR factor and N is the MB factor).** **a**) Regular EPI (1×1), **b**) (2×2), **c**) (2×3) and **d**) (3×3). Parameters and minimum TR acquisition times are given in Table S1.
(PDF)

Figure S2 **Simulation of multiband aliasing with unaliased slices.** Top row: 4 single band slices acquired as part of a 60 slice single band standard EPI acquisition. Middle row: The 4 slices were then combined (summed) and then separated with the methods used in this paper and as described in Moeller et al MRM 2010, with a SENSE/GRAPPA algorithm. Bottom row: The difference between the MB un-aliased slices and the original slices.
(PDF)

Figure S3 **25 example RSNs from the 100-dimensional group-ICA (followed by dual-regression) analysis of the 3 datasets from subject 2.** The top row shows in blue the BOLD amplitude as a percent signal change, thresholded at 0.4. The bottom row shows in red-yellow the Z-stat (effective CNR) thresholded at 4. The three columns are, from left to right: TR = 2.5 s, 0.8 s, 0.4 s. The "Intensity" values shown refer to the value of the percent signal change or Z-stat at the position of the cross-hair.
(PDF)

Table S1 **The minimum repetition times (TRs) for acquiring whole brain imaging with the specified M-EPI sequence.** Unless noted, the calculations are for 60 slices for the 2 mm acquisitions and 36 slices for the 3 mm. The average time per slice or this case the minimum time per slice is the TR divided by the number of slices.
(PDF)

Table S2 **Results of the g-factors and SNR for the different M-EPI acquisitions.** The 2 mm isotropic acquisitions and SNR values for the different M-EPI were acquired fully relaxed with 90 degree flip angles. The 3 mm resting state fMRI image acquisitions were acquired using flip angles of 90° (1×1), 60° (2×2), and 50° (3×3) with TRs of 2.5 s, 0.8 s, and 0.4 s, respectively.
(PDF)

Table S3 **Results of the probabilistic fiber orientation estimation using diffusion data with different slice accelerations.** 2nd to 1st is the proportion of voxels that had a 2nd fiber orientation above threshold when the first was above threshold. 3rd to 1st is the same proportion applied to 3rd fibers. Dispersion (i.e. orientation estimation uncertainty) is also reported for each fiber as well. Values are reported from white matter only as defined by segmentation of T1 weighted anatomical images. HARDI data were acquired at 3T using 2 mm isotropic resolutions with 71 diffusion directions and a b value of 2000 s/mm2. The total acquisition time was 11 min., 7.5 min., 5.5 min., and 3.5 min. for the 1×1, 2×1, 1×2, and 2×2 accelerations, respectively.
(PDF)

Text S1 Supplemental text.
(PDF)
