# Correction: Multiplexed Echo Planar Imaging for Sub-Second Whole Brain FMRI and Fast Diffusion Imaging

**DOI:** 10.1371/annotation/d9496d01-8c5d-4d24-8287-94449ada5064

**Published:** 2011-09-28

**Authors:** David A. Feinberg, Steen Moeller, Stephen M. Smith, Edward Auerbach, Sudhir Ramanna, Matthias Gunther, Matt F. Glasser, Karla L. Miller, Kamil Ugurbil, Essa Yacoub

The authors would like to add Matthias Gunther as the 6th author. The updated byline is: David A. Feinberg1,2,3*, Steen Moeller4, Stephen M. Smith5, Edward Auerbach4, Sudhir Ramanna1, Matthias Gunther1, Matt F. Glasser6, Karla L. Miller5, Kamil Ugurbil4, Essa Yacoub4

Matthias Gunther is also affiliated with Fraunhafer-Mevis Institute, Bremen, Germany

The updated author contributions are: Conceived and designed the experiments: DAF SMS KU EY. Performed the experiments: DAF SM EA SR MG EY. Analyzed the data: DAF SM SMS EY KLM MFG. Contributed reagents/materials/analysis tools: DAF SM EA SR SMS MG MFG. Wrote the paper: DAF SM SMS KU EY KLM. 

