# Supplementary figures and images for: Multiplexed Echo Planar Imaging for Sub-Second Whole Brain FMRI and Fast Diffusion Imaging
Source: PLoS One. 2010 Dec 20;5(12):e15710. doi: 10.1371/journal.pone.0015710 (PMC3004955; doi:10.1371/journal.pone.0015710)

SIR 1 MB 1

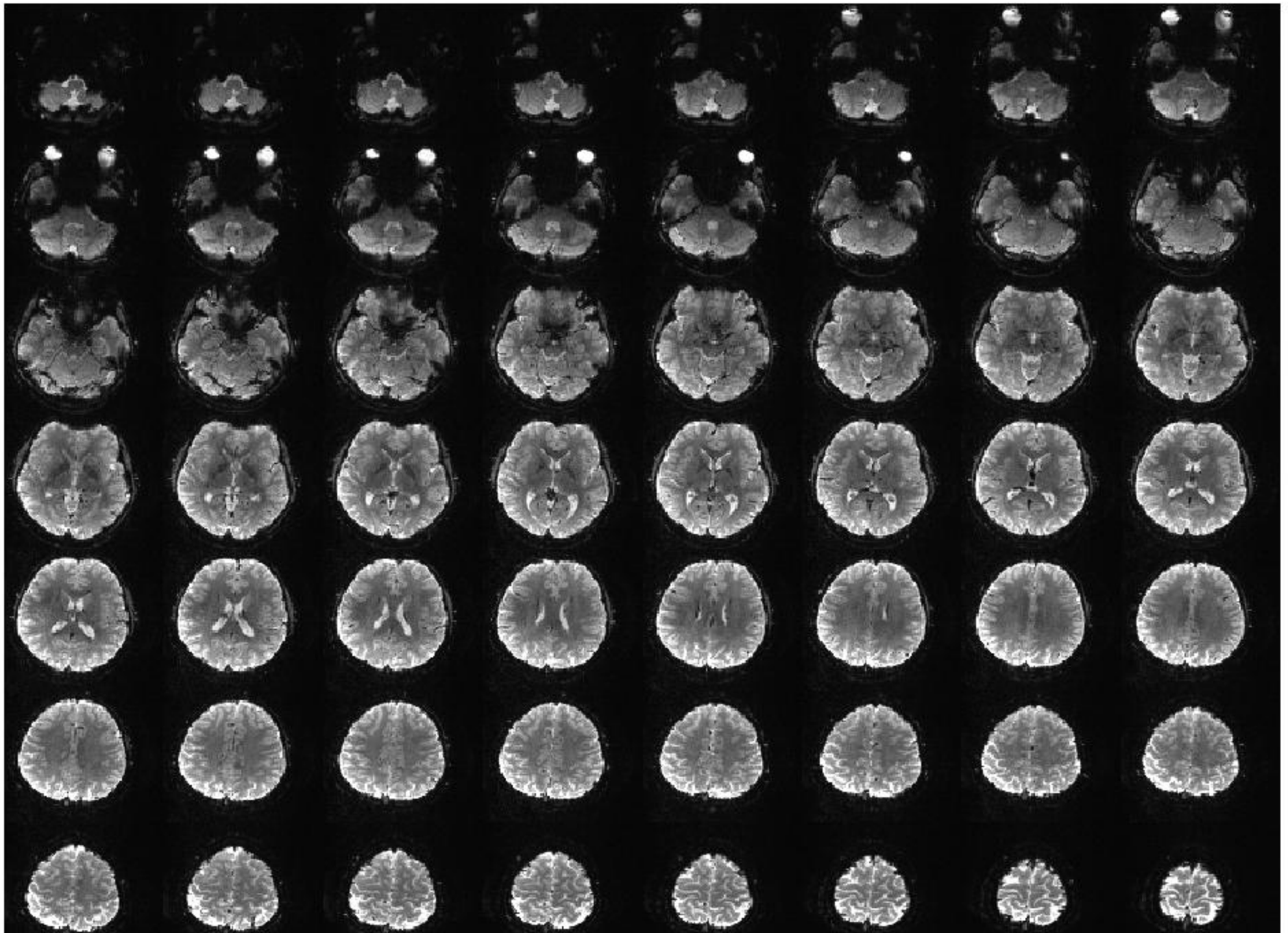

Fig. S1a

SIR 2 MB 2

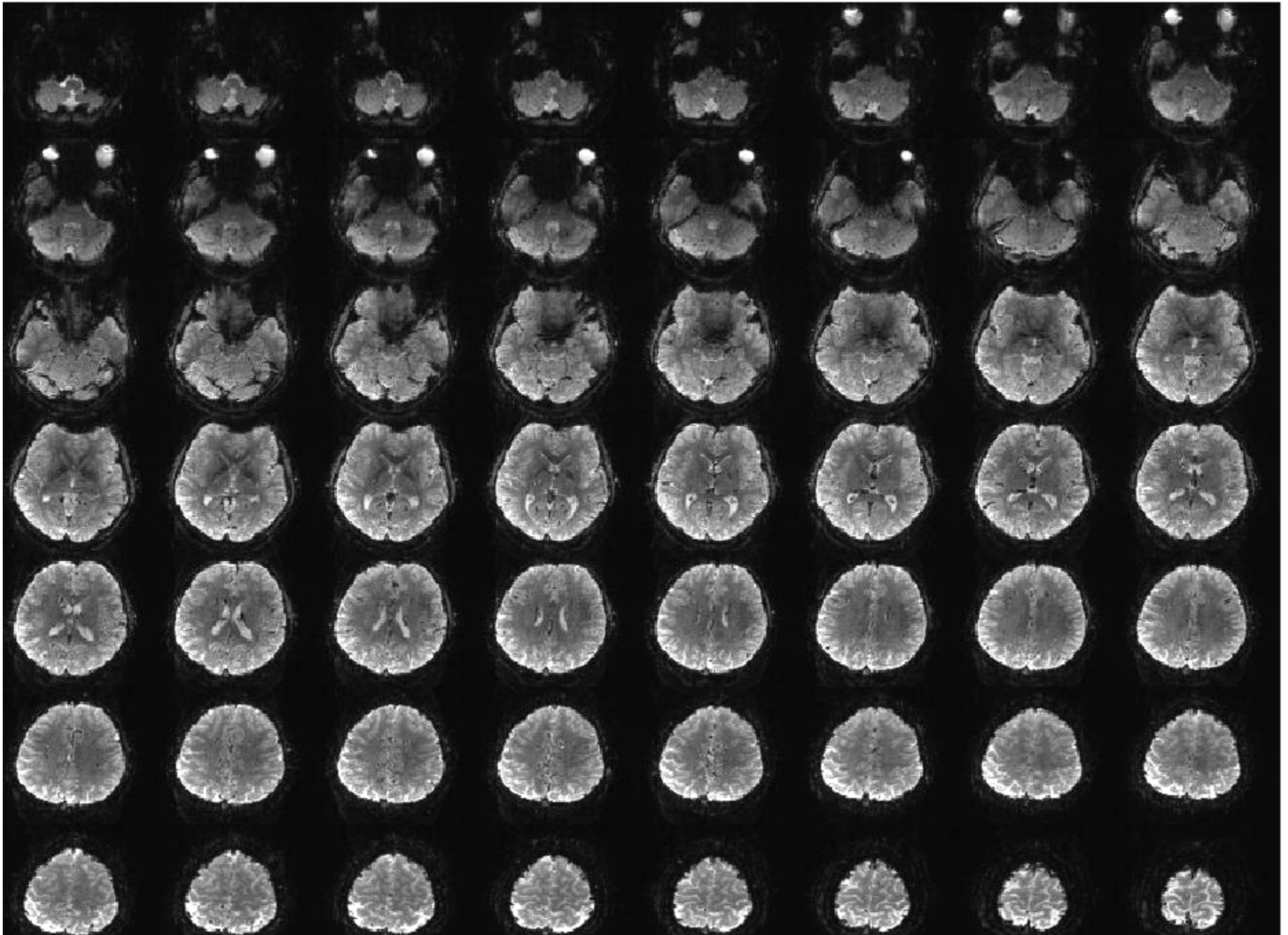

Fig. S1b

SIR 2 MB 3

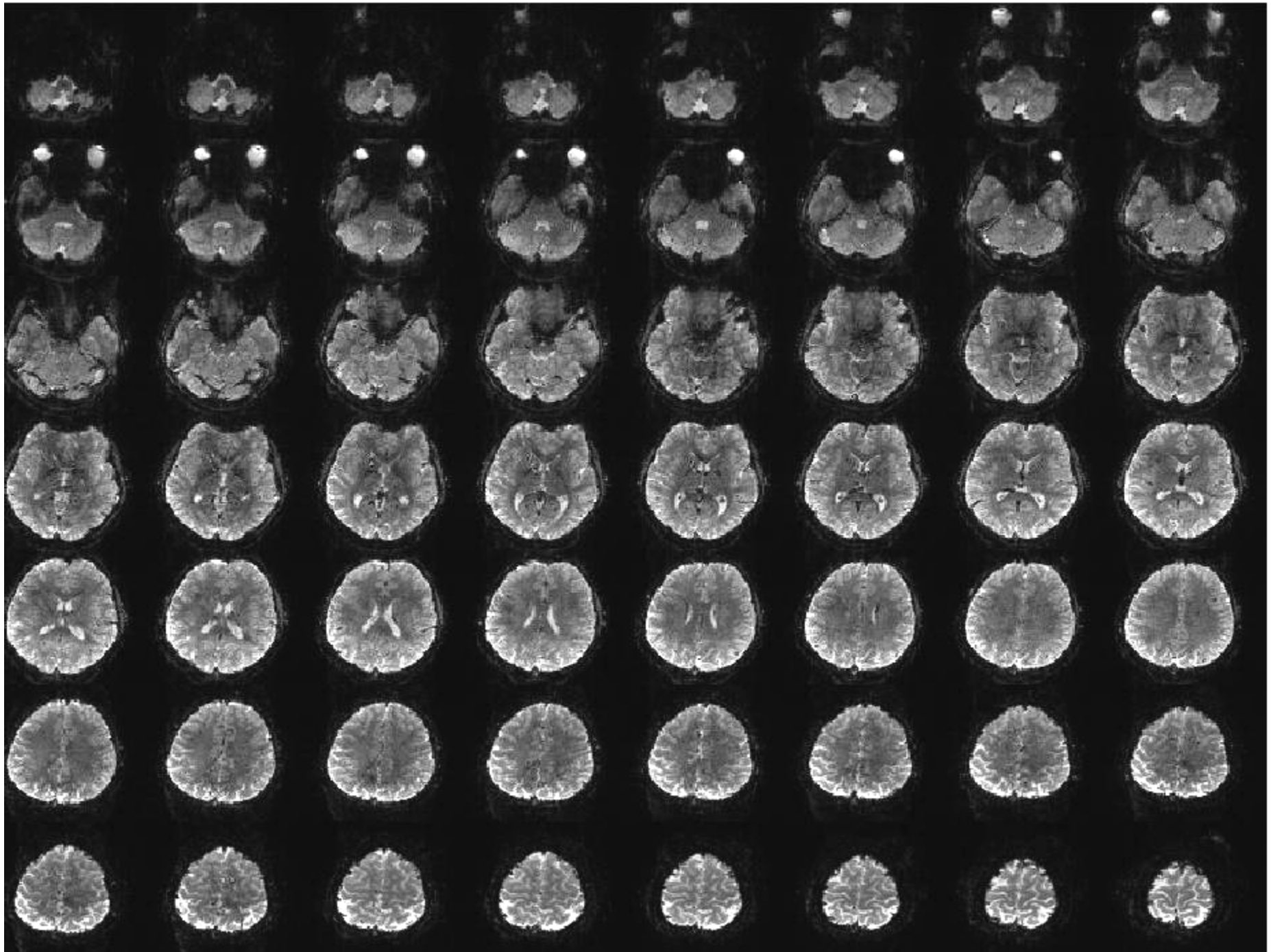

Fig. S1c

SIR 3 MB 3

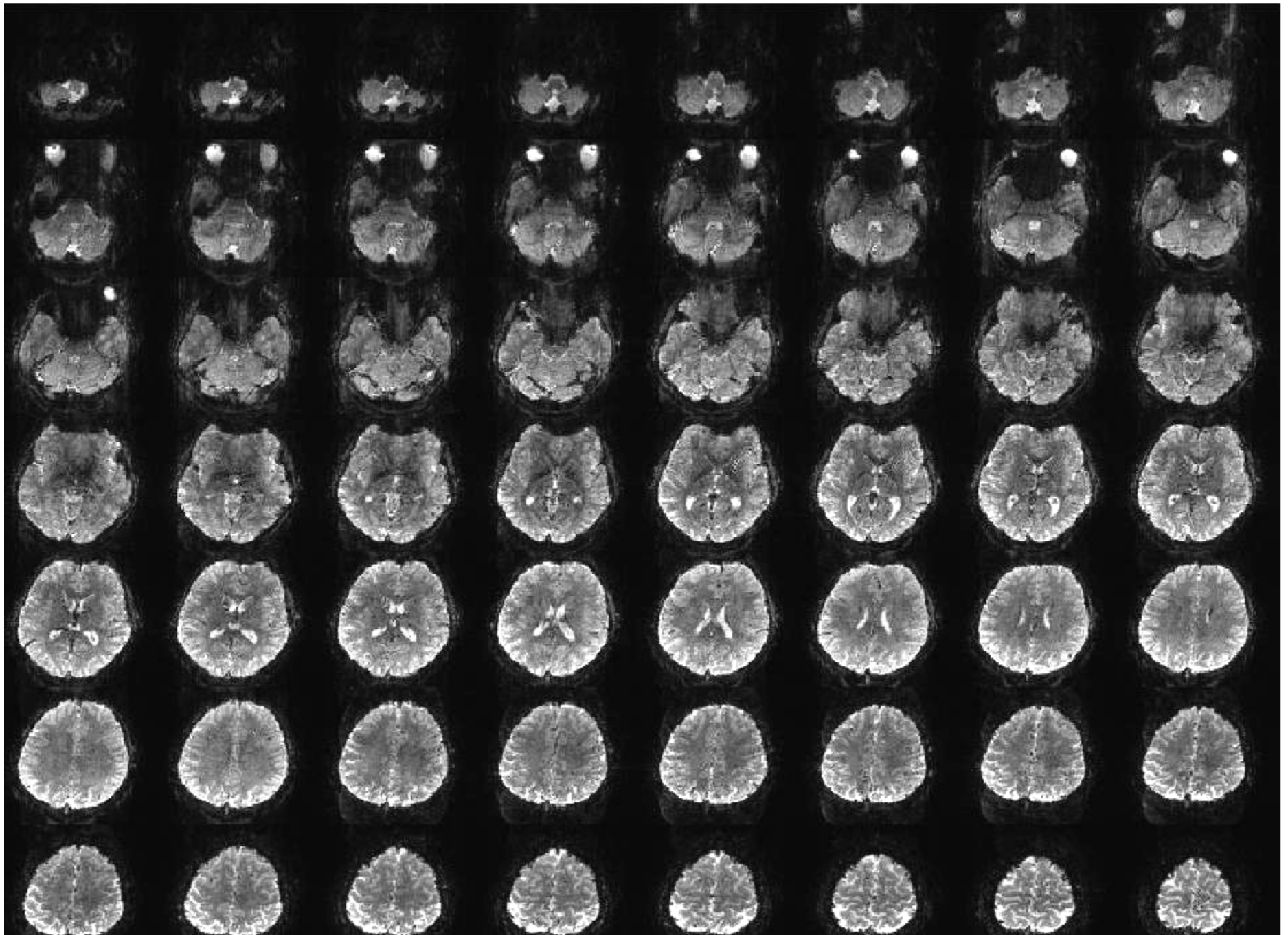

Fig. S1d

Supplement: Figure S1 — The full 60 image data sets comparing normal EPI (1x1) and the M-EPI data sets (M×N) acquired at 2 mm isotropic resolution (where M is the SIR factor and N is the MB factor). a) Regular EPI (1×1), b) (2×2), c) (2×3) and d) (3×3). Parameters and minimum TR acquisition times are given in Table S1. (PDF) [file pone.0015710.s001.pdf]

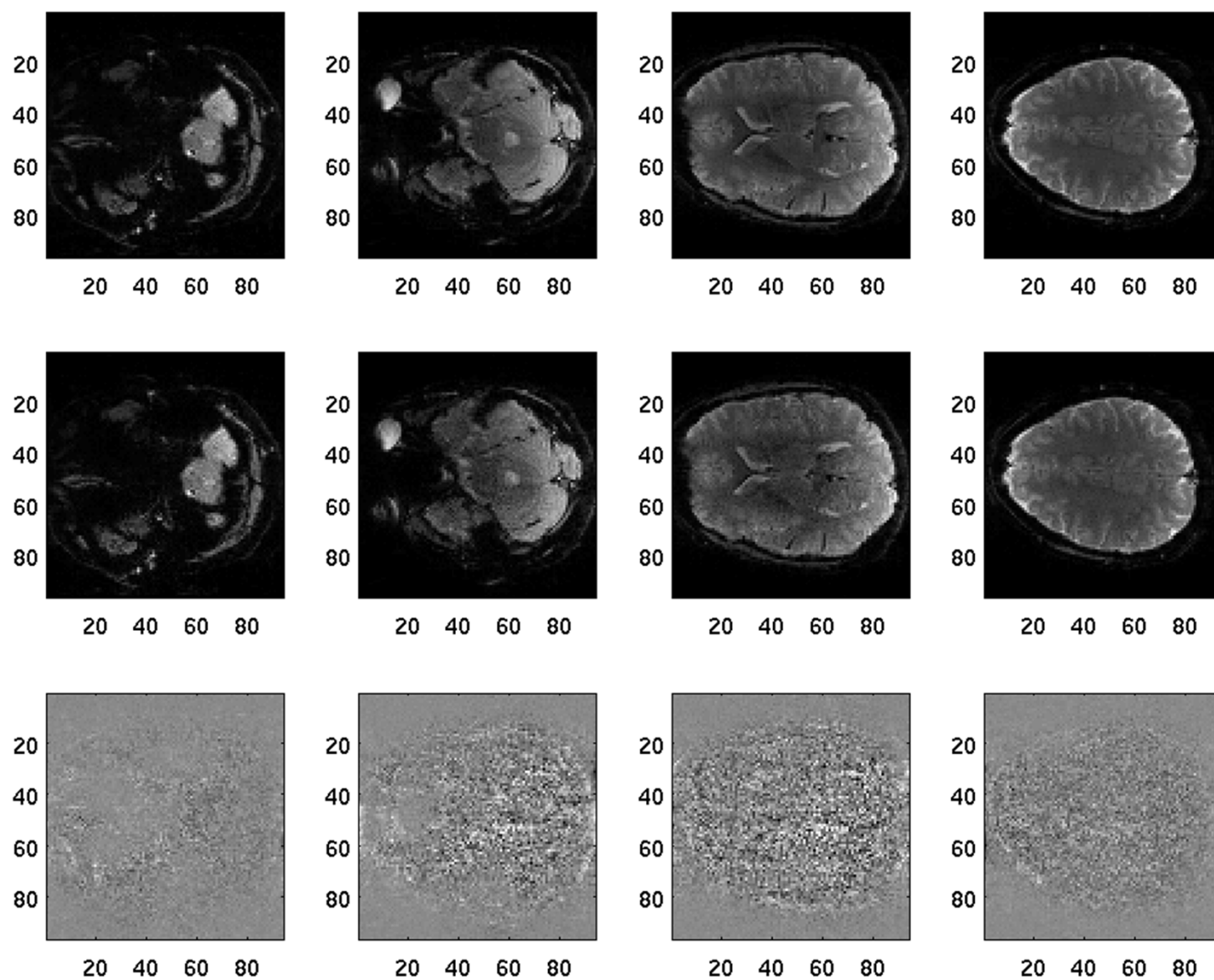

Fig. S2

Supplement: Figure S2 — Simulation of multiband aliasing with unaliased slices. Top row: 4 single band slices acquired as part of a 60 slice single band standard EPI acquisition. Middle row: The 4 slices were then combined (summed) and then separated with the methods used in this paper and as described in Moeller et al MRM 2010, with a SENSE/GRAPPA algorithm. Bottom row: The difference between the MB un-aliased slices and the original slices. (PDF) [file pone.0015710.s002.pdf]

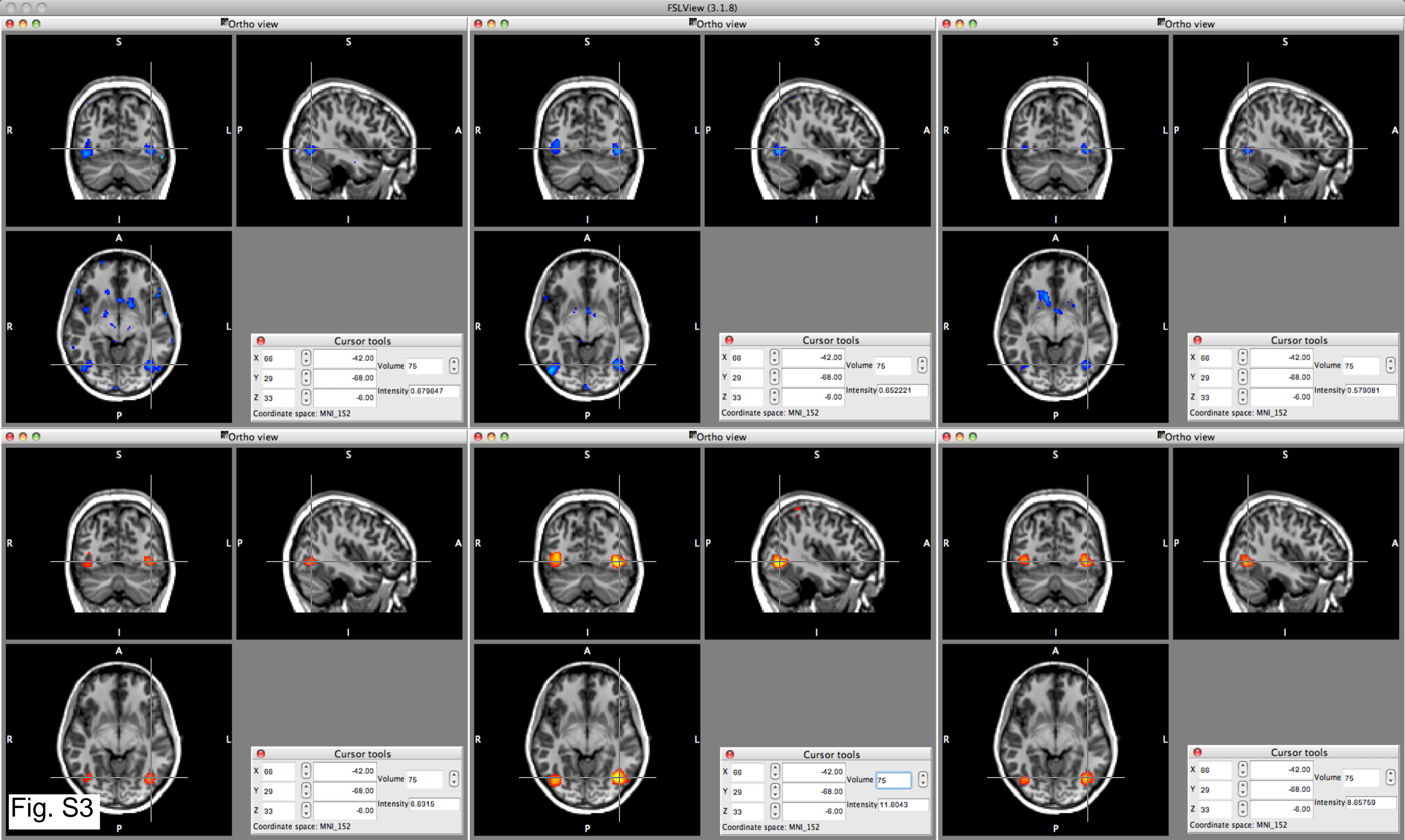

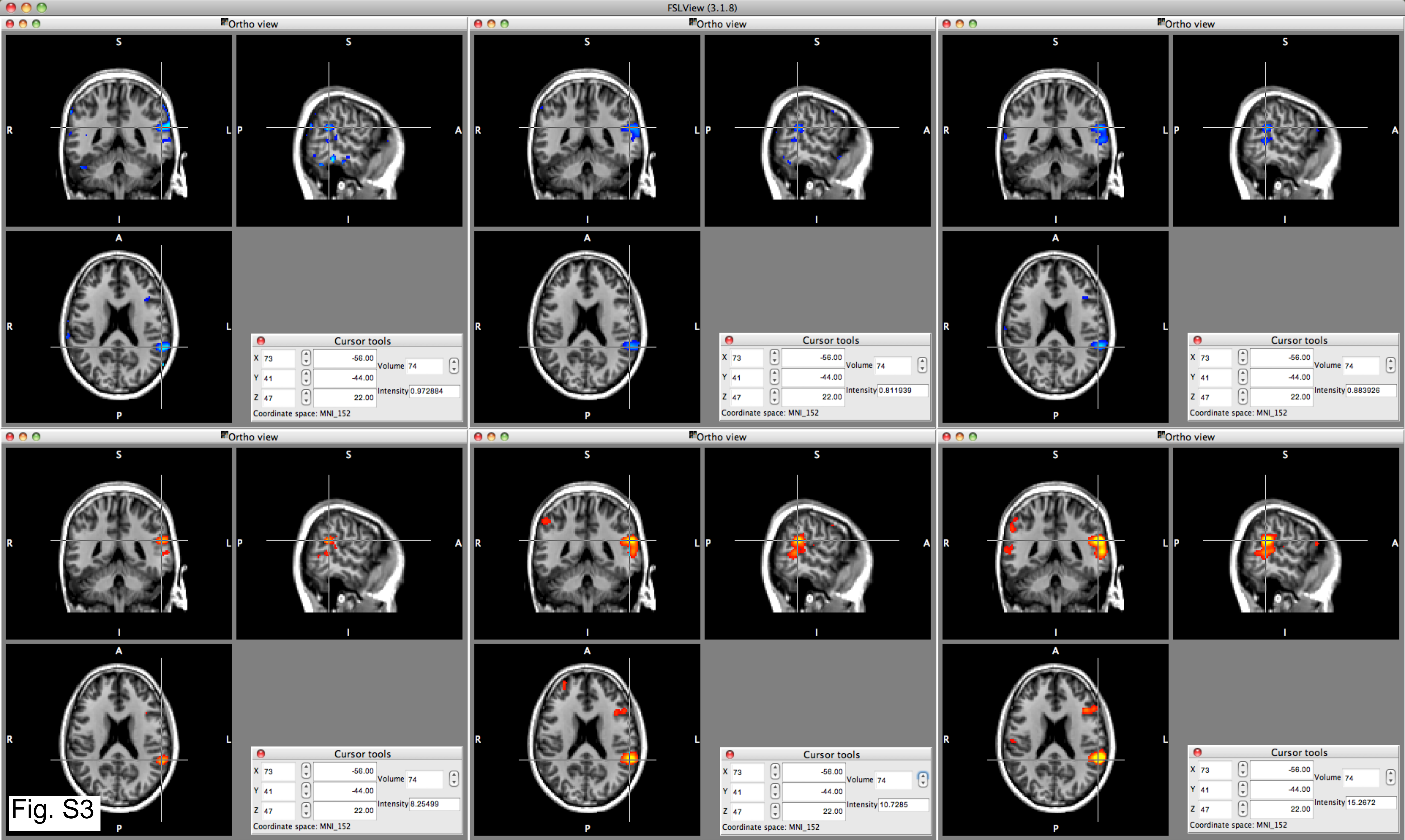

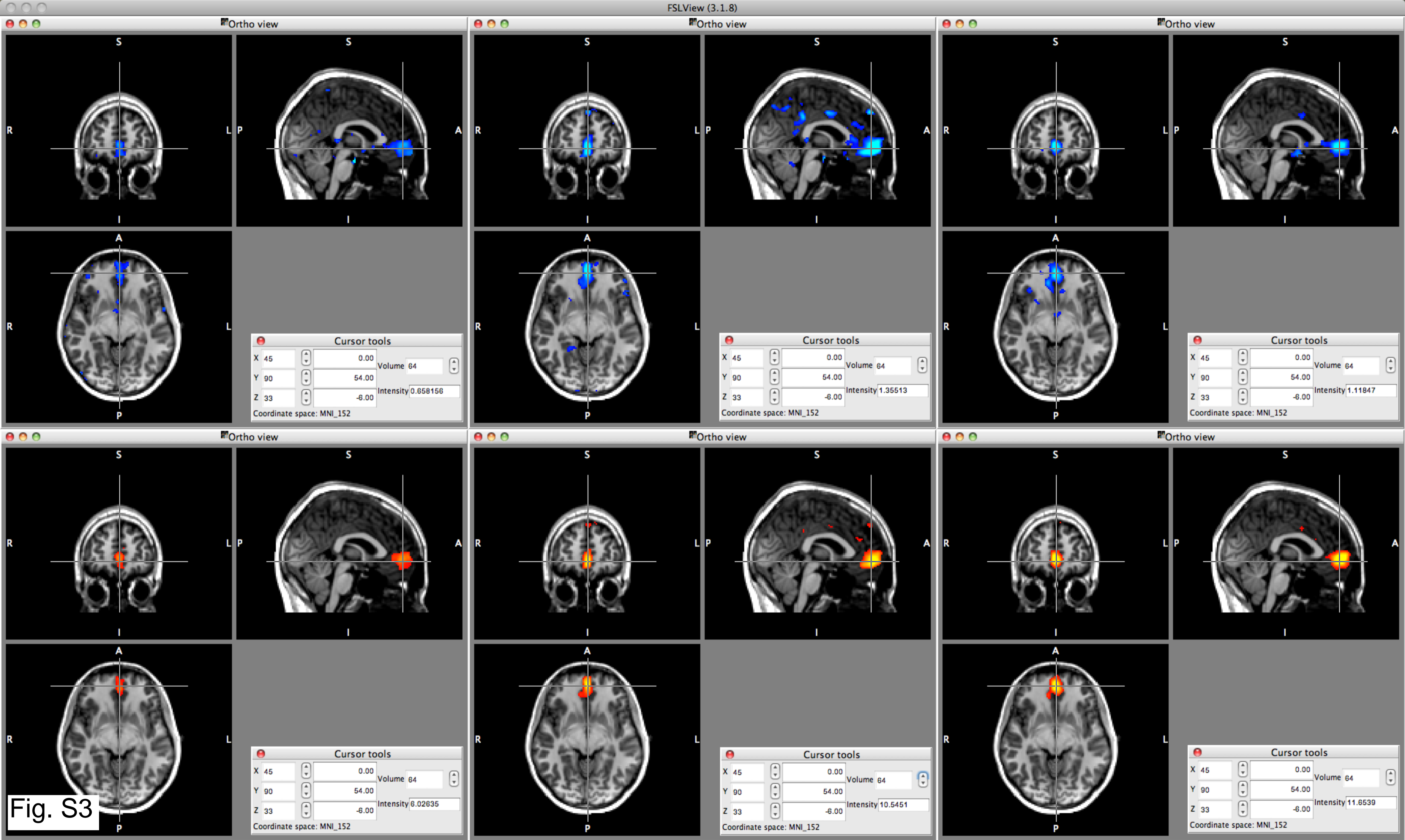

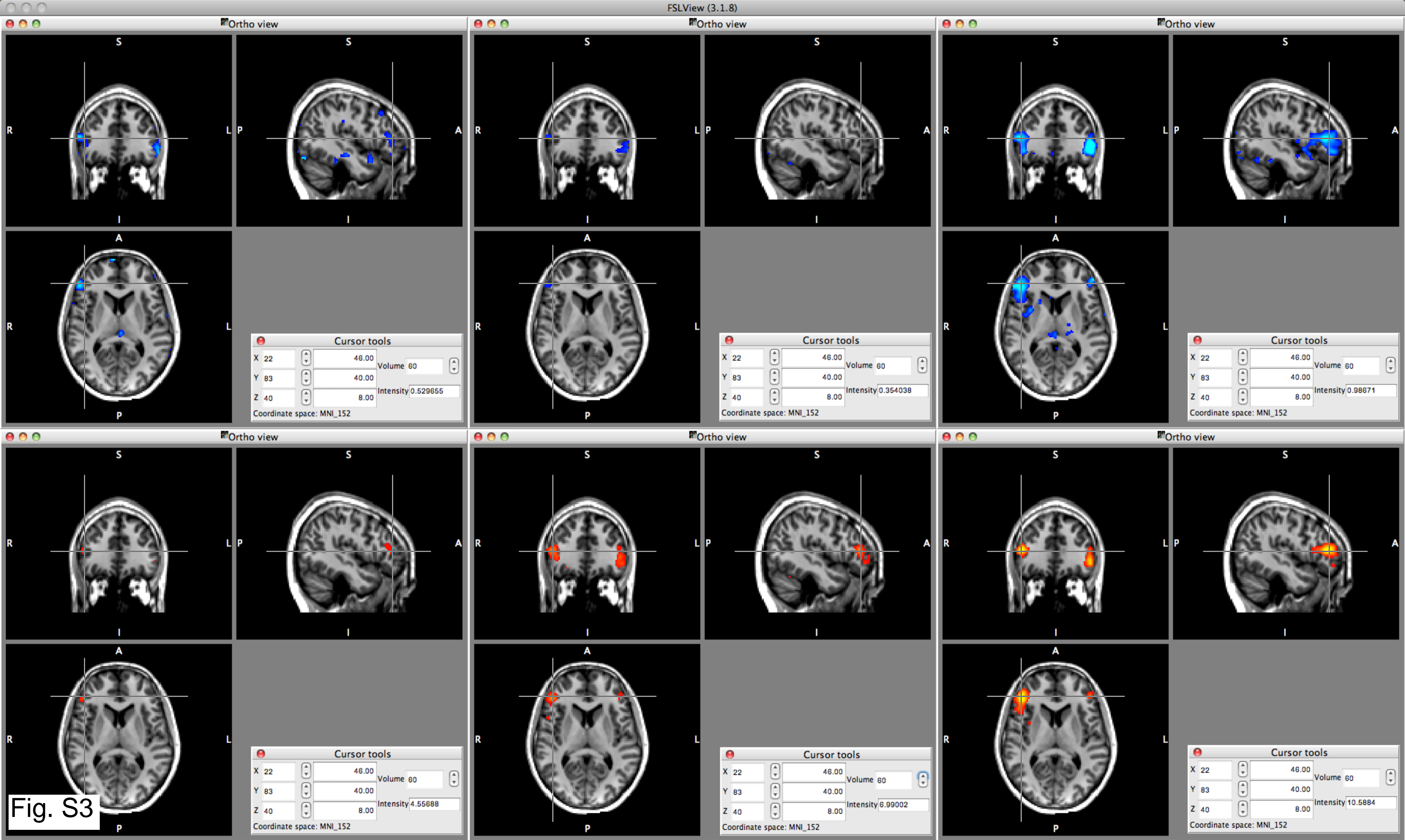

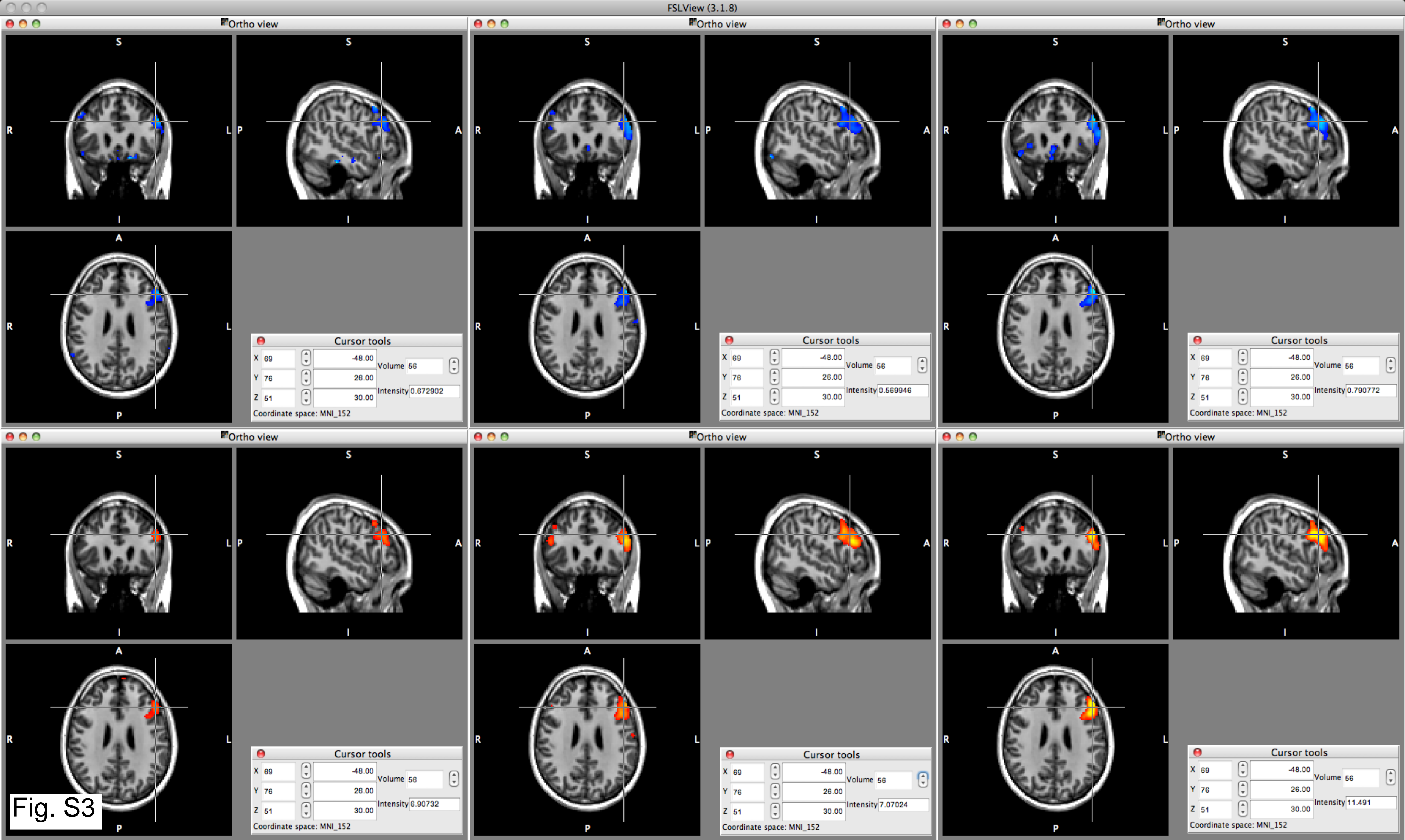

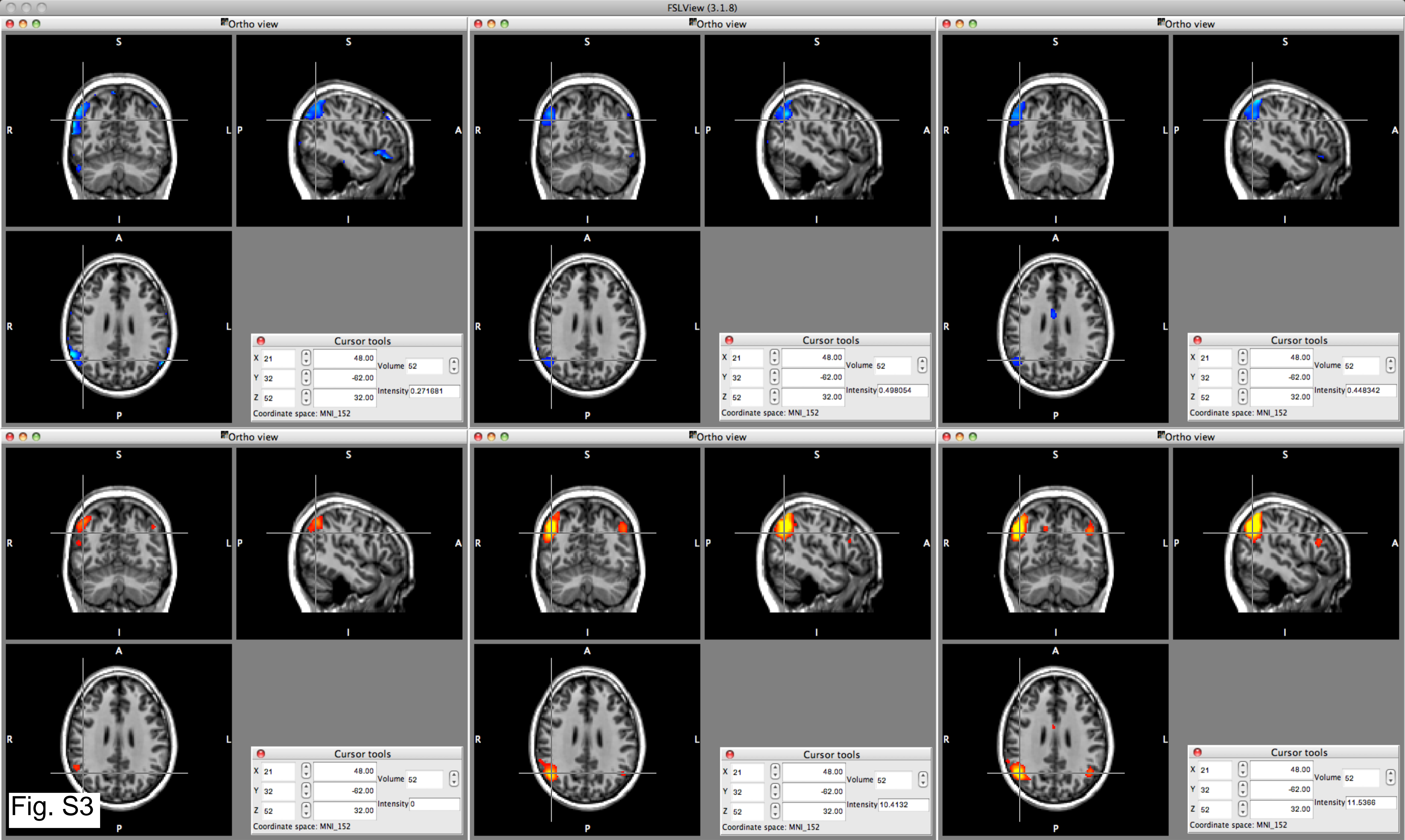

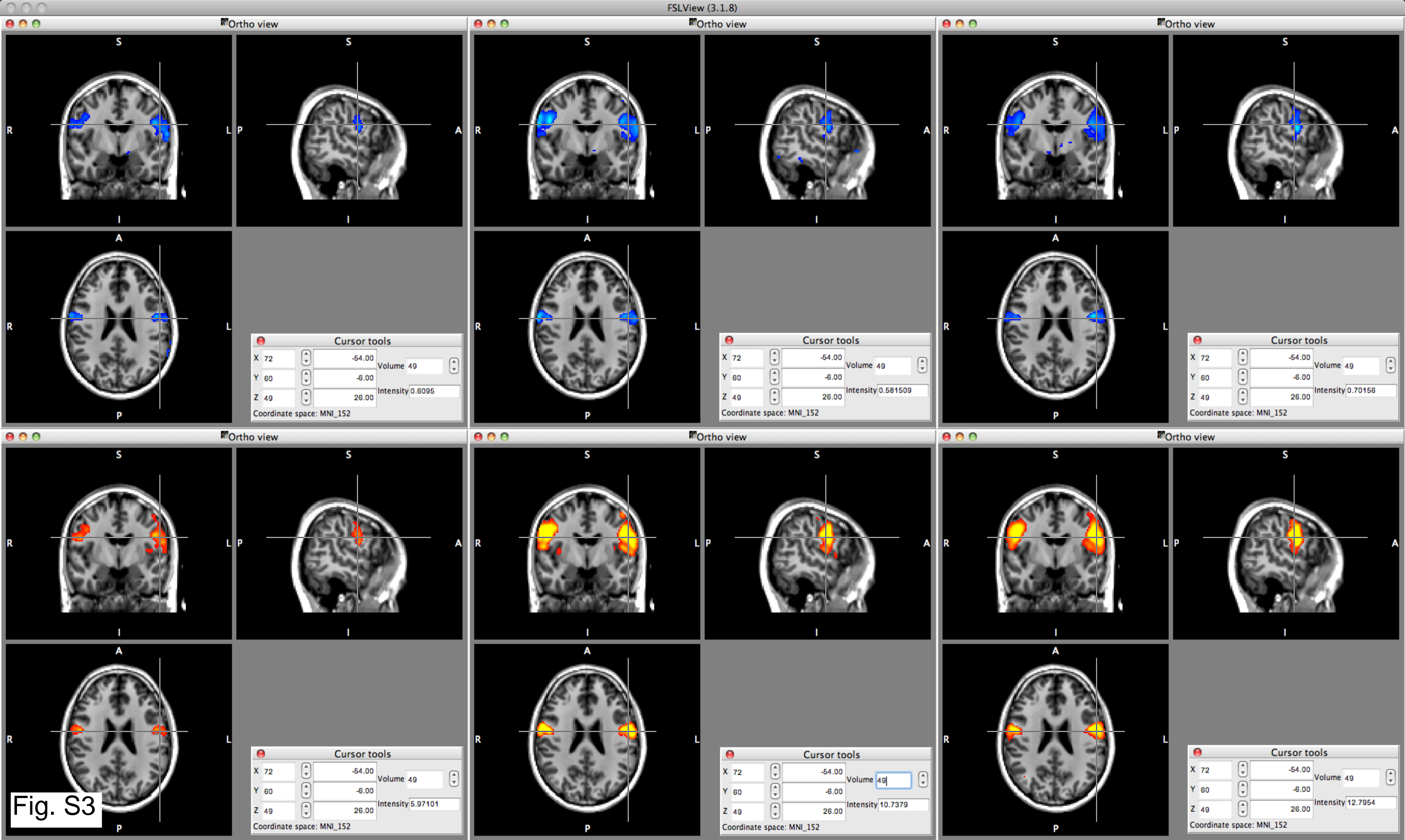

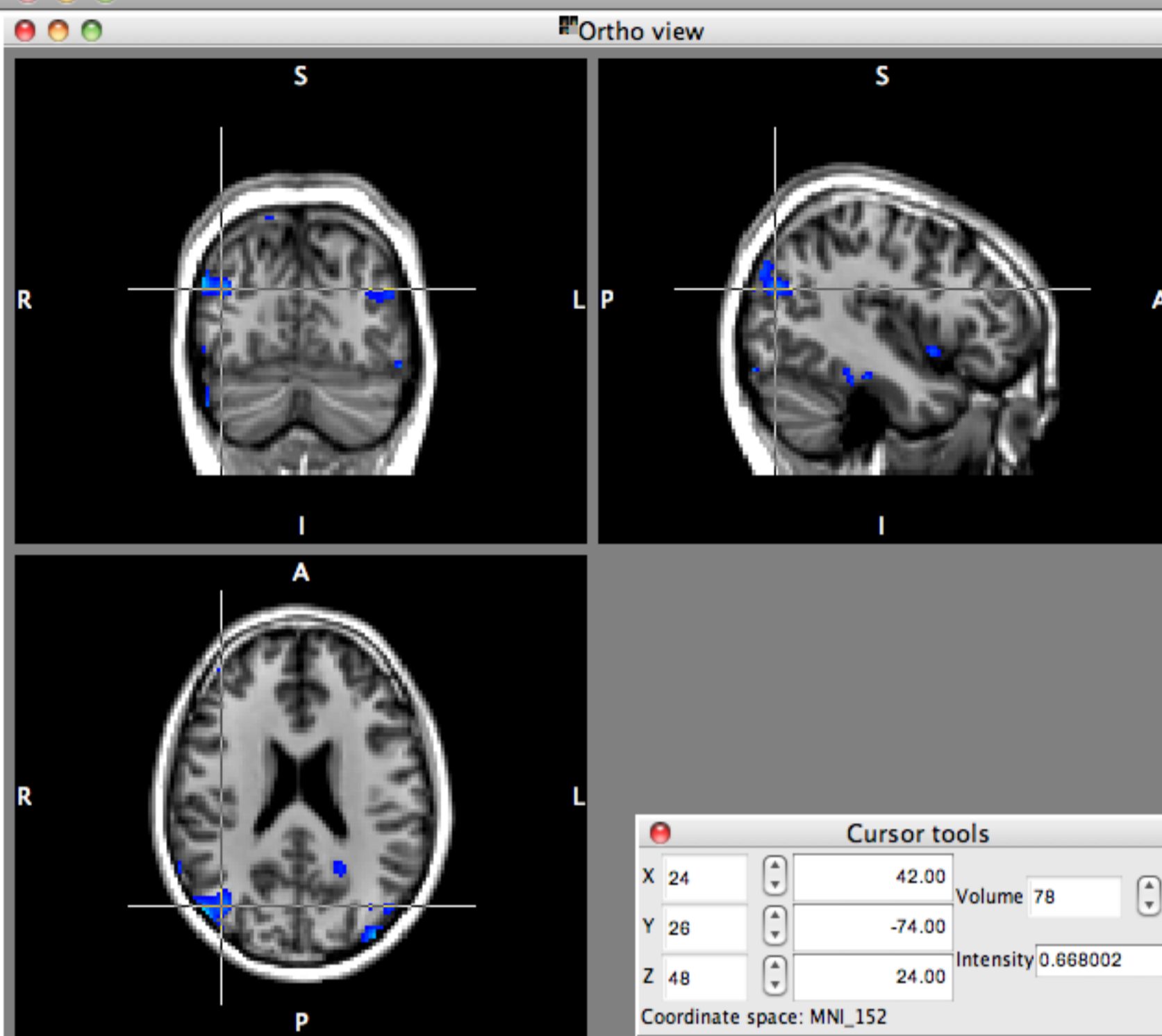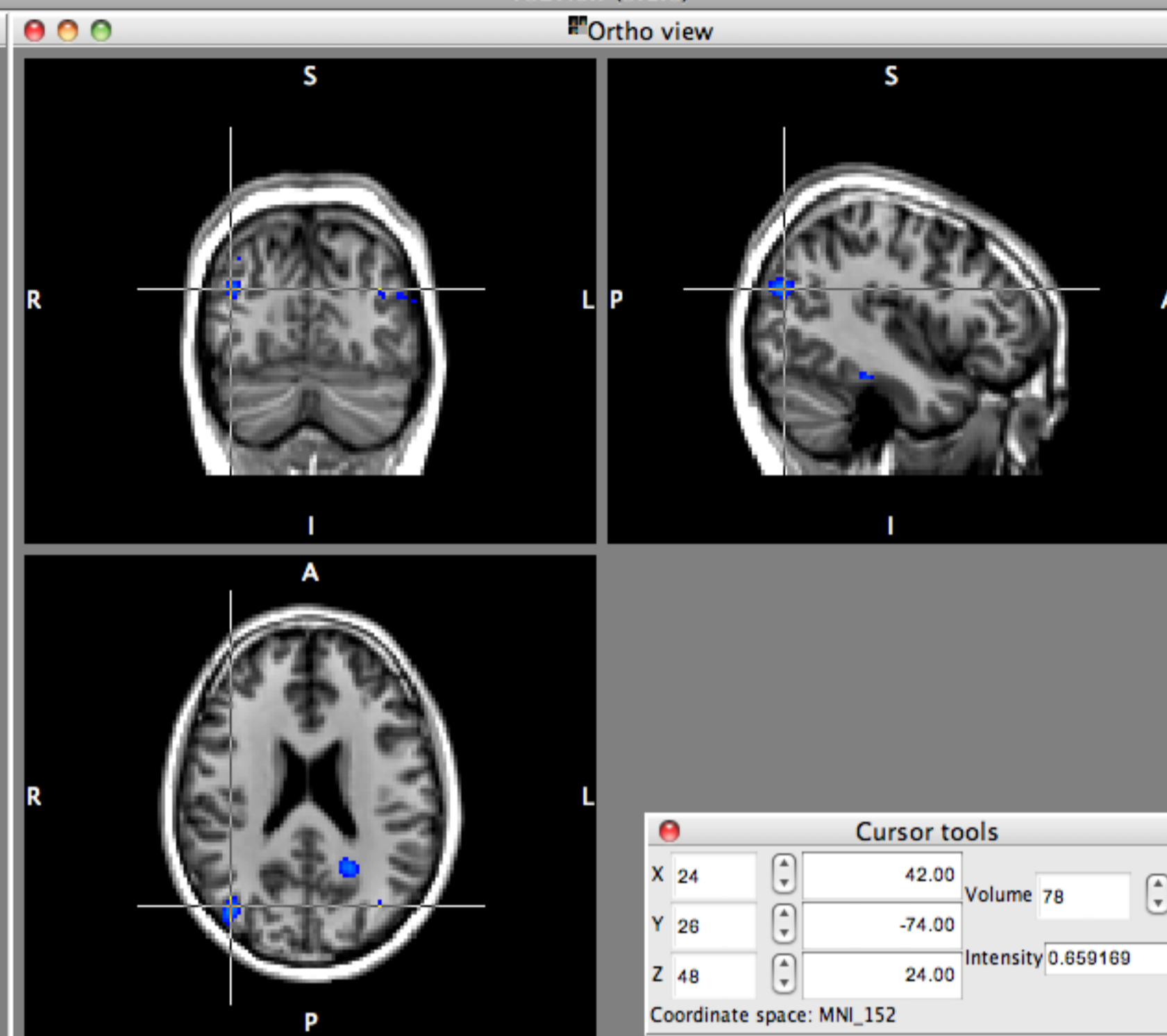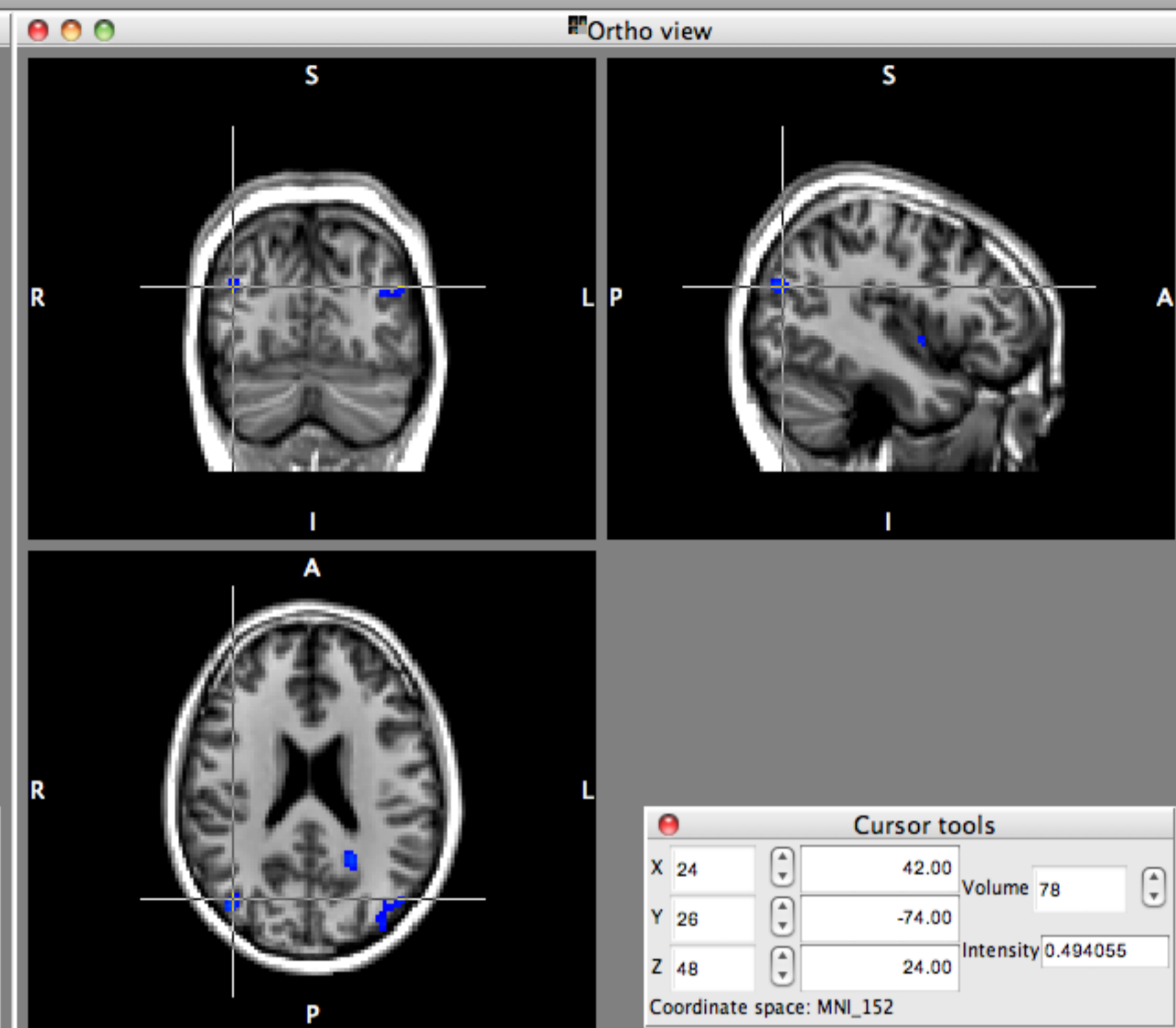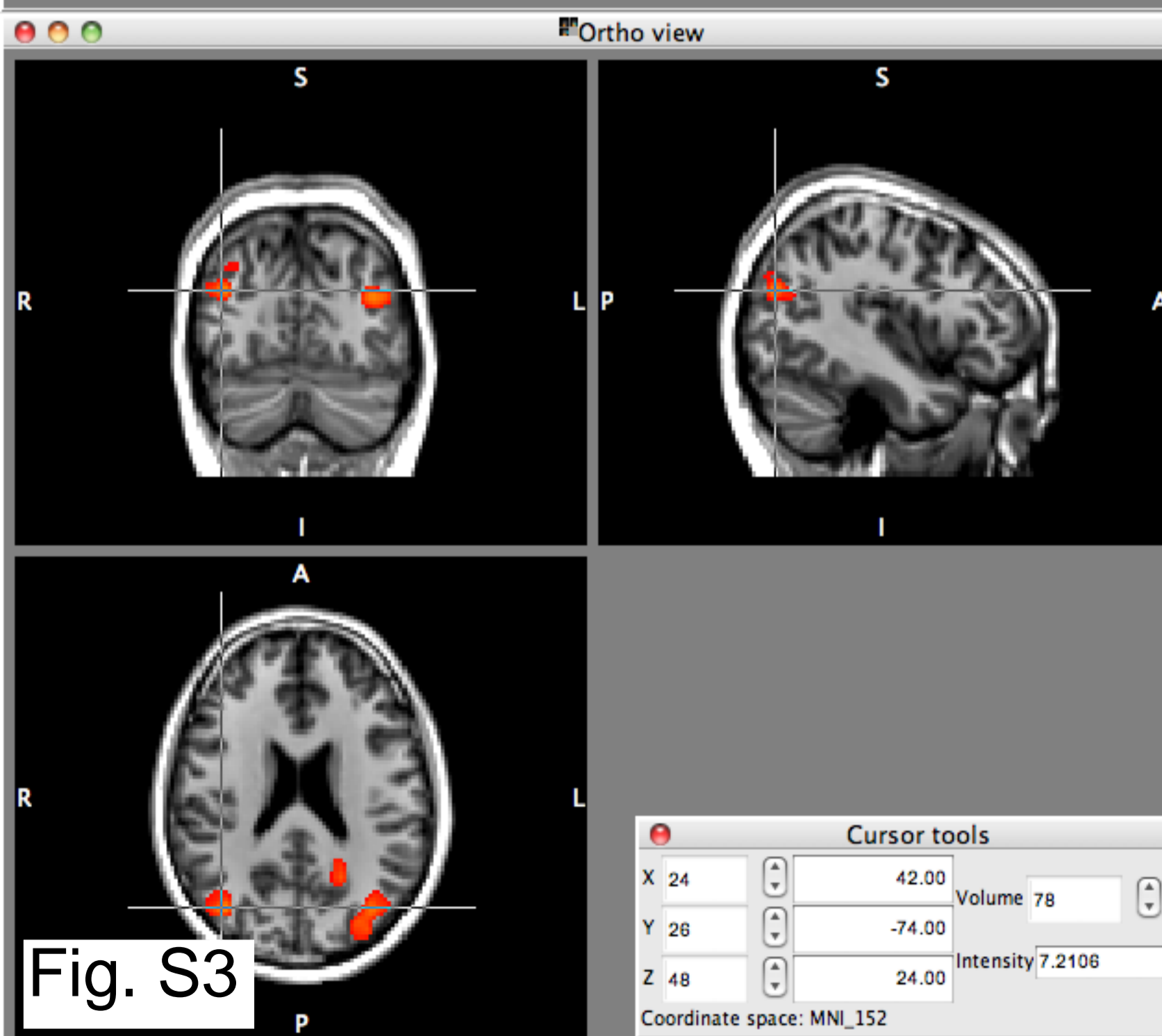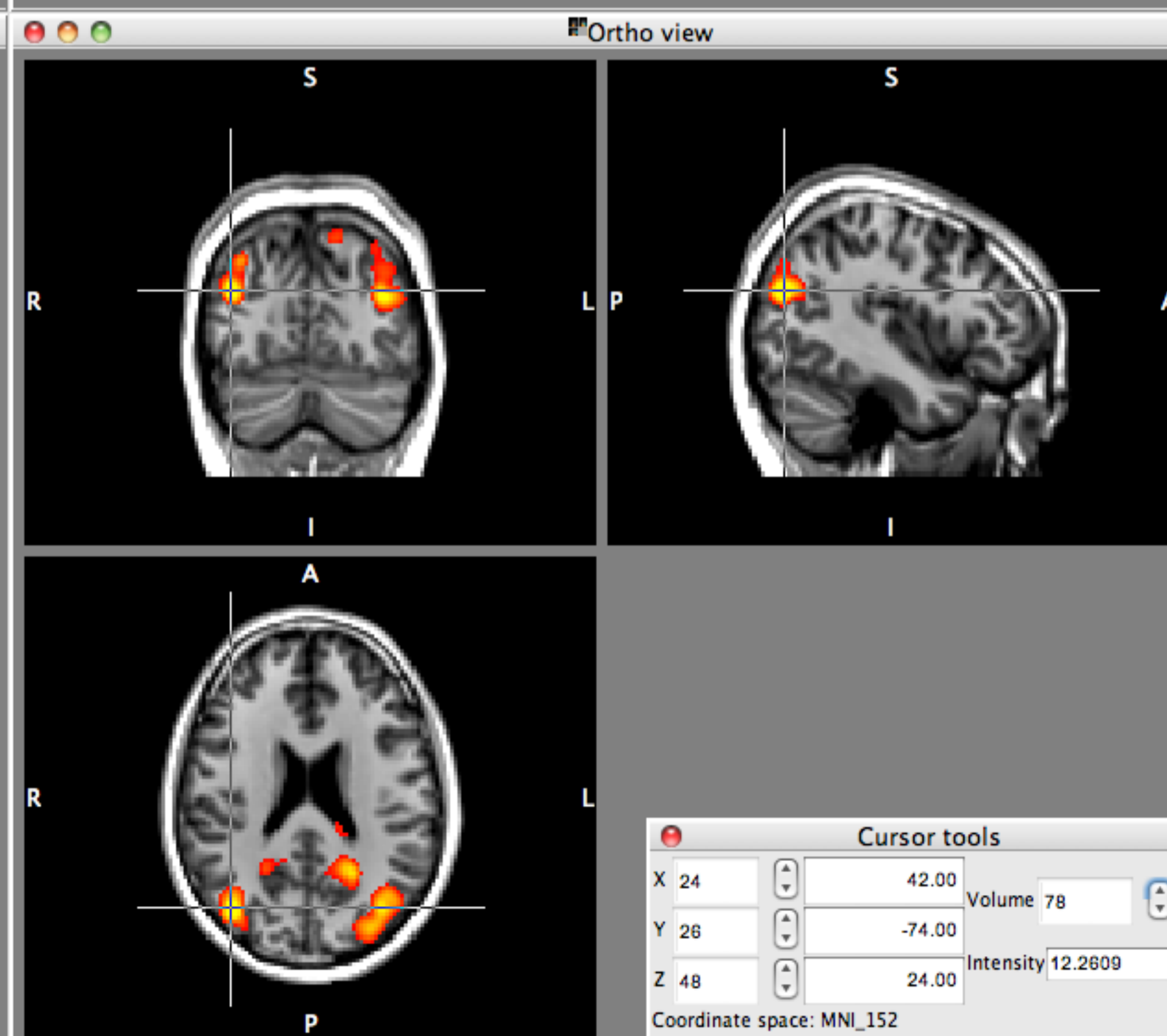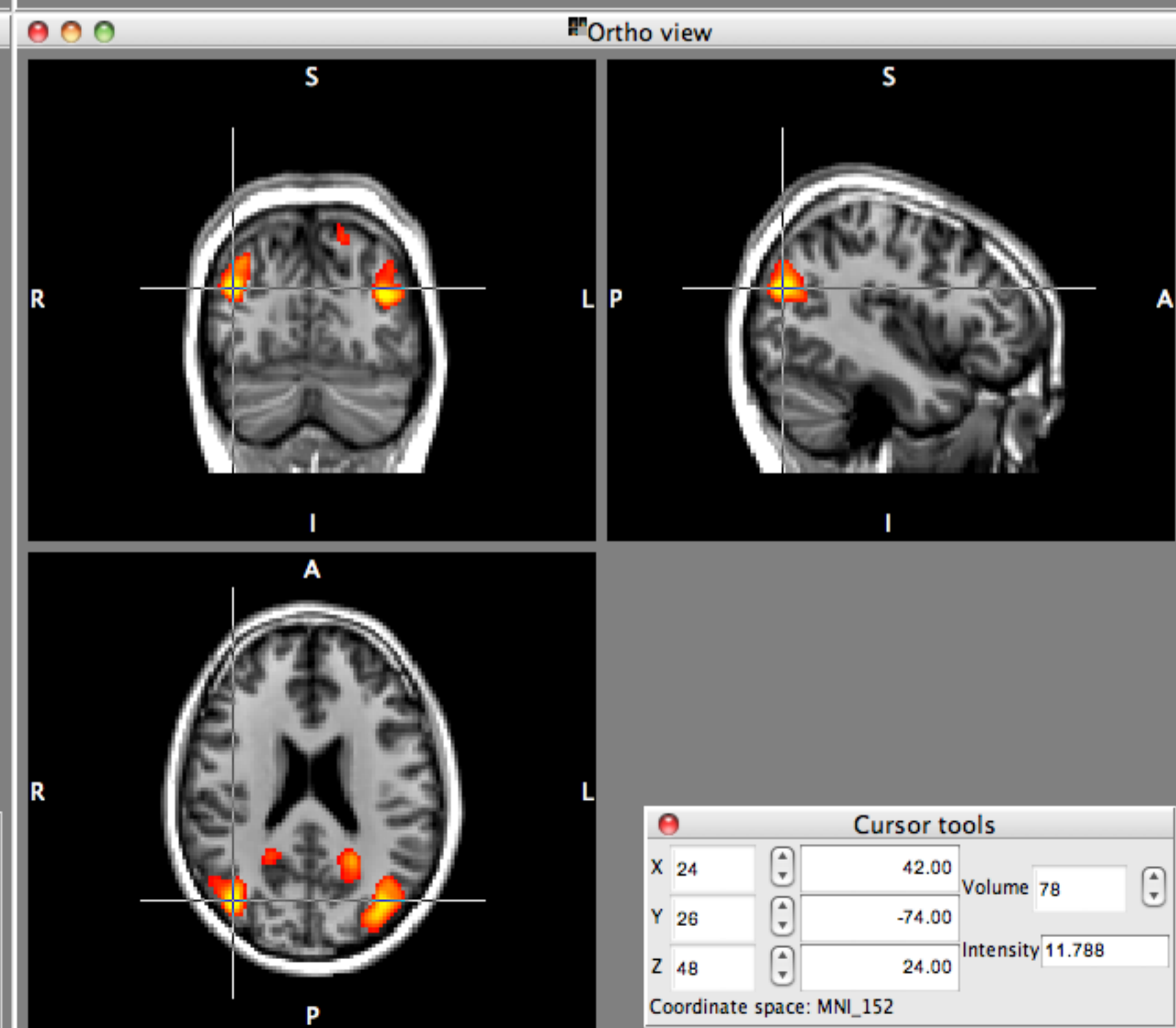

Fig. S3

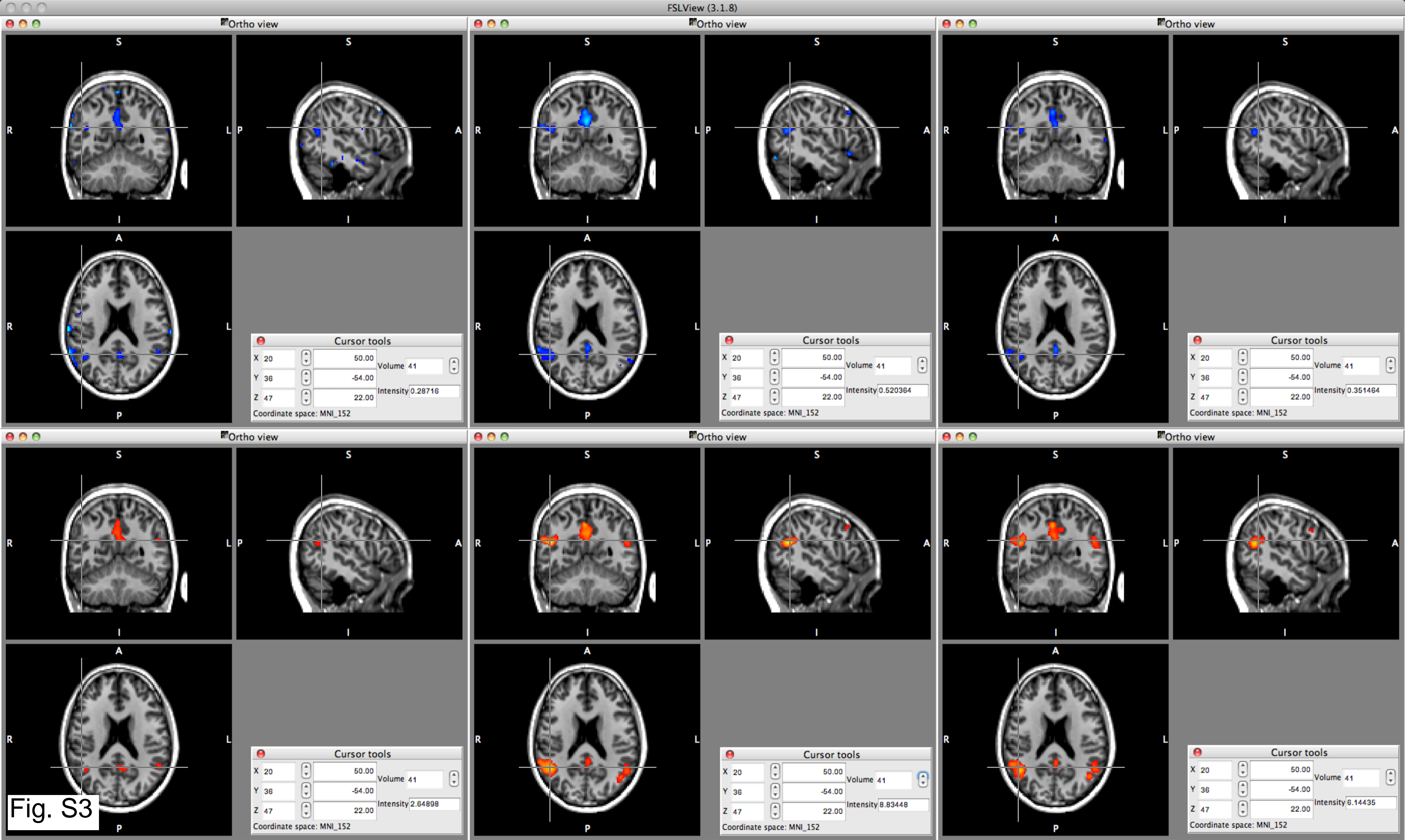

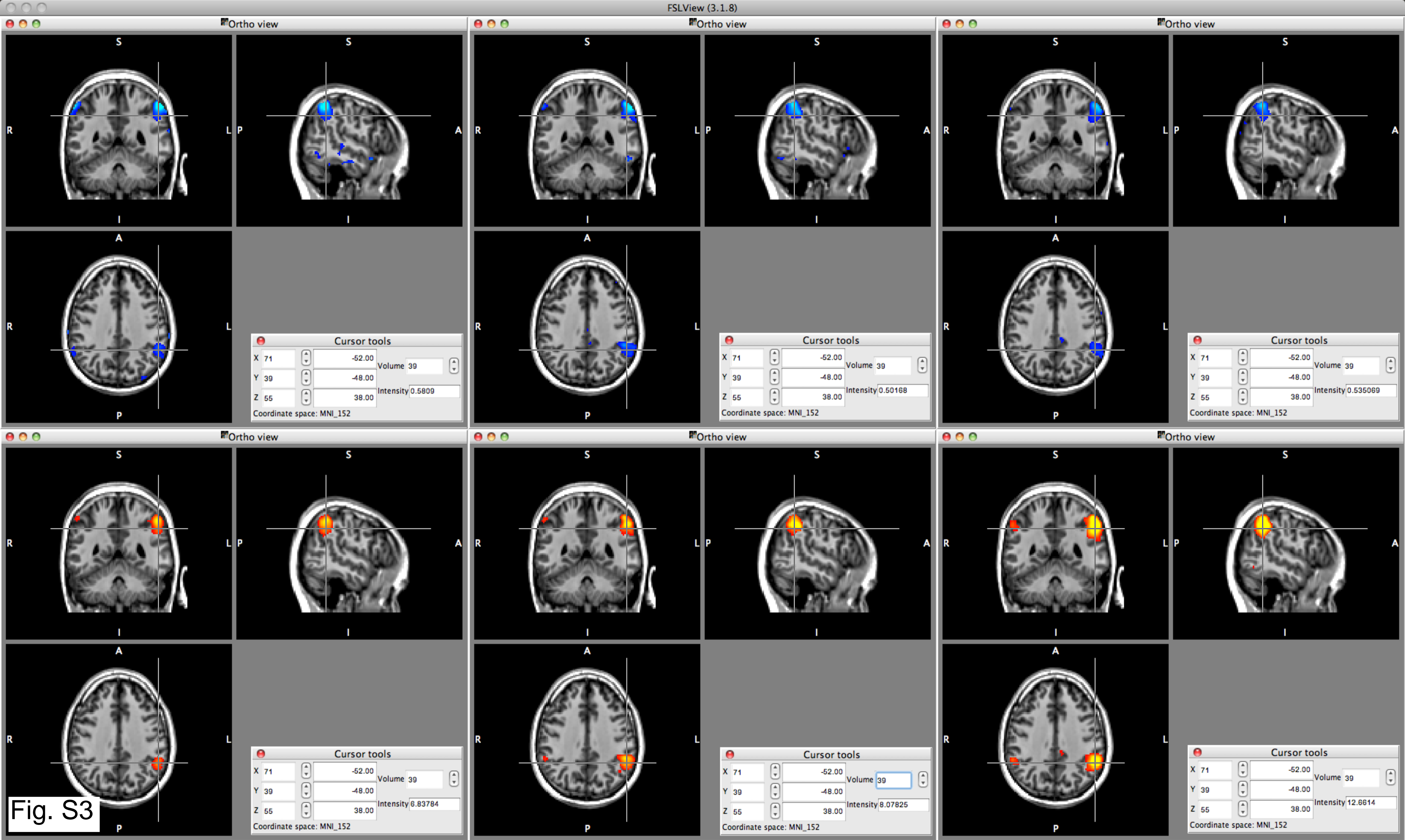

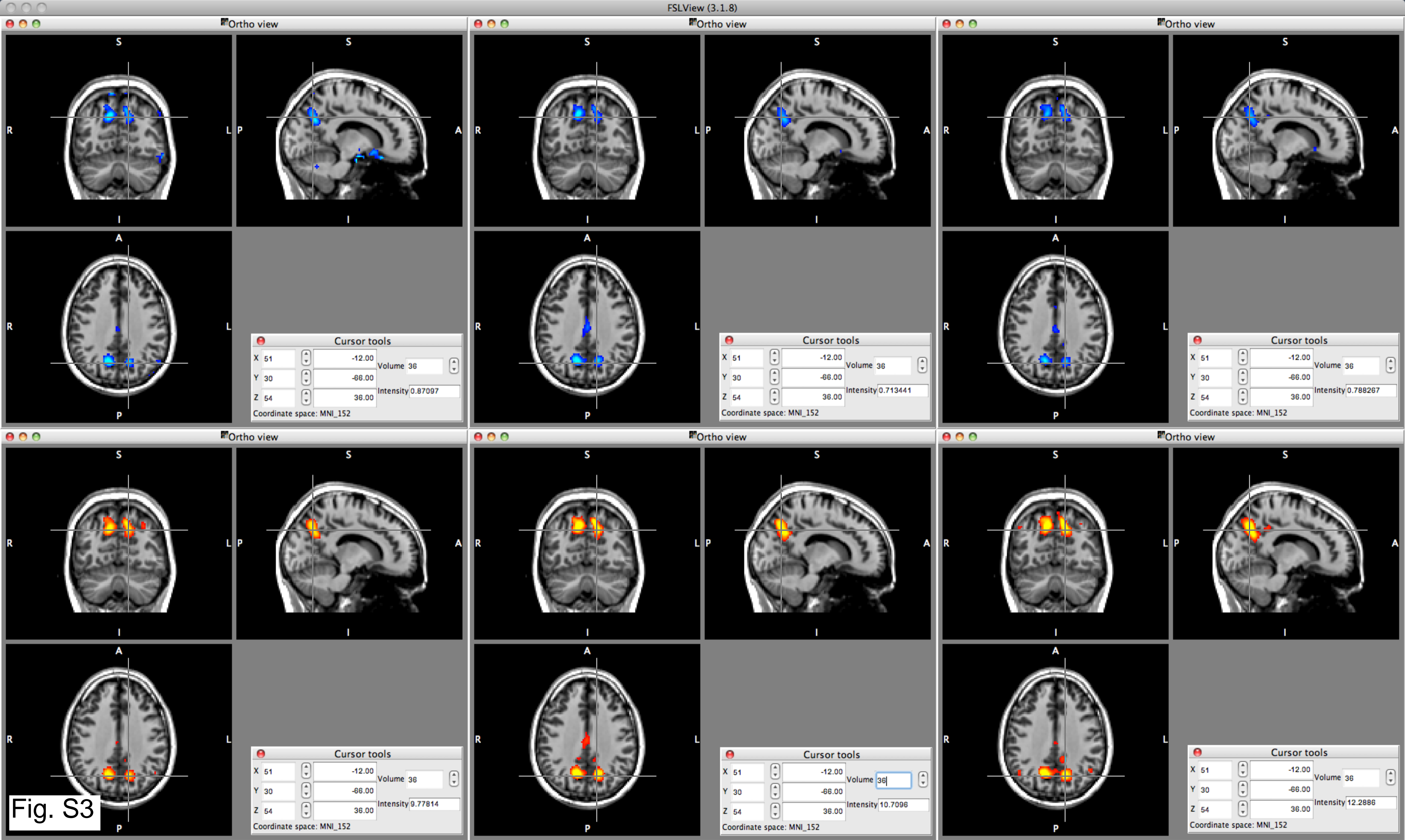

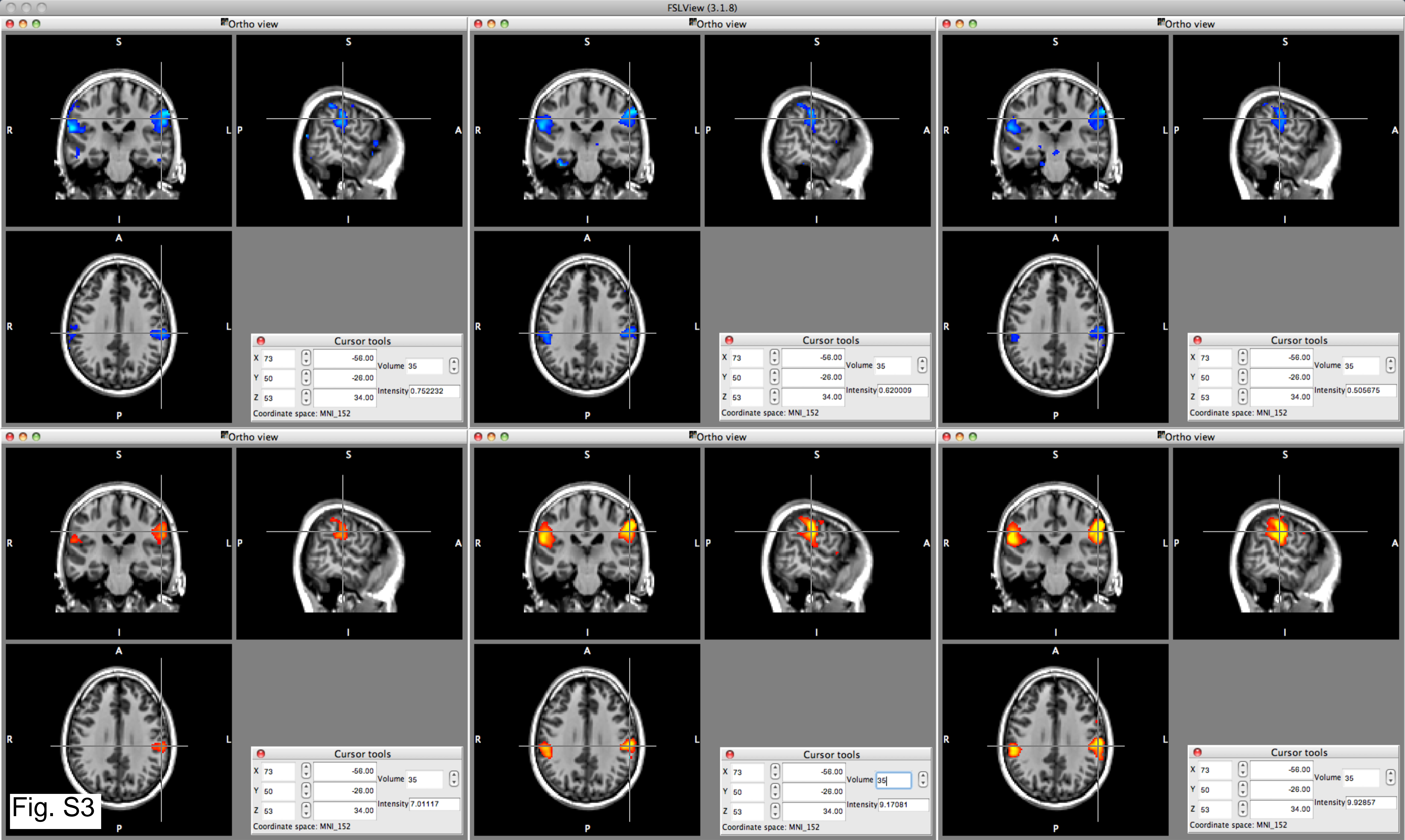

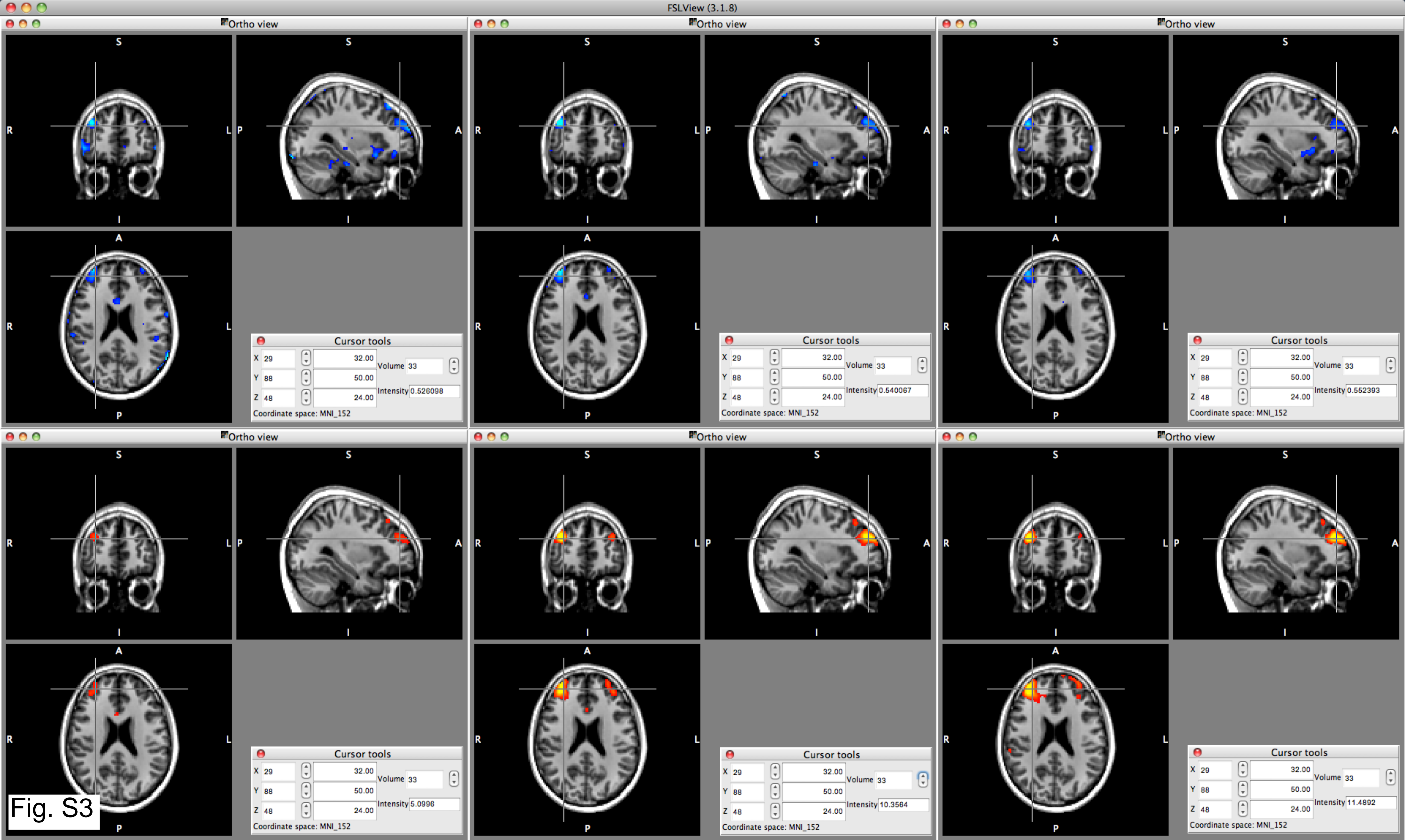

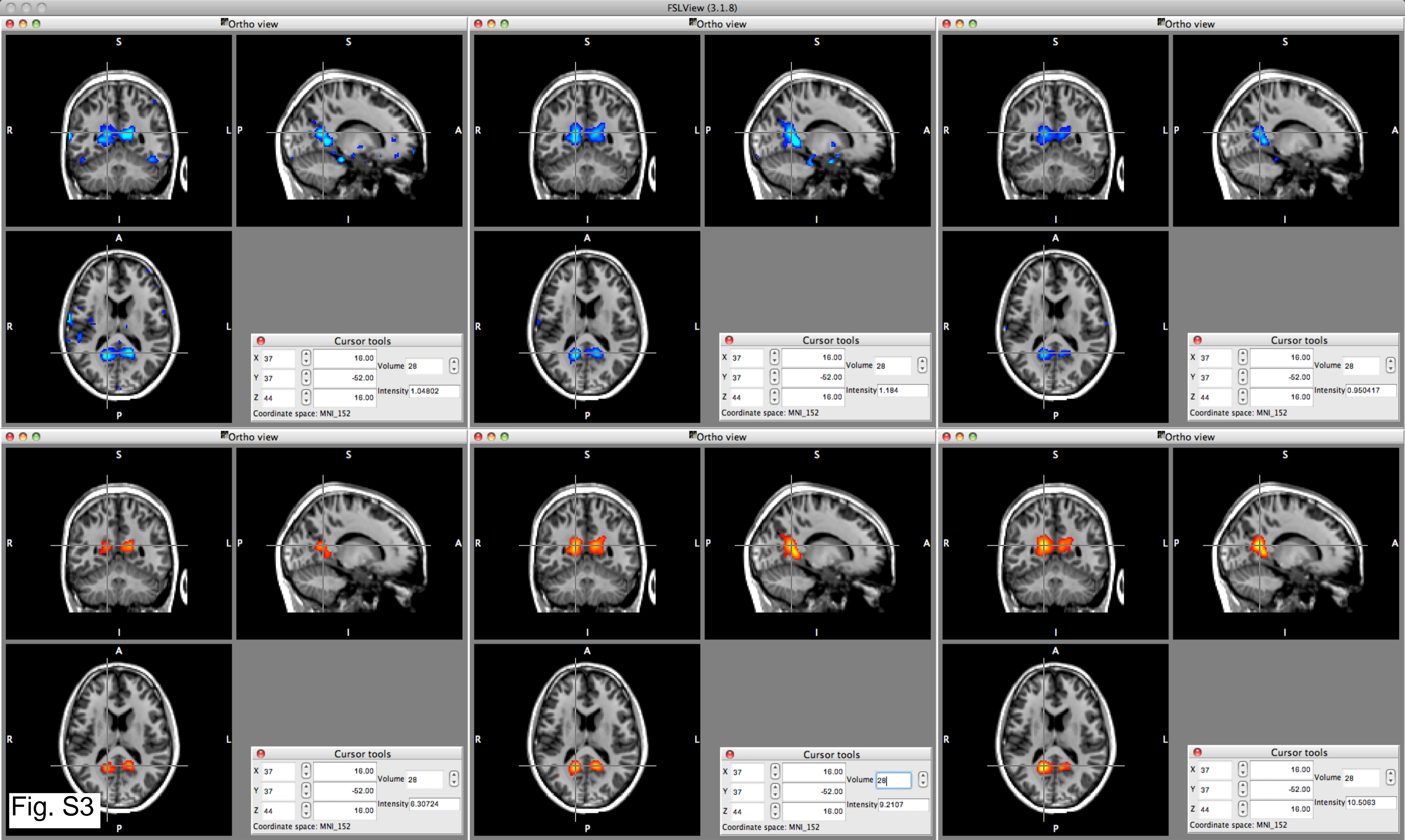

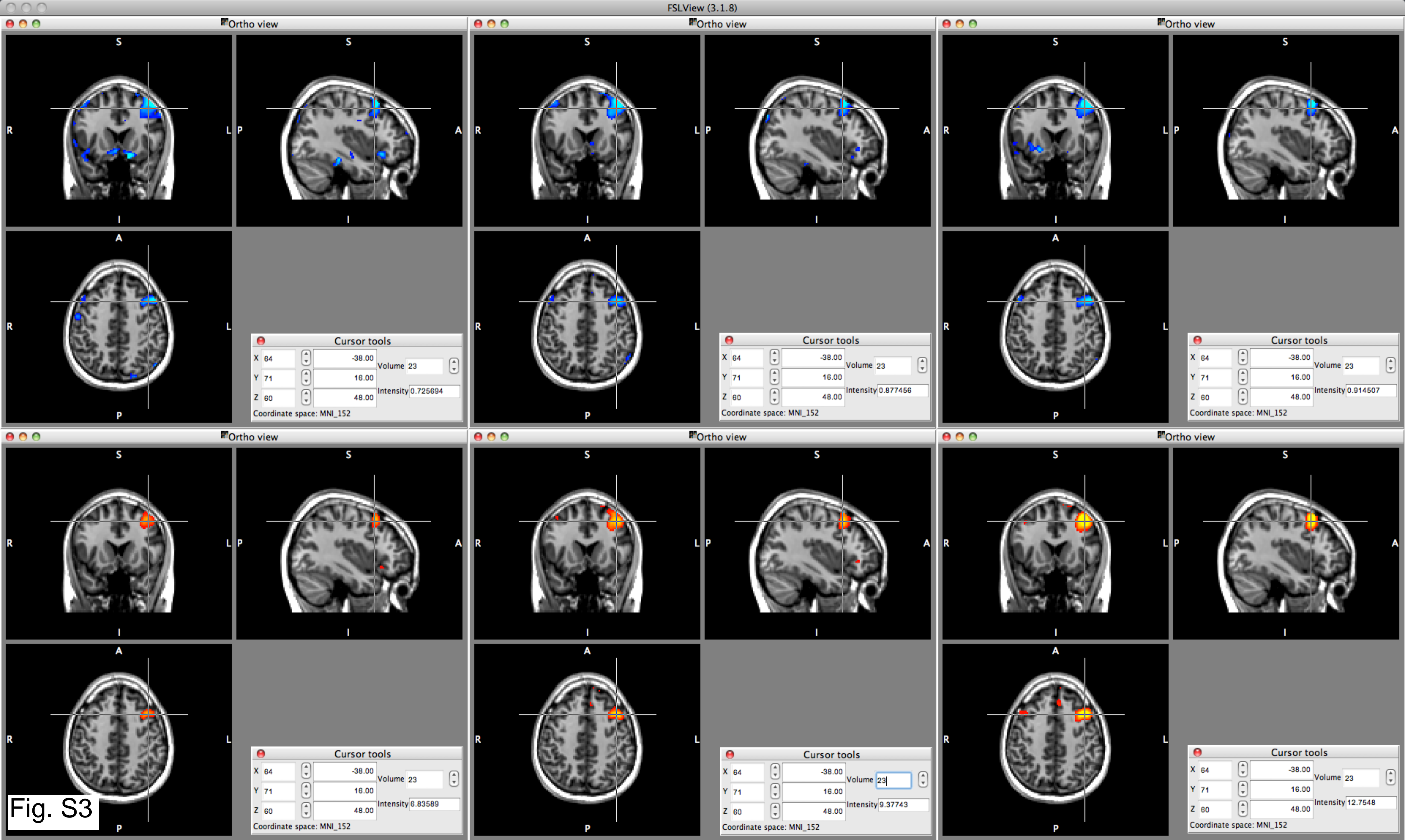

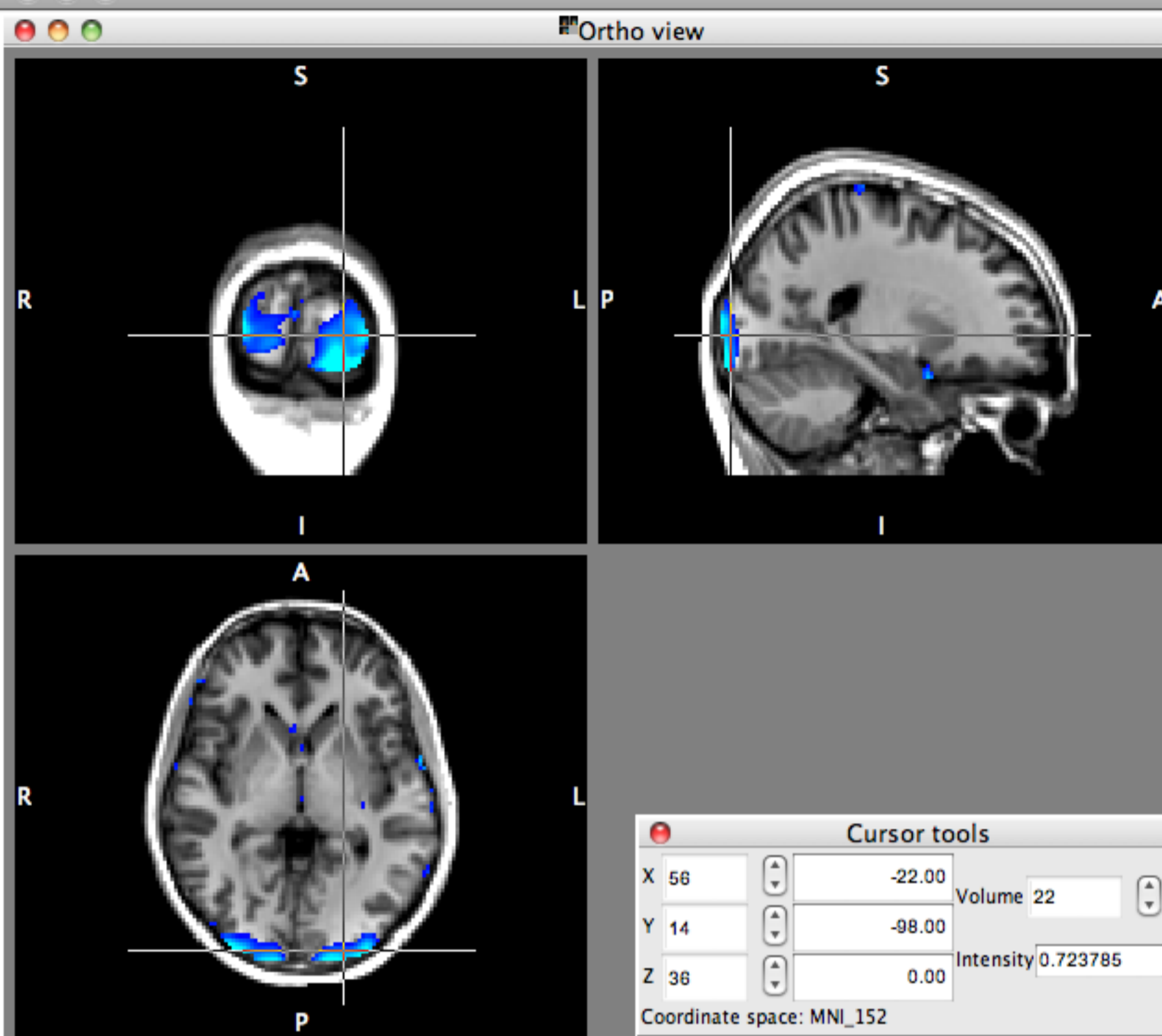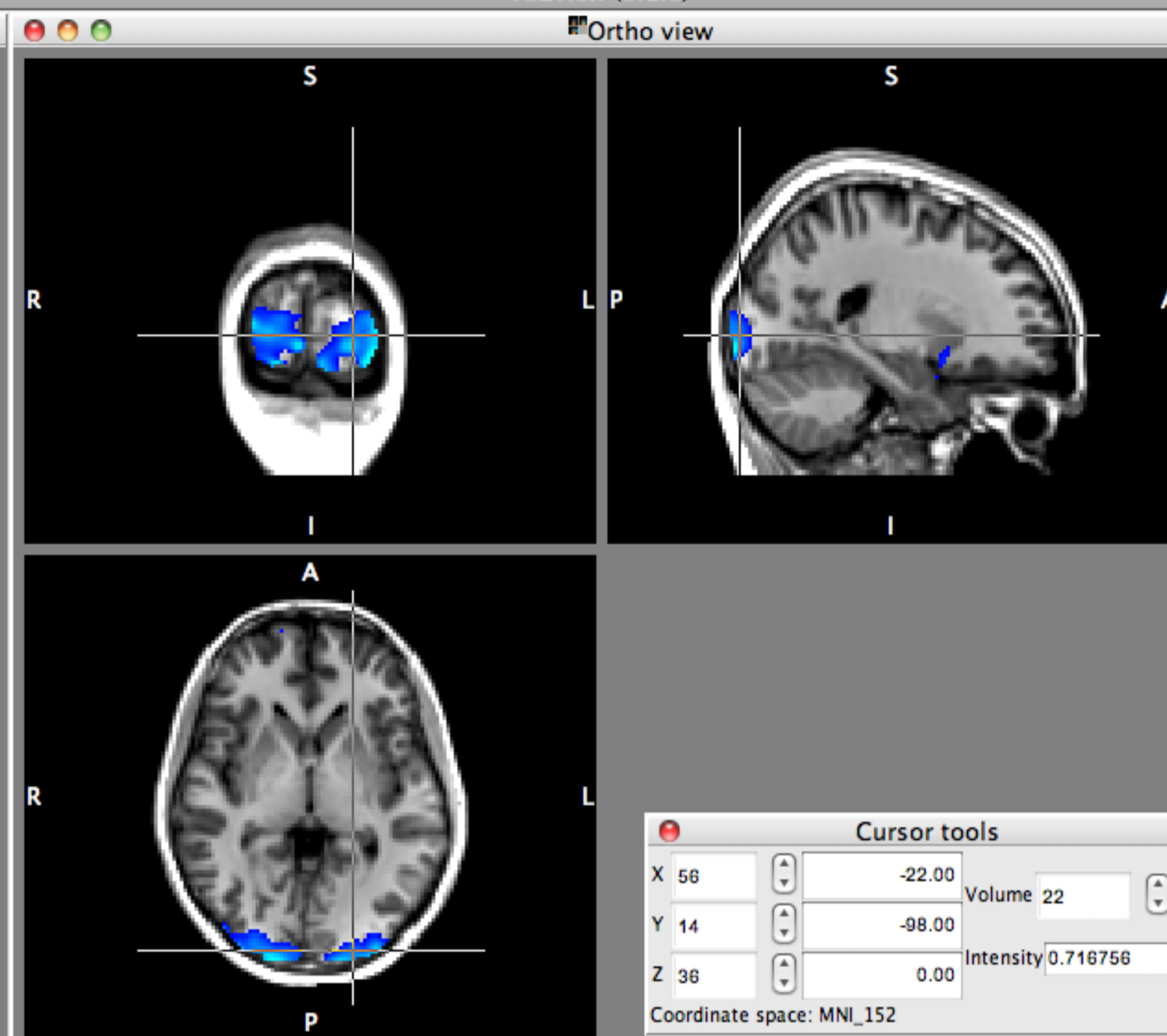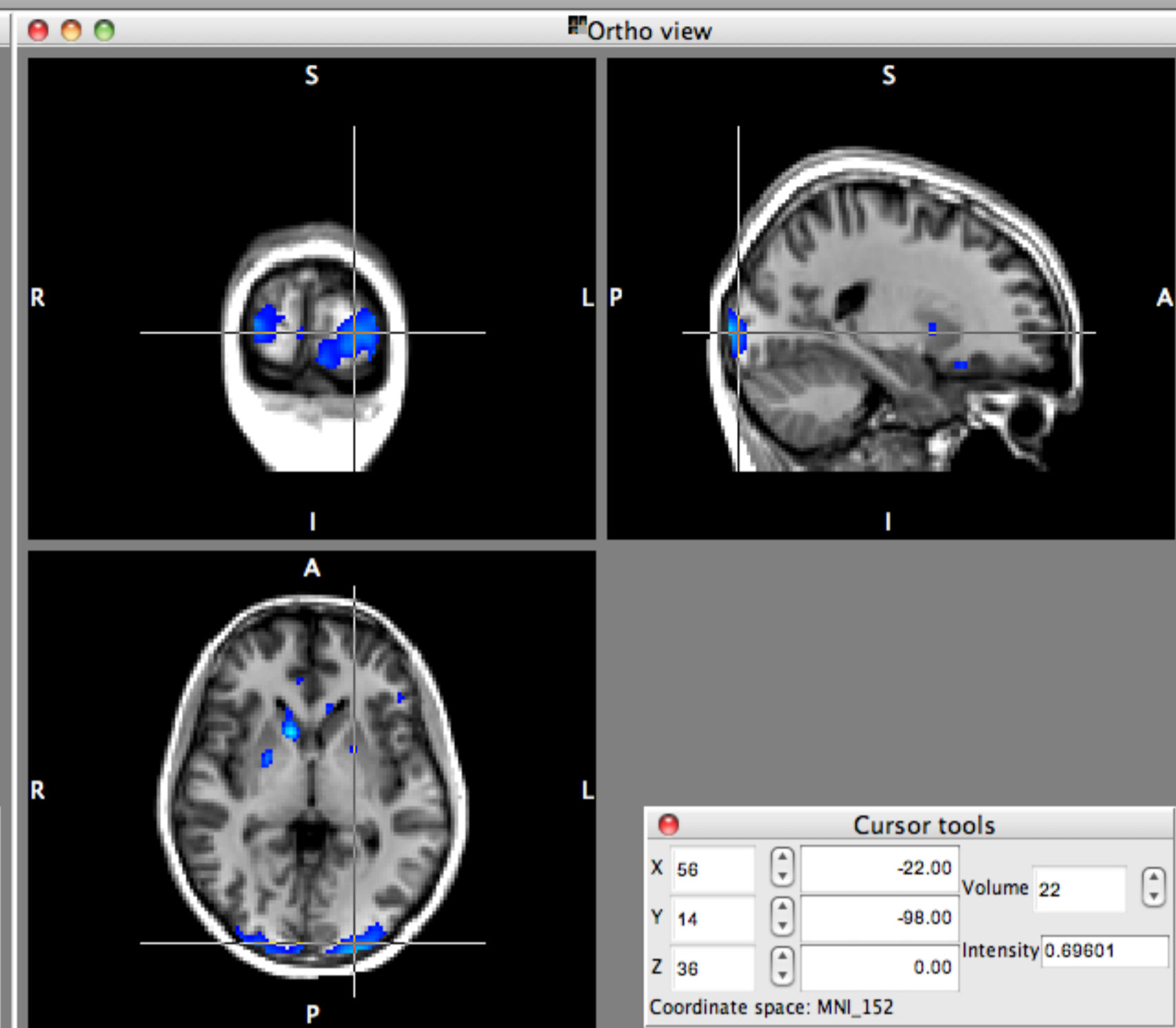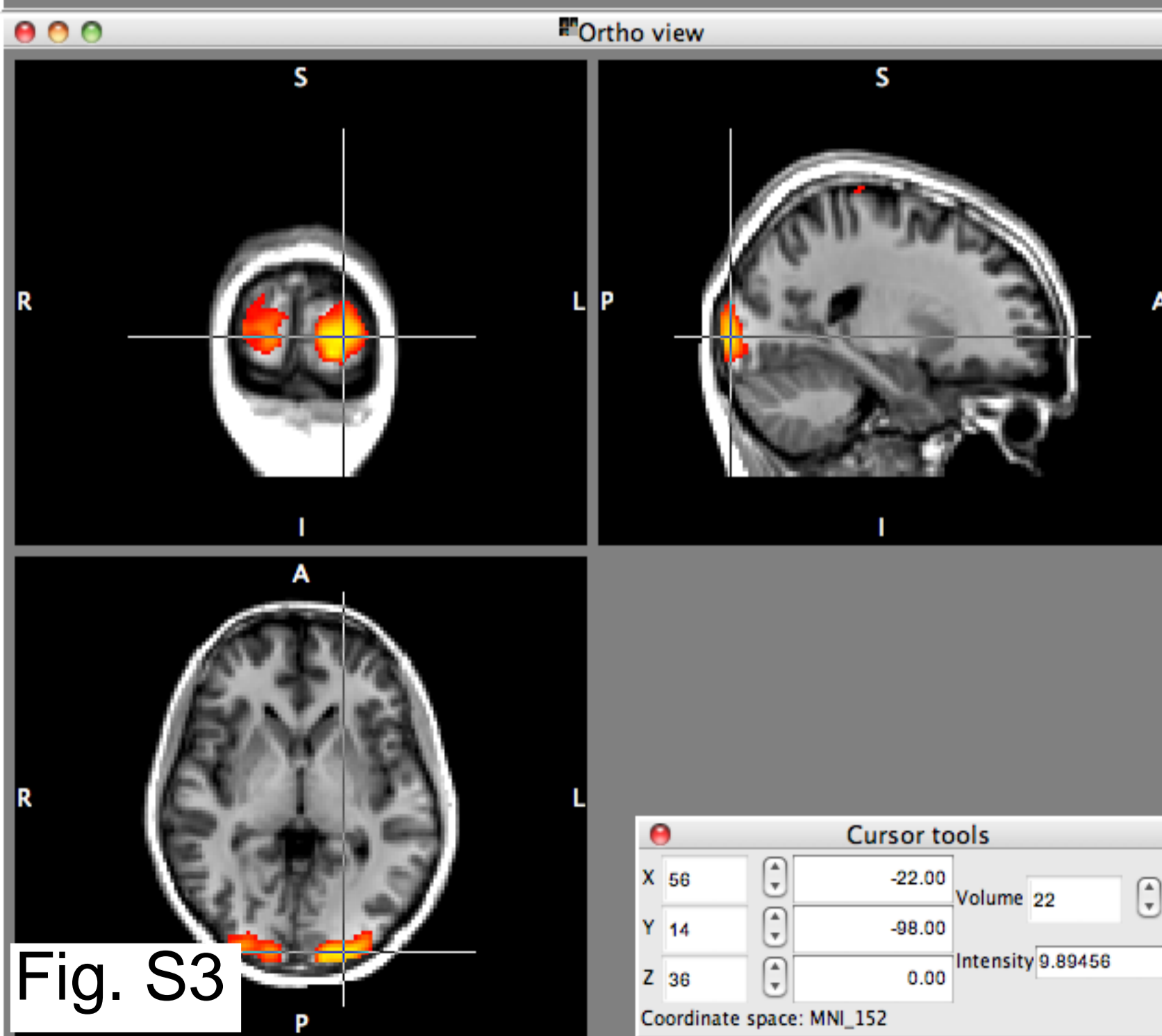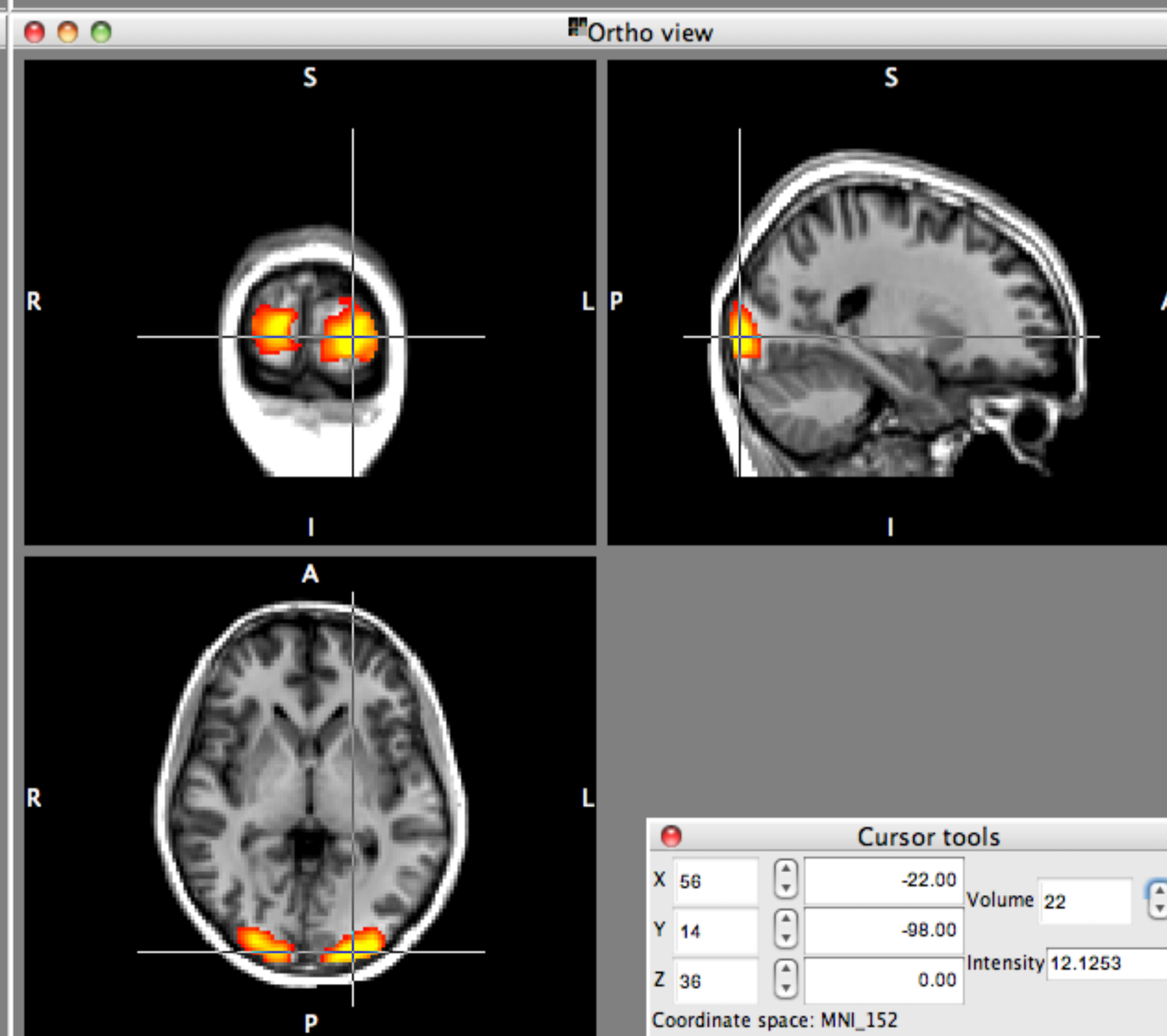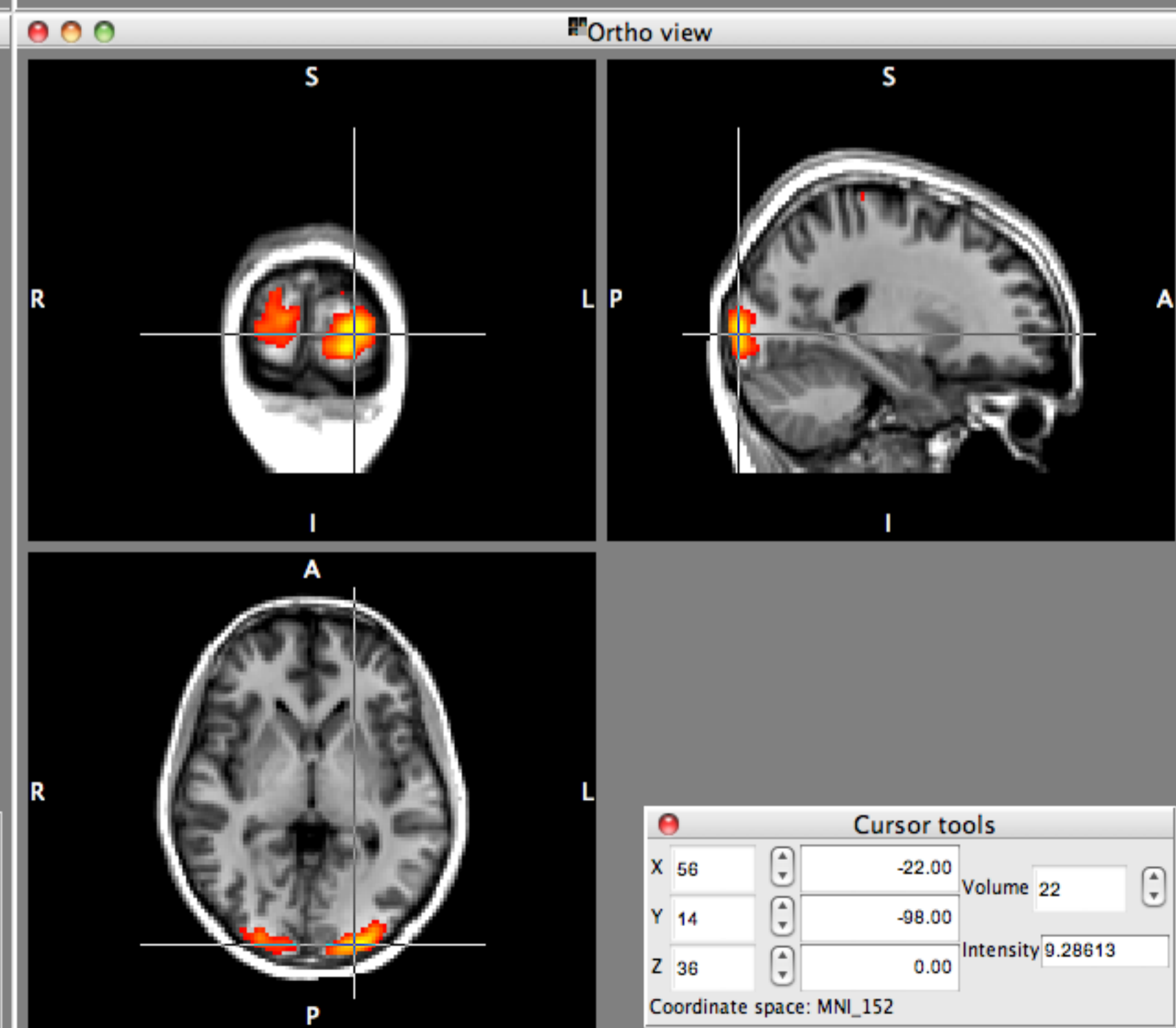

Fig. S3

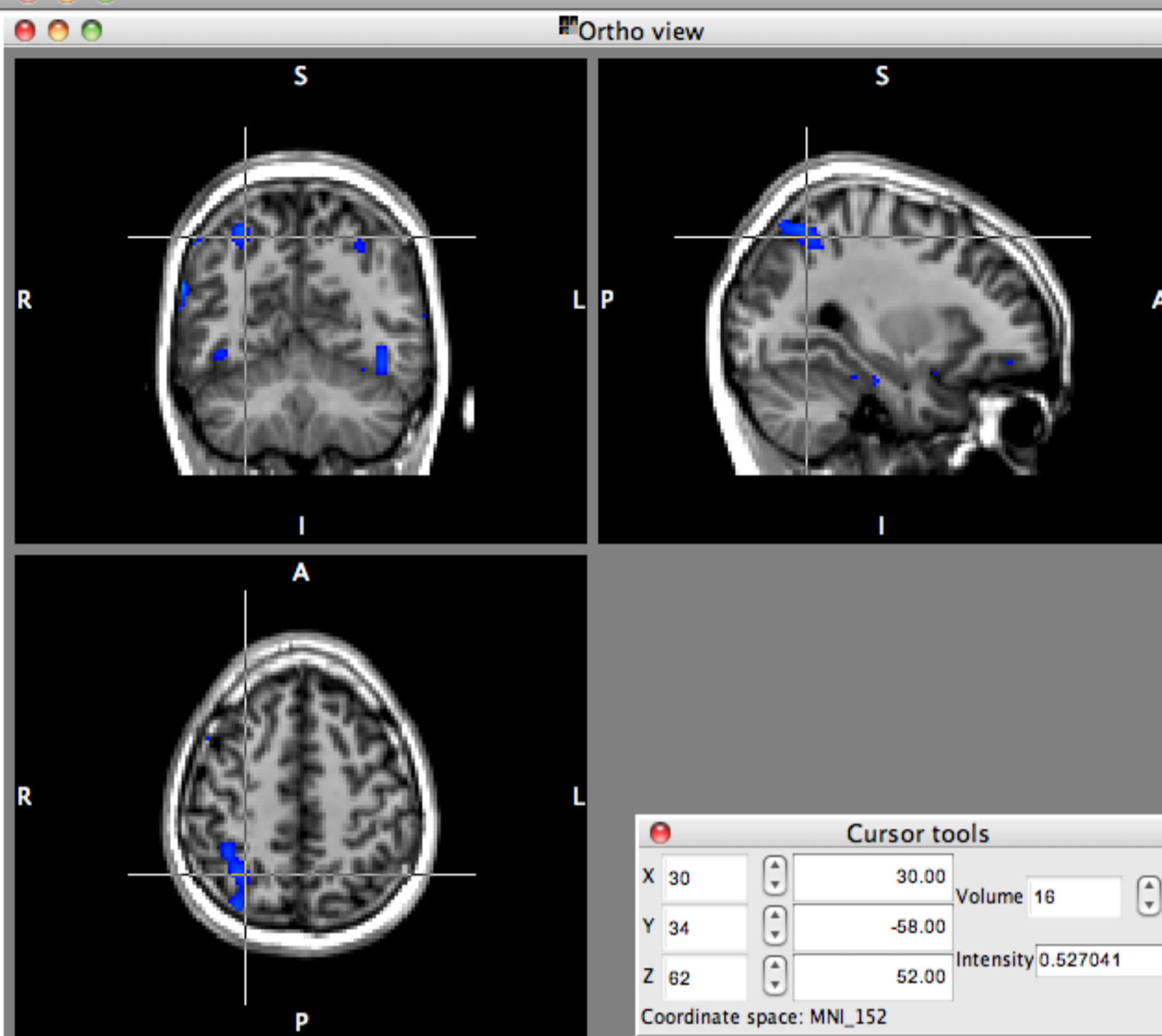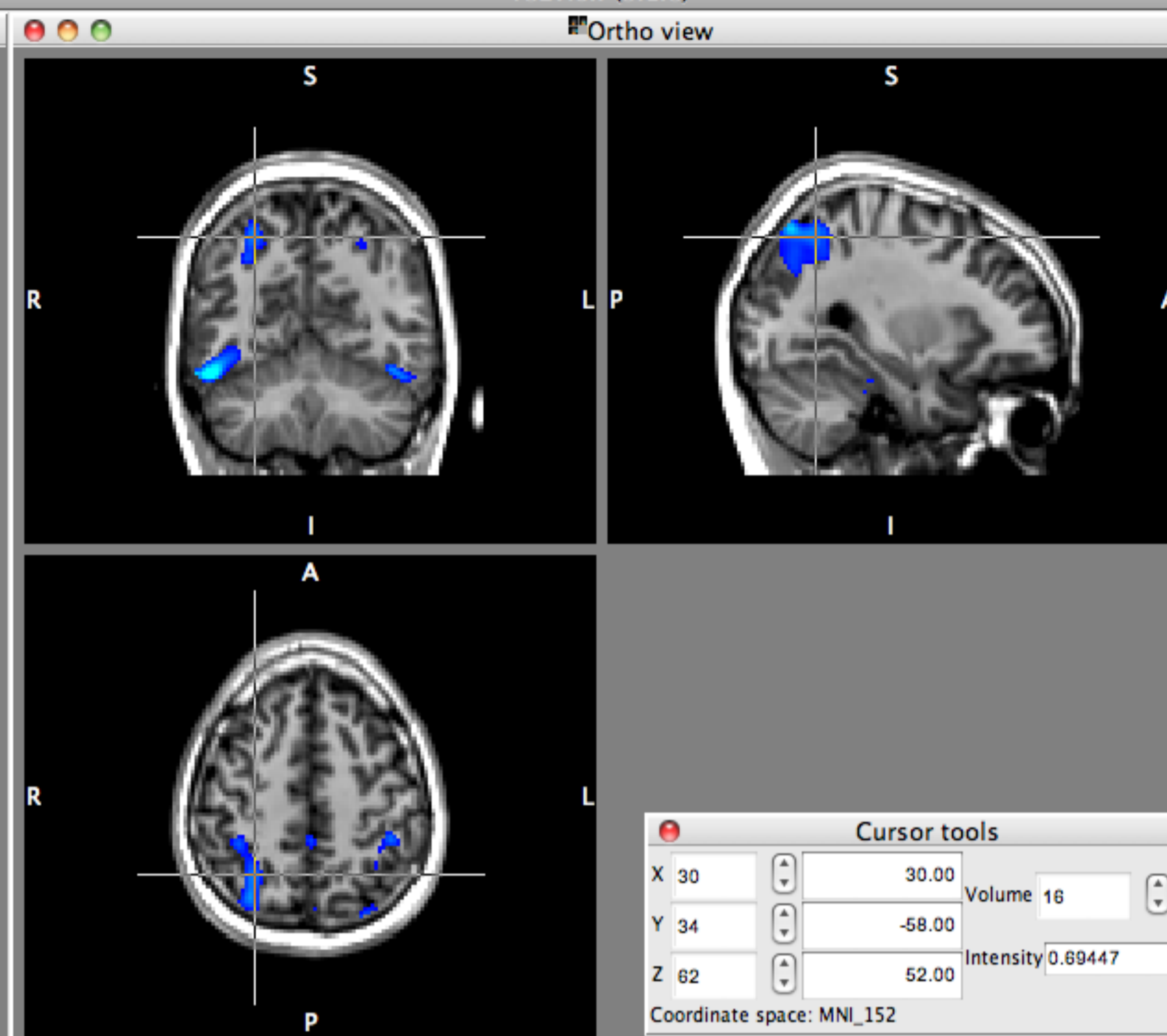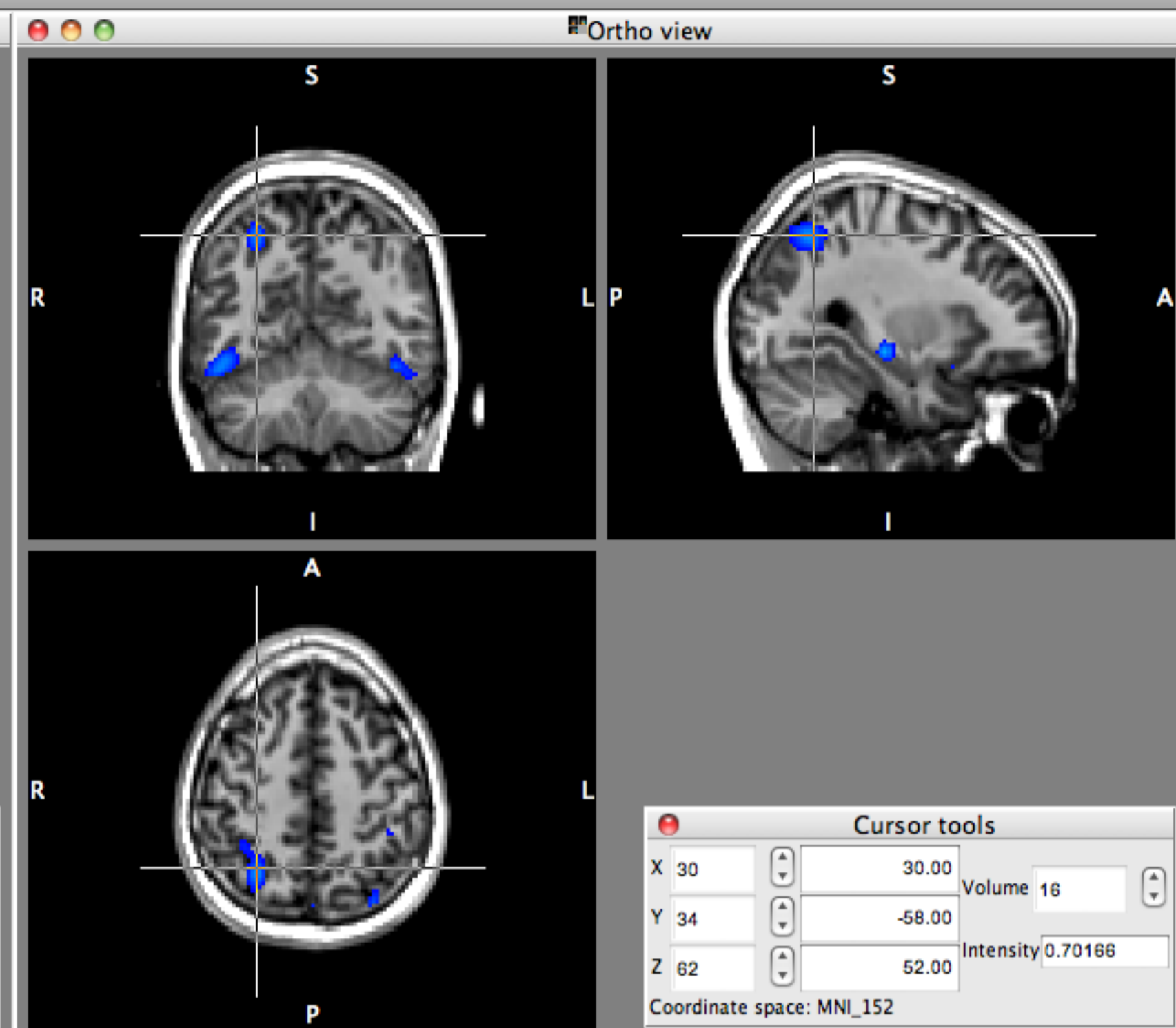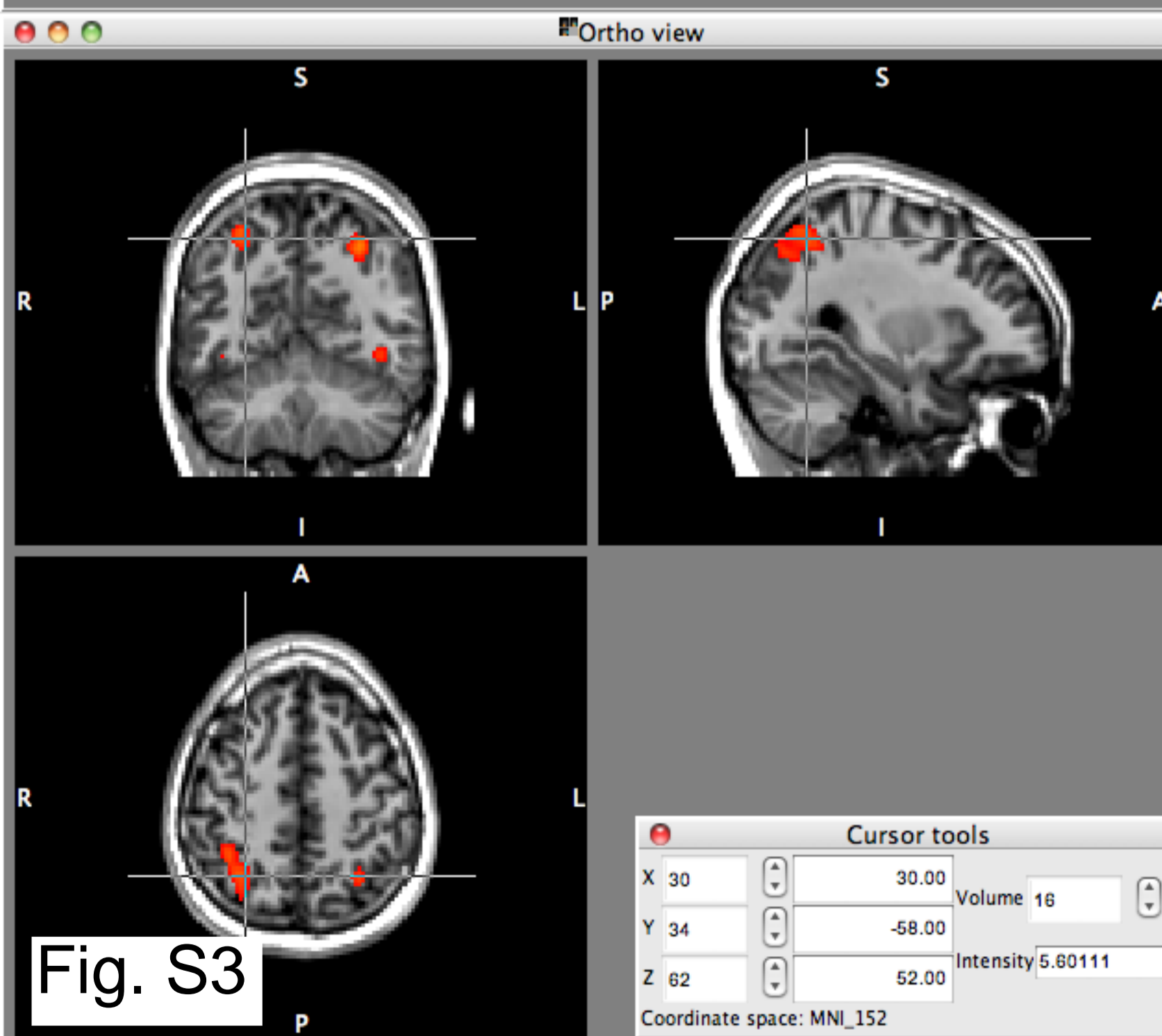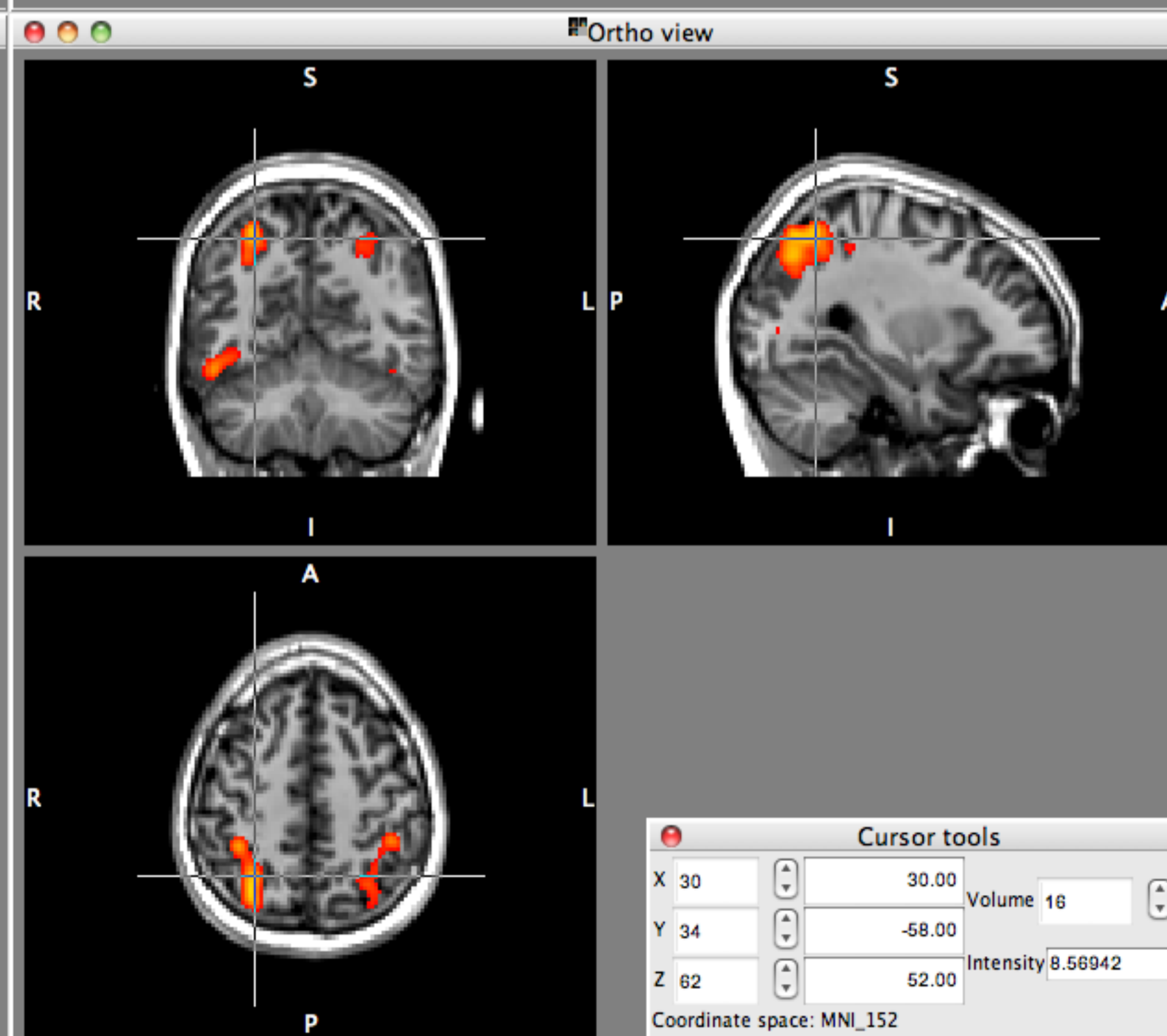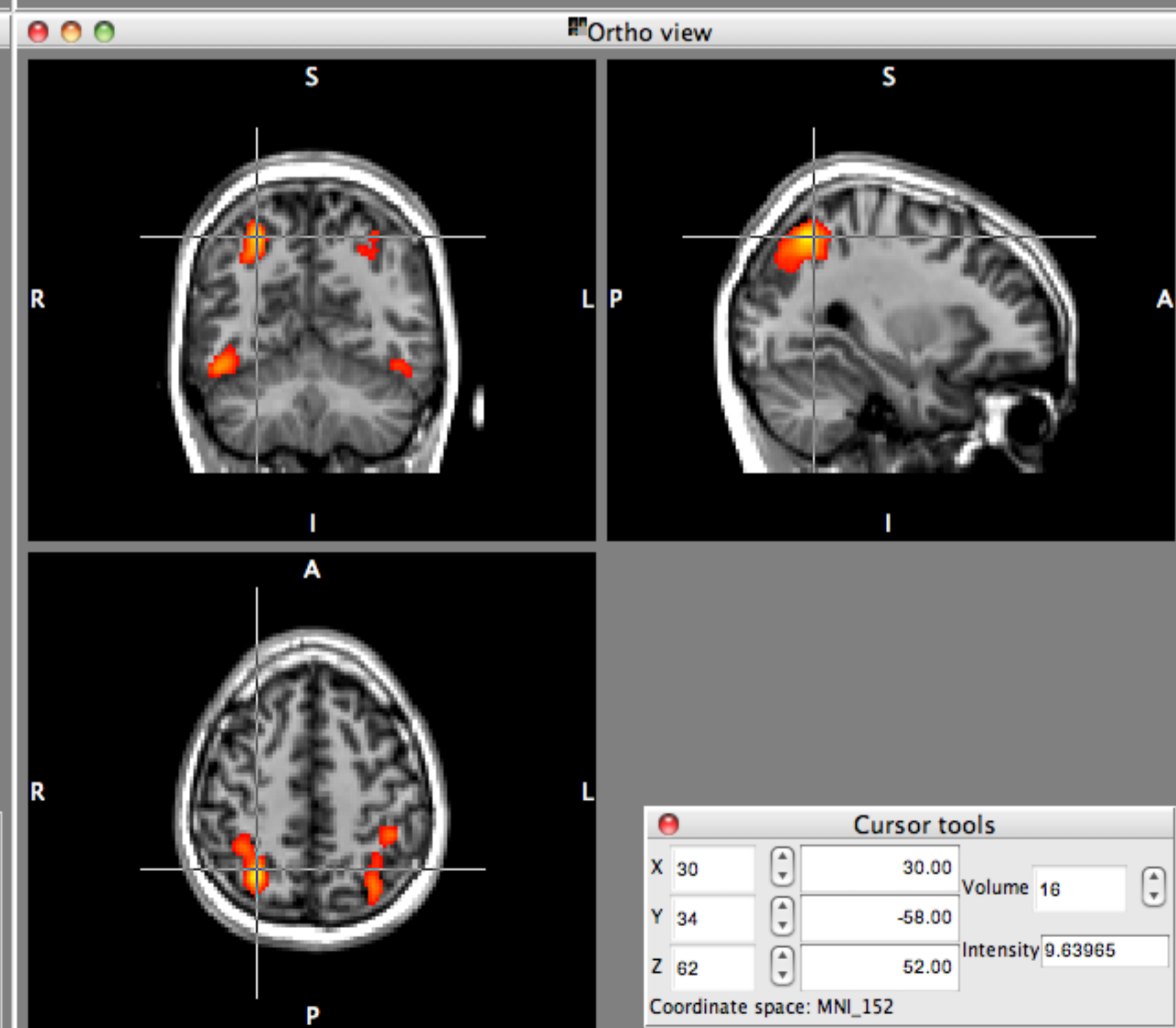

Fig. S3

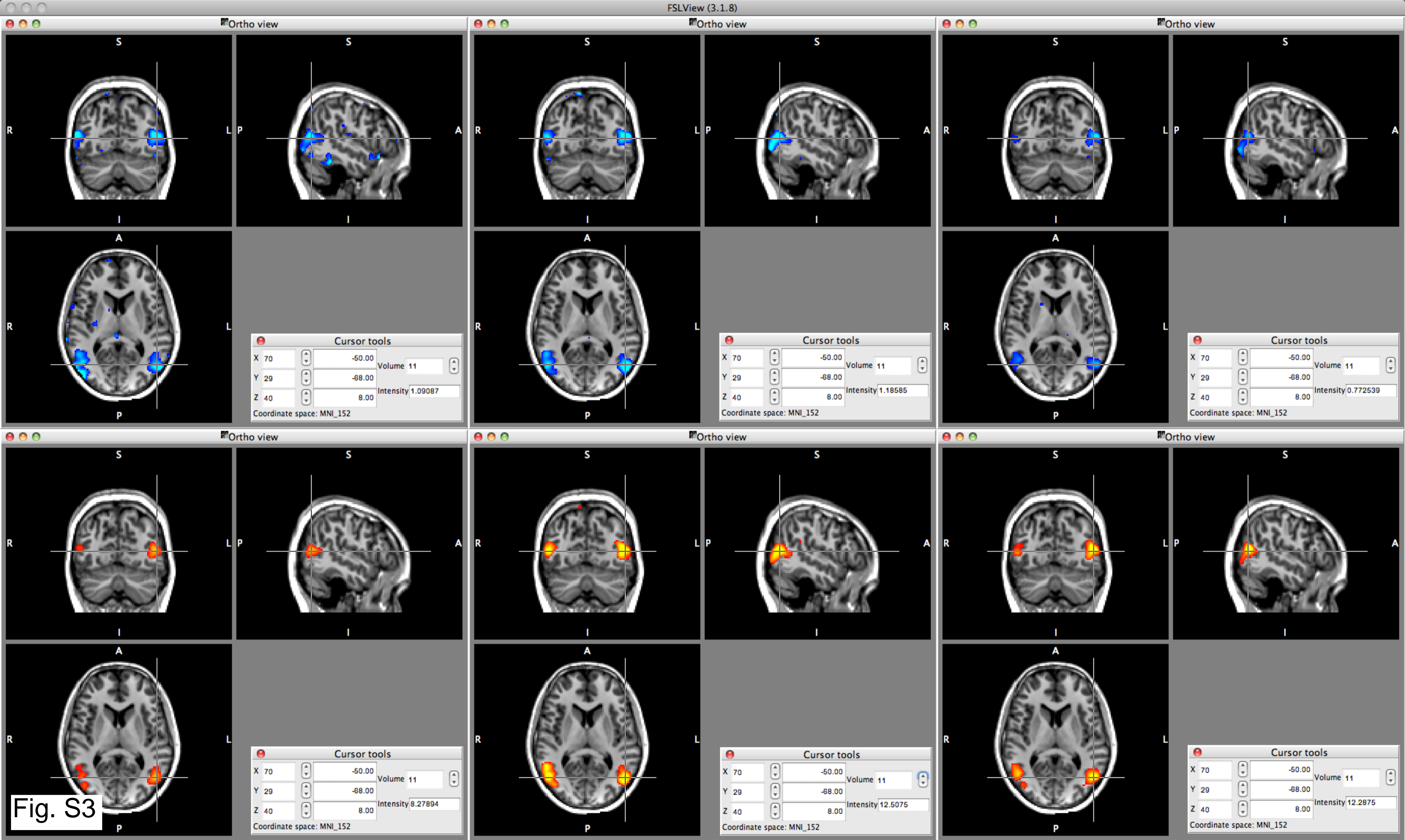

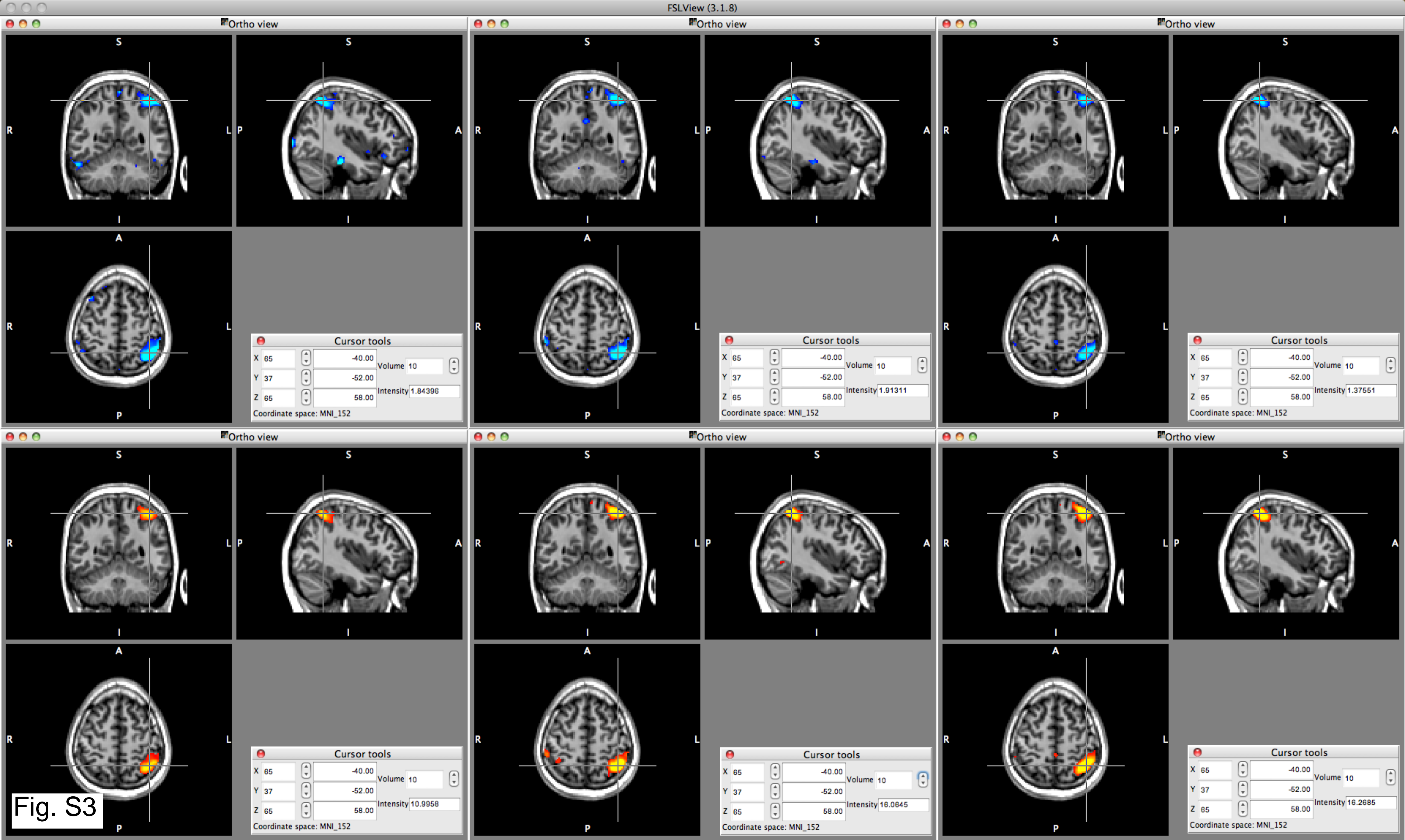

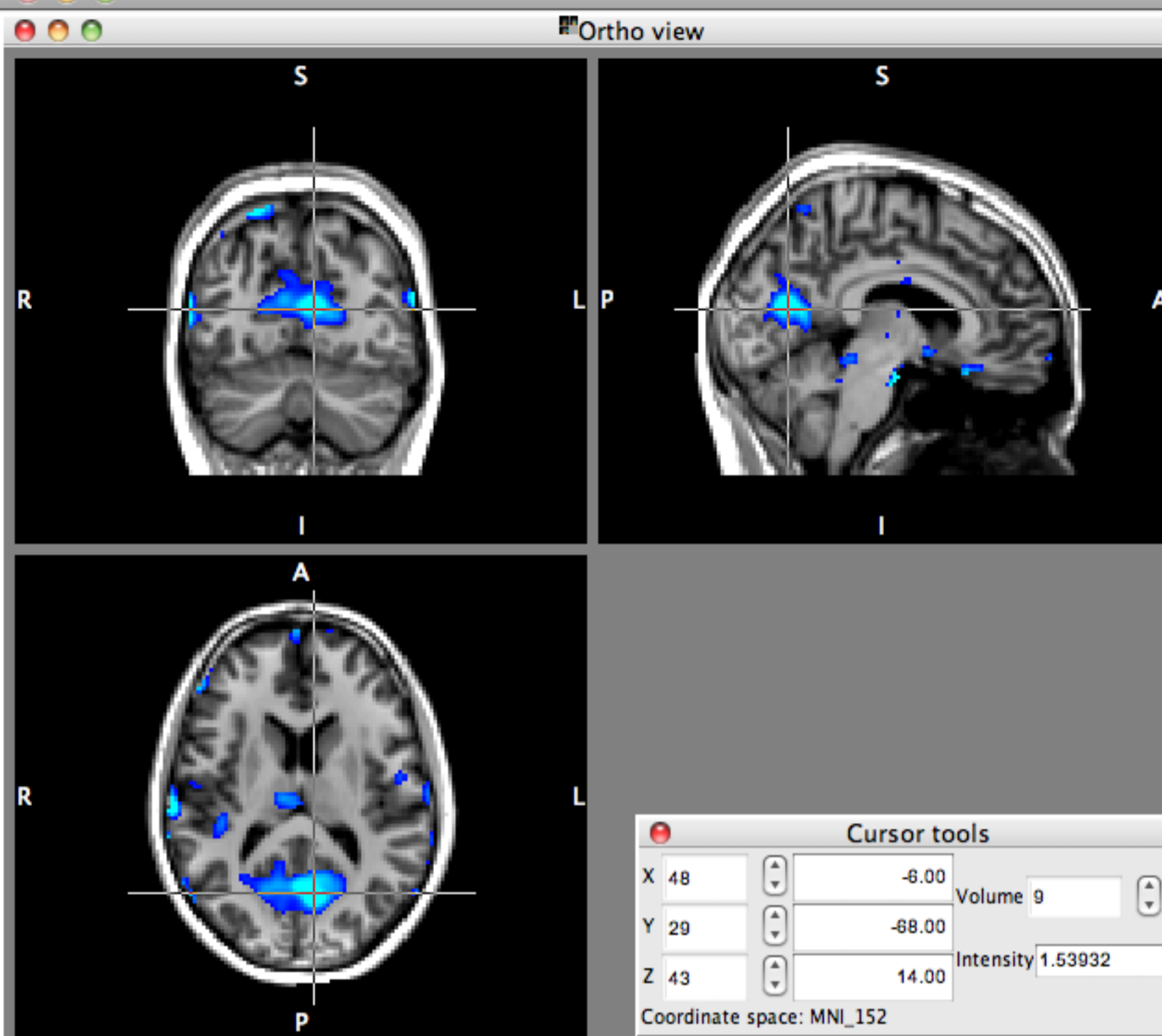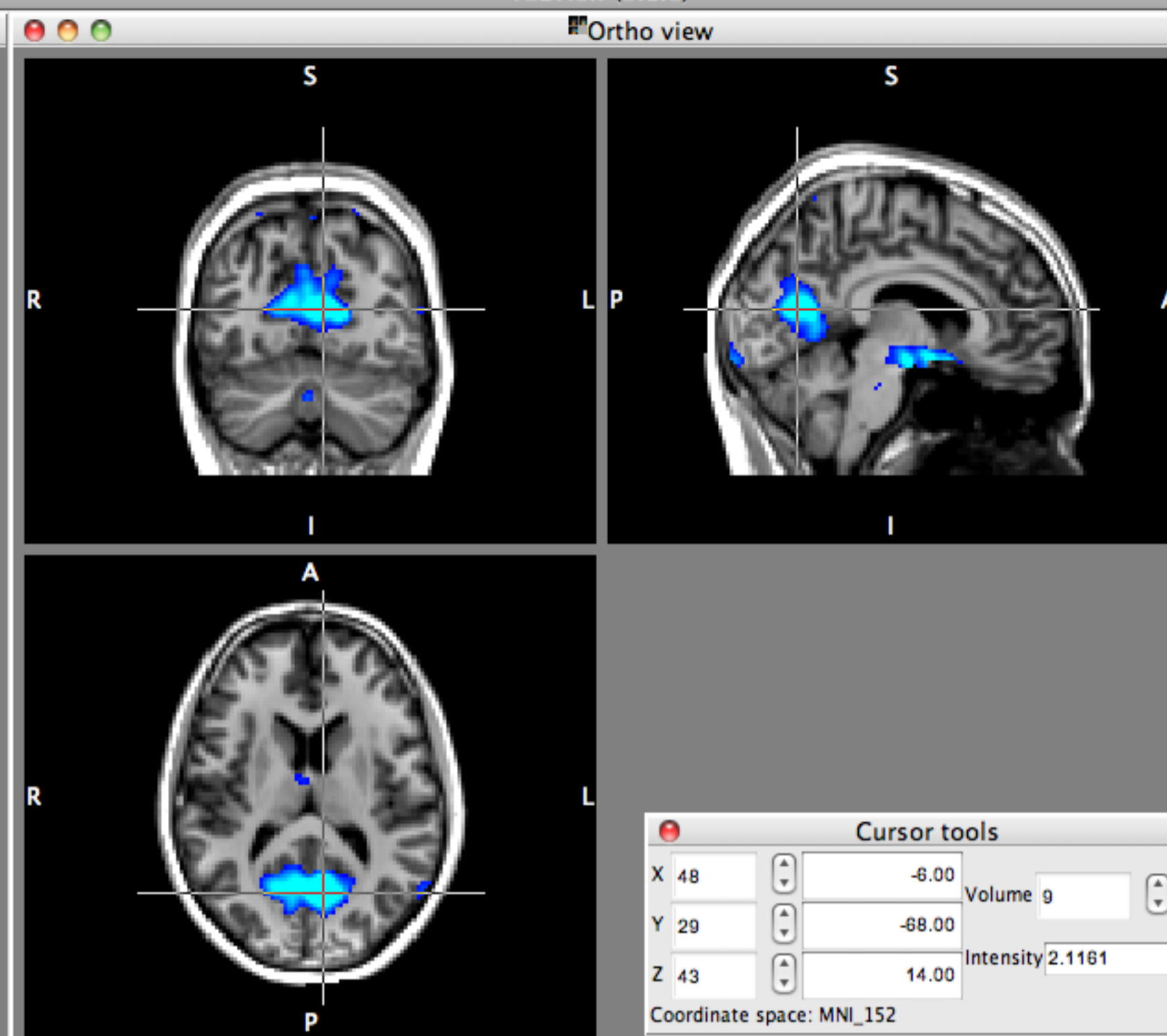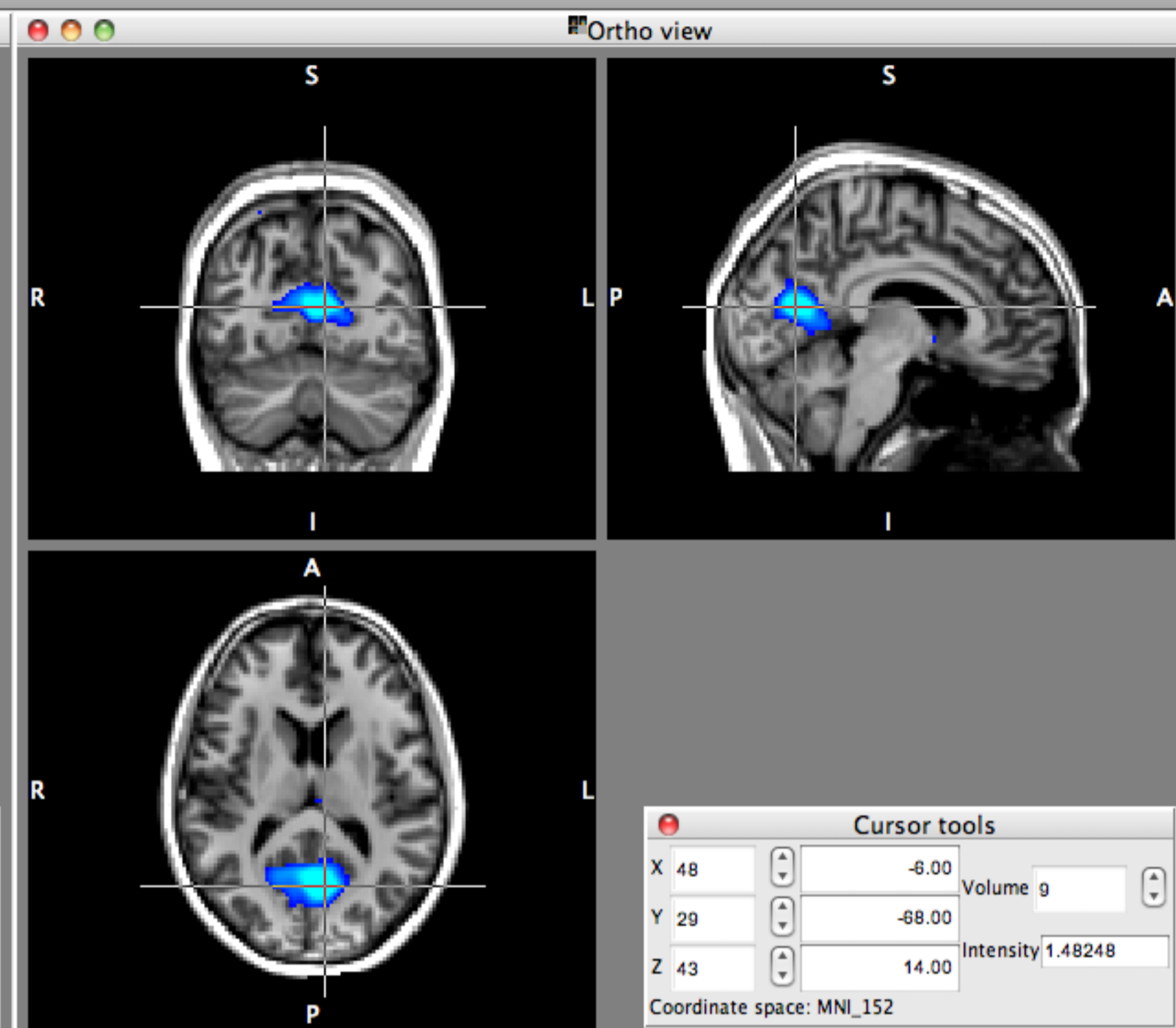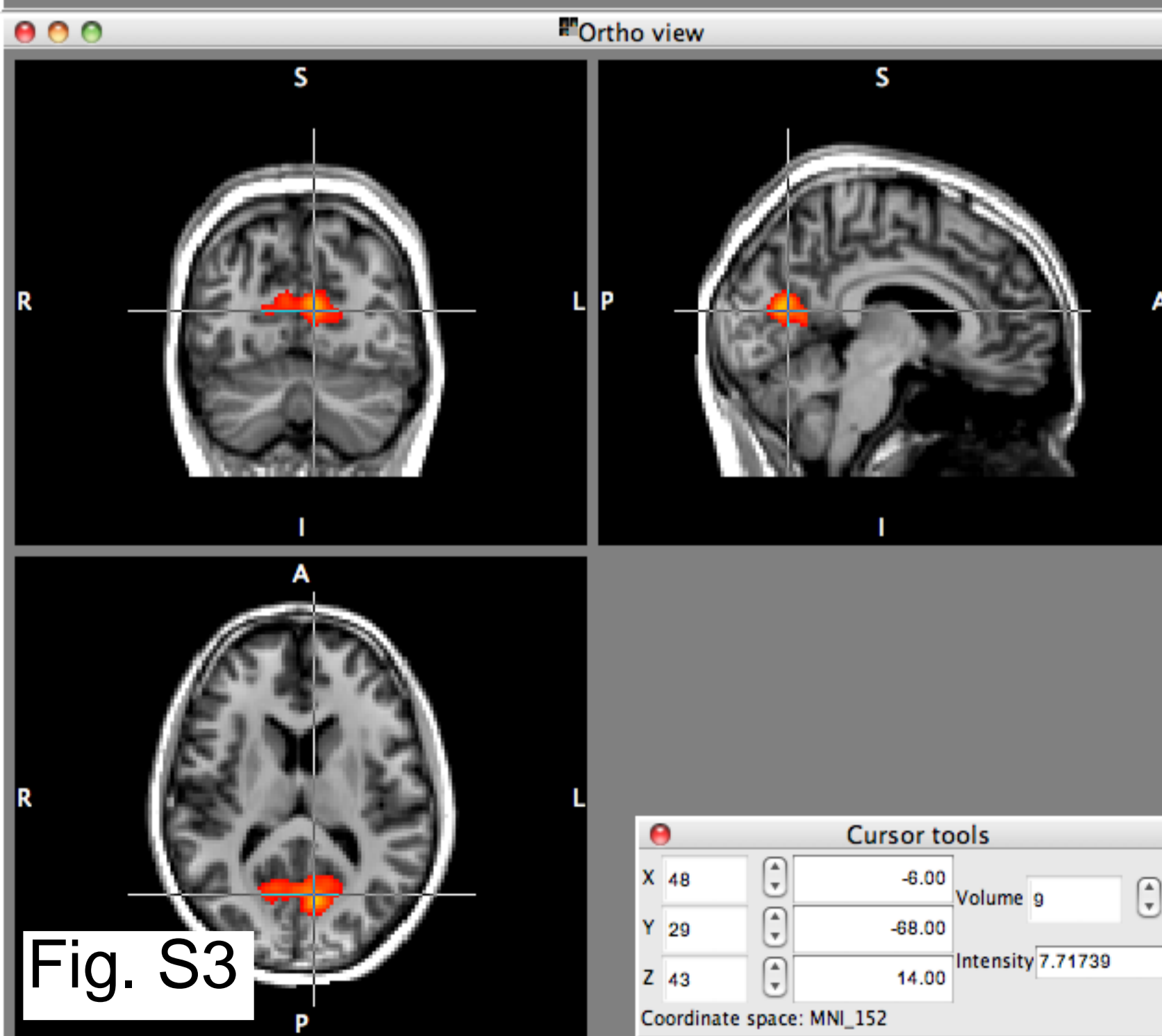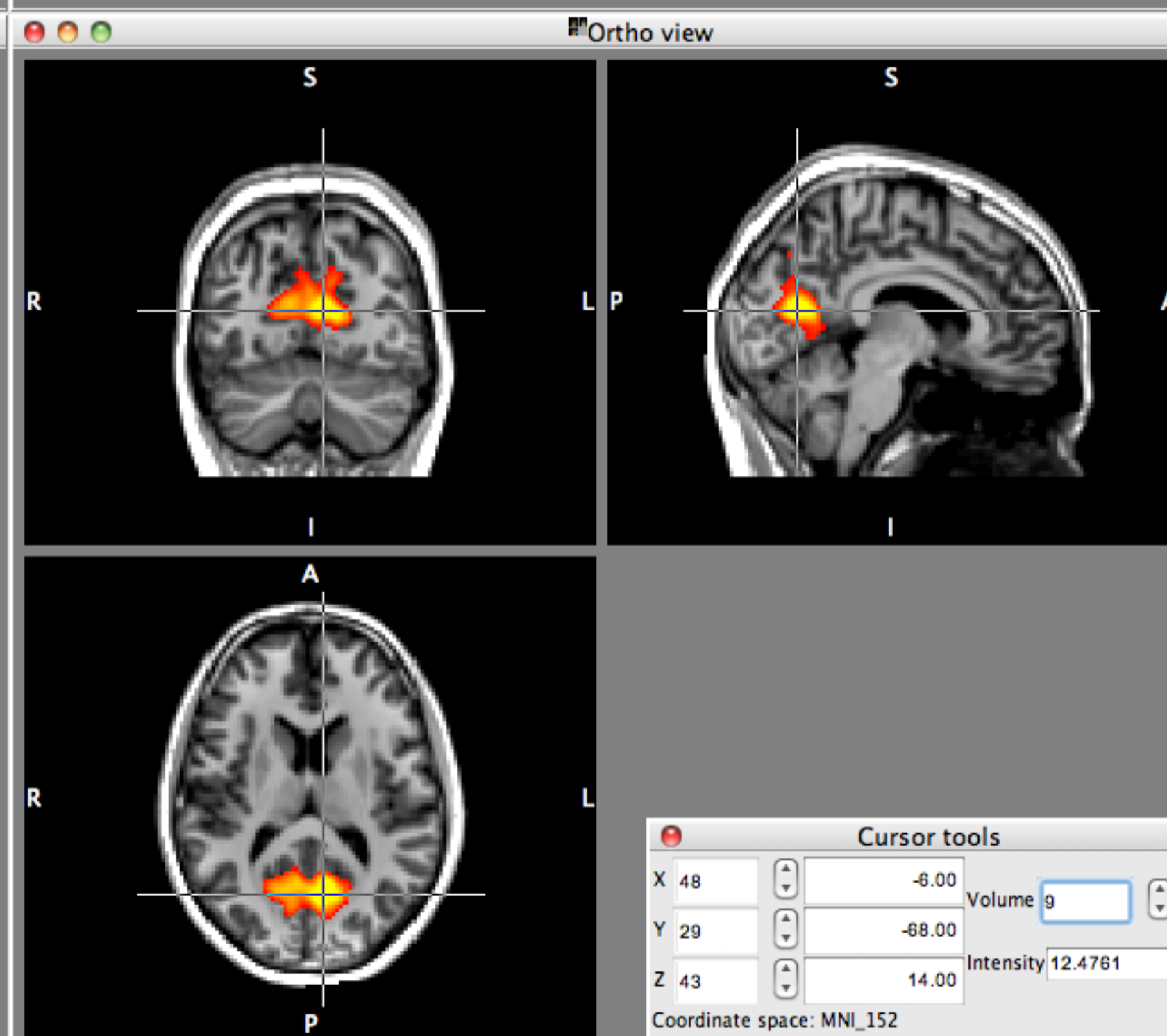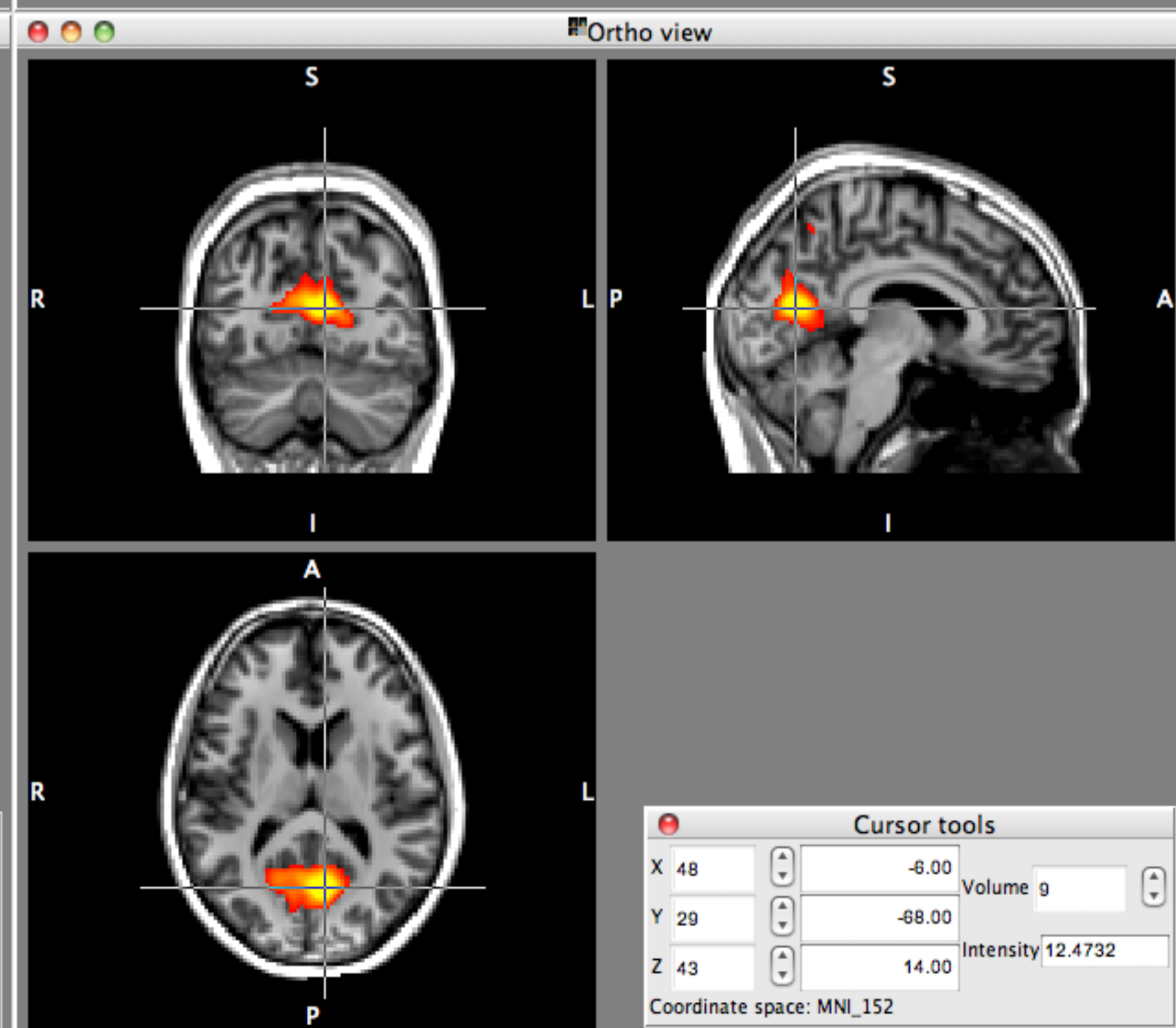

Fig. S3

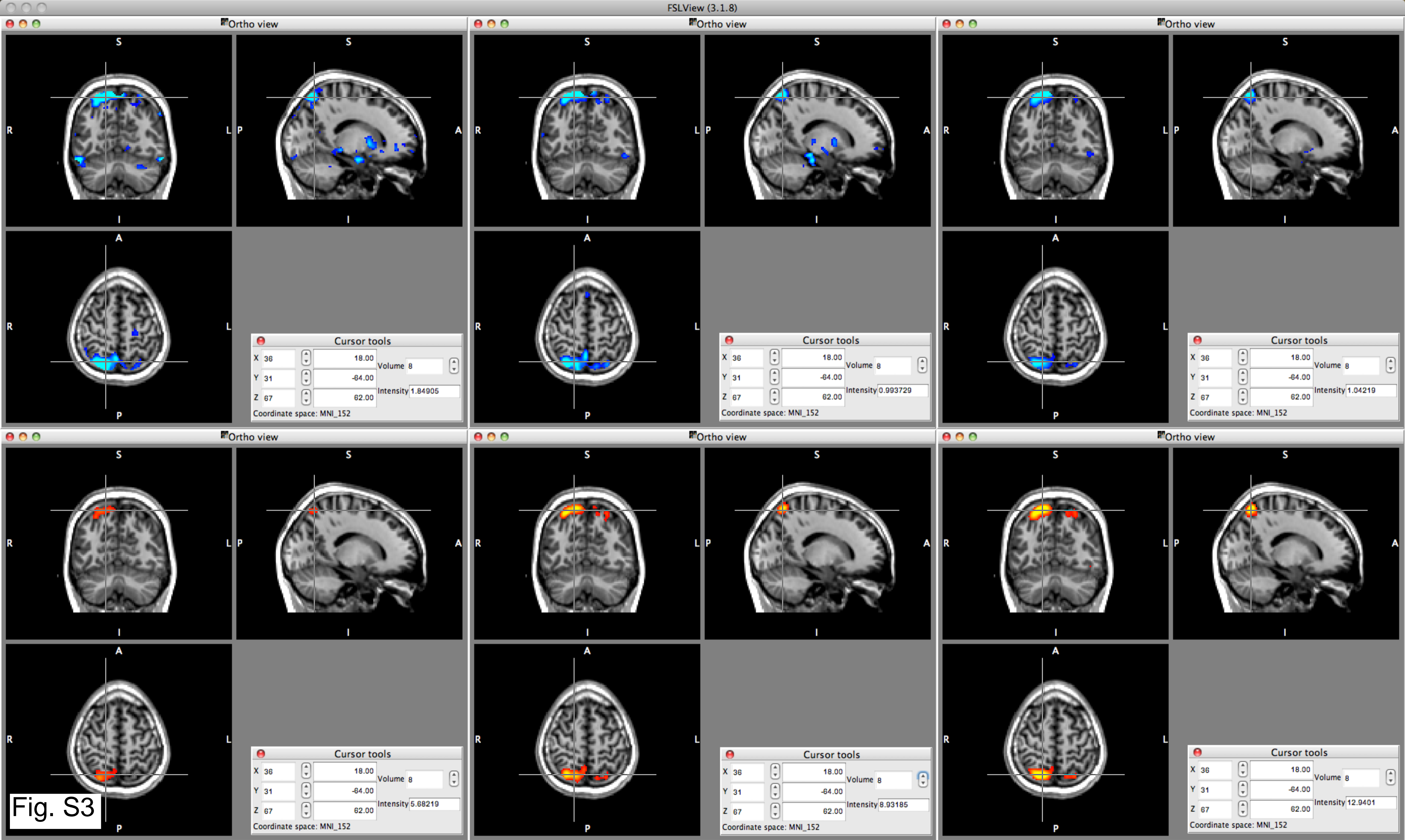

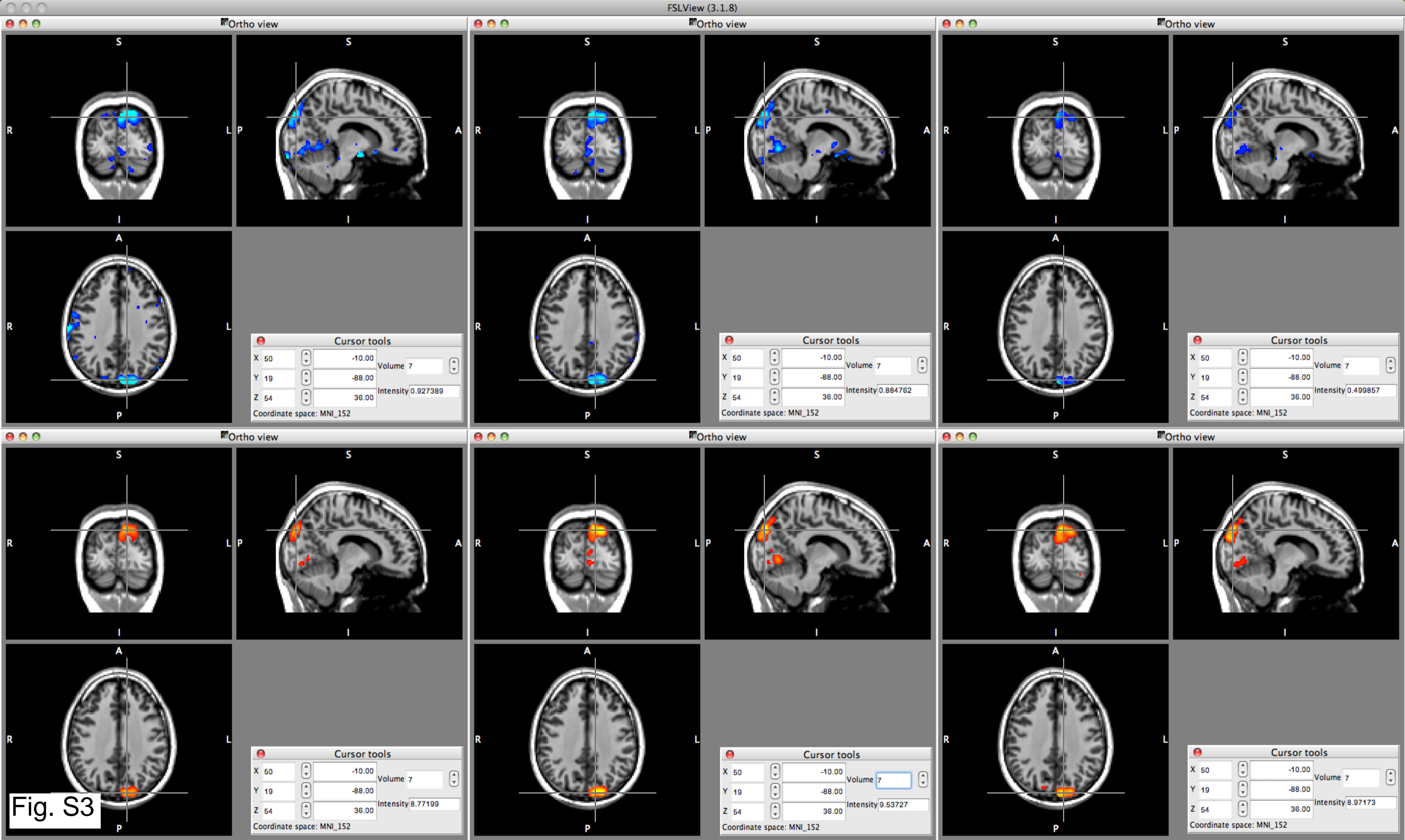

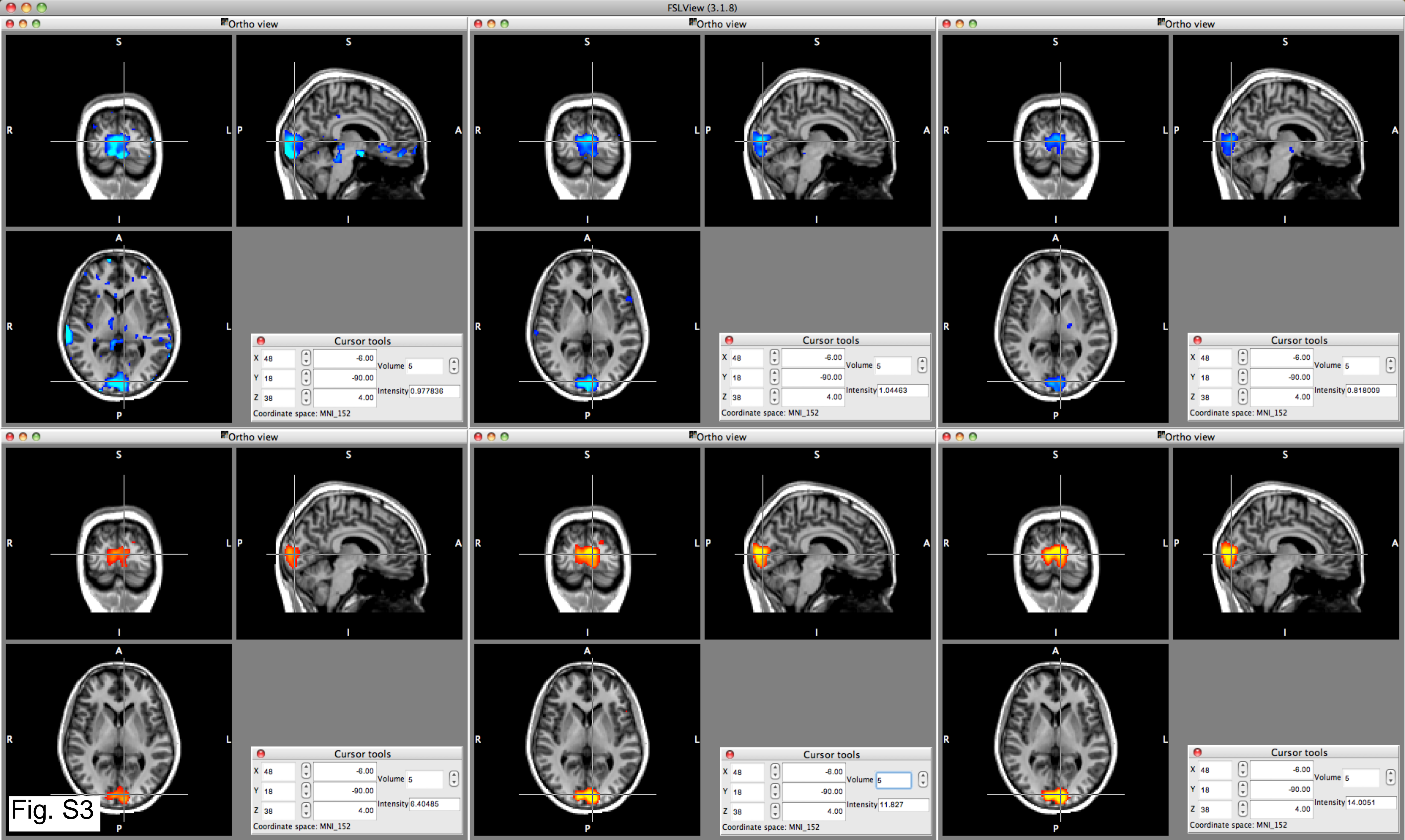

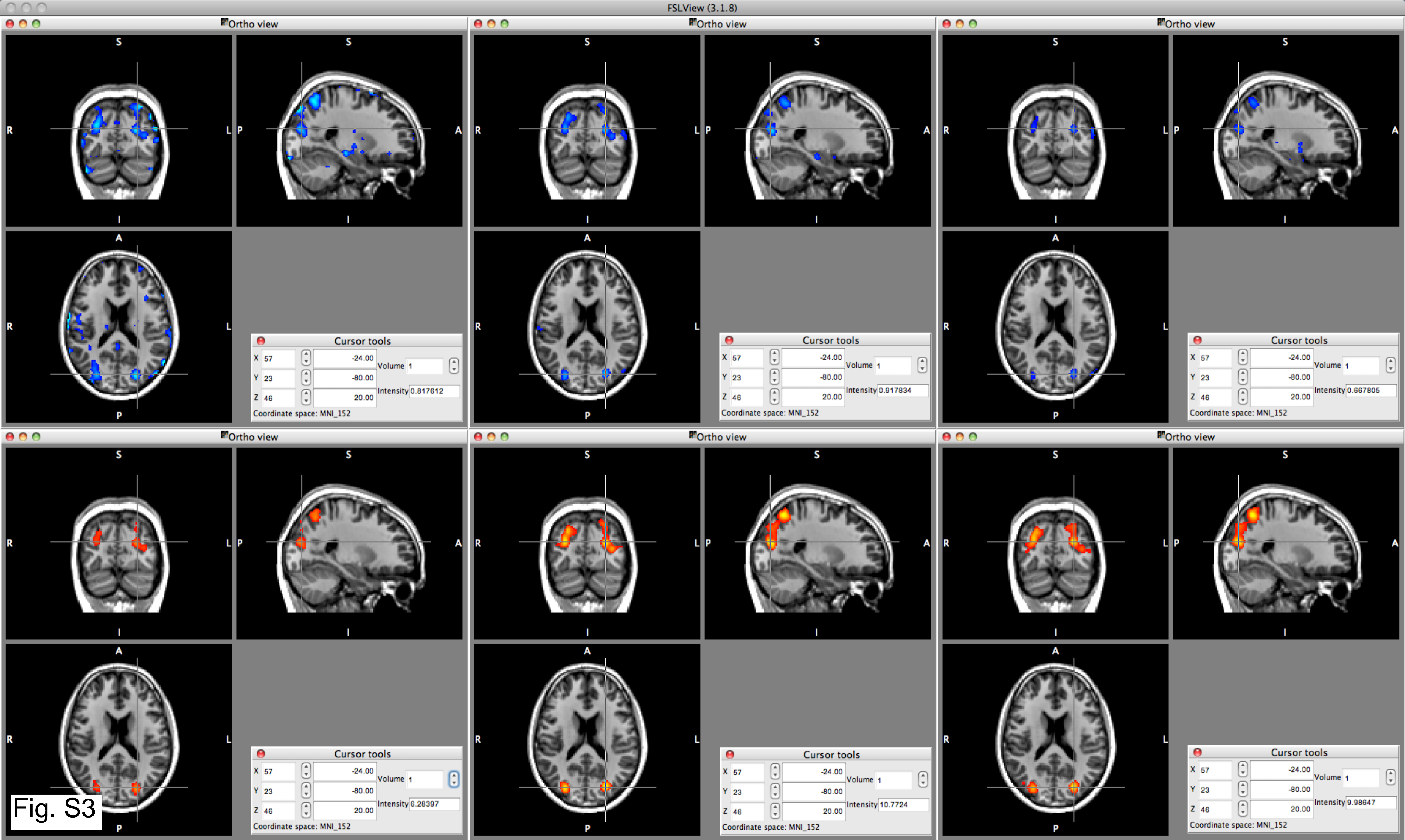

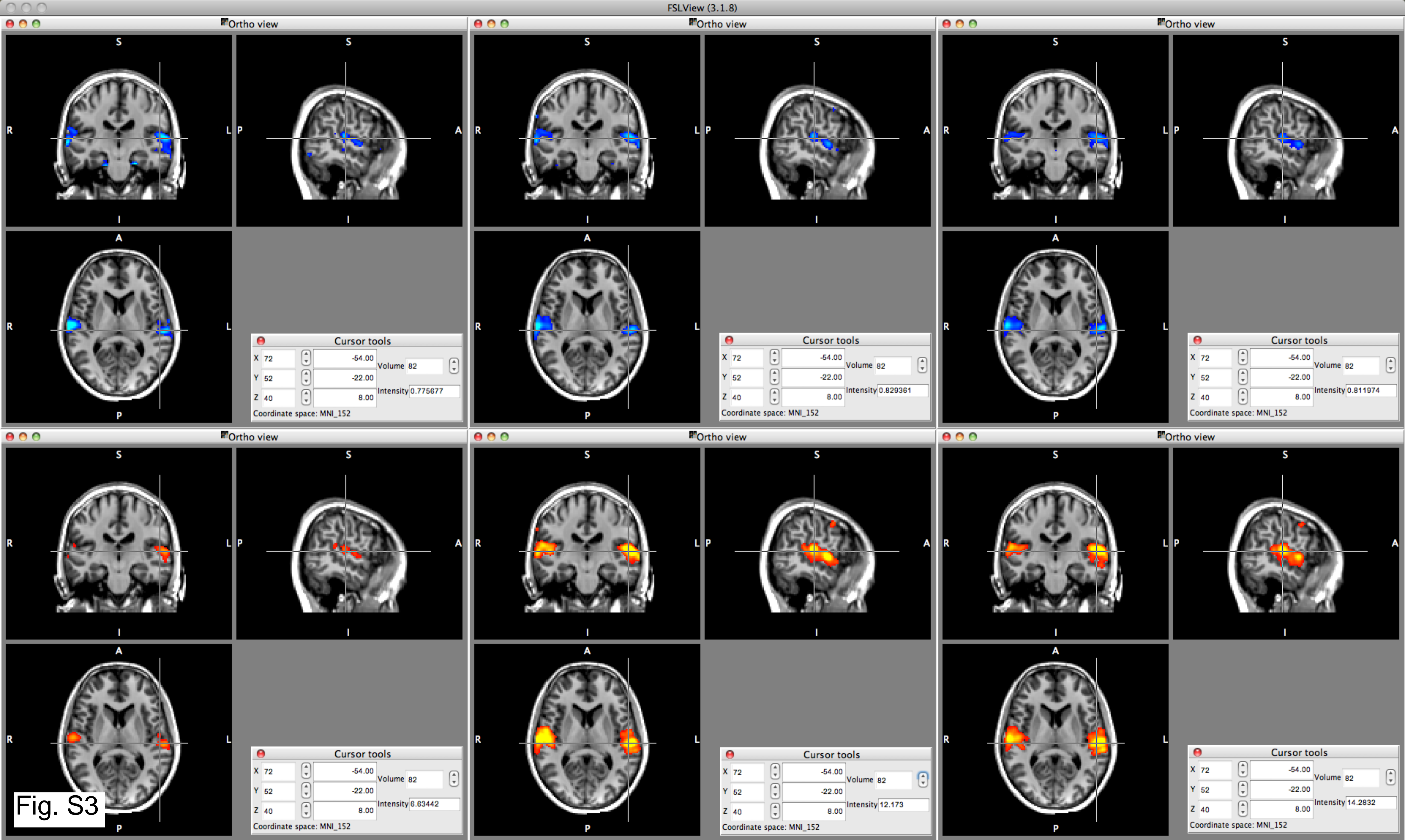

Supplement: Figure S3 — 25 example RSNs from the 100-dimensional group-ICA (followed by dual-regression) analysis of the 3 datasets from subject 2. The top row shows in blue the BOLD amplitude as a percent signal change, thresholded at 0.4. The bottom row shows in red-yellow the Z-stat (effective CNR) thresholded at 4. The three columns are, from left to right: TR = 2.5 s, 0.8 s, 0.4 s. The "Intensity" values shown refer to the value of the percent signal change or Z-stat at the position of the cross-hair. (PDF) [file pone.0015710.s003.pdf]
