# Supplementary material for: Multiplexed Echo Planar Imaging for Sub-Second Whole Brain FMRI and Fast Diffusion Imaging
Source: PLoS One. 2010 Dec 20;5(12):e15710. doi: 10.1371/journal.pone.0015710 (PMC3004955; doi:10.1371/journal.pone.0015710)
Supplement: Table S1 — The minimum repetition times (TRs) for acquiring whole brain imaging with the specified M-EPI sequence. Unless noted, the calculations are for 60 slices for the 2 mm acquisitions and 36 slices for the 3 mm. The average time per slice or this case the minimum time per slice is the TR divided by the number of slices. (PDF) [file pone.0015710.s004.pdf]

| SIR | MB | Min TR (2mm) (60sl) (msec) | Min TR (3mm) (36sl) (msec) |
|---|---|---|---|
| 1 | 1 (normal EPI) | 4300 | 2250 |
| 2 | 1 | 2770 | |
| 1 | 2 | 2150 | |
| 3 | 1 | 2180 | |
| 4 | 1 | 1890 | |
| 1 | 3 | 1440 | |
| 2 | 2 | 1390 | 675 |
| 3 | 2 | 1100 | |
| 1 | 4 | 1080 | |
| 4 | 2 | 976 (64sl) | |
| 2 | 3 | 922 | |
| 3 | 3 | 781 (63sl) | 338 |
| 2 | 4 | 749 (64sl) | |
| 4 | 3 | 630 | |
| 3 | 4 | 546 | |
| 4 | 4 | 489 (64sl) | 189 (32sl) |

Table S1
