# Supplementary material for: Multiplexed Echo Planar Imaging for Sub-Second Whole Brain FMRI and Fast Diffusion Imaging
Source: PLoS One. 2010 Dec 20;5(12):e15710. doi: 10.1371/journal.pone.0015710 (PMC3004955; doi:10.1371/journal.pone.0015710)
Supplement: Table S2 — Results of the g-factors and SNR for the different M-EPI acquisitions. The 2 mm isotropic acquisitions and SNR values for the different M-EPI were acquired fully relaxed with 90 degree flip angles. The 3 mm resting state fMRI image acquisitions were acquired using flip angles of 90° (1×1), 60° (2×2), and 50° (3×3) with TRs of 2.5 s, 0.8 s, and 0.4 s, respectively. (PDF) [file pone.0015710.s005.pdf]

2 mm isotropic	
M x N (SIR x MB)	Mean g-factors
1x1	1.00 +/- .1
2x2	1.58 +/- .34
3x2	1.61 +/- .69
4x3	3.37 +/- .89
4x4	4.16 +/- 1.25
3 mm isotropic	
M x N (SIR x MB)	Mean SNR
1x1	209
2x2	162
3x3	113

Table S2
