# Supplementary material for: Multiplexed Echo Planar Imaging for Sub-Second Whole Brain FMRI and Fast Diffusion Imaging
Source: PLoS One. 2010 Dec 20;5(12):e15710. doi: 10.1371/journal.pone.0015710 (PMC3004955; doi:10.1371/journal.pone.0015710)
Supplement: Table S3 — Results of the probabilistic fiber orientation estimation using diffusion data with different slice accelerations. 2nd to 1st is the proportion of voxels that had a 2nd fiber orientation above threshold when the first was above threshold. 3rd to 1st is the same proportion applied to 3rd fibers. Dispersion (i.e. orientation estimation uncertainty) is also reported for each fiber as well. Values are reported from white matter only as defined by segmentation of T1 weighted anatomical images. HARDI data were acquired at 3T using 2 mm isotropic resolutions with 71 diffusion directions and a b value of 2000 s/mm2. The total acquisition time was 11 min., 7.5 min., 5.5 min., and 3.5 min. for the 1×1, 2×1, 1×2, and 2×2 accelerations, respectively. (PDF) [file pone.0015710.s006.pdf]

SIR x MB (mxn)	2nd To 1st	3rd To 1st	1st Dispersion	2nd Dispersion	3rd Dispersion
1 x 1	.606	.050	.0381	.079	.112
1 x 2	.626	.046	.0321	.069	.099
2 x 1	.492	.034	.0637	.105	.138
2 x 2	.462	.021	.0647	.105	.149

Table S3
