# Supplementary material for: Multiplexed Echo Planar Imaging for Sub-Second Whole Brain FMRI and Fast Diffusion Imaging
Source: PLoS One. 2010 Dec 20;5(12):e15710. doi: 10.1371/journal.pone.0015710 (PMC3004955; doi:10.1371/journal.pone.0015710)
Supplement: Text S1 — Supplemental text. (PDF) [file pone.0015710.s007.pdf]

Supplemental Text

Optimization of SIR

SIR multiplexing causes a lengthening of the excitation and readout periods (Fig. 1), so it yields less than a 100% time reduction. SIR utilizes additional adjacent RF excitation pulses and the signal resulting from different slices are readout sequentially on each flat top of the switched polarity readout gradient. Using both MB and SIR, the scan time reduction for an EPI image slice (Tsl) is expressed as the total sequence time divided by the product of SIR and MB image encodings : Tsl = Tpre + (Npe/R) ((SIR * Tr) + Tsw) (SIR * MB) $^{-1}$ where Tpre (”waiting time”) is the initial pulse sequence time before the first signal recording and depending on different sequence designs may include time delays for increased T2* BOLD contrast or additional gradient pulses for diffusion contrast (Npe –phase encoded lines in 2D k-space, R- reduction factor of acquired signals from PPA parallel imaging, Tr – time of signal, Tsw – Gr switching time which simultaneously refocuses signals, SIR, MB - are the number of images multiplexed, respectively). The resulting time reduction in SIR diffusion imaging (1) is greatly impacted by the ratio of Tpre to the total echo train readout, which can be ~2-3 to 1 (i.e. 80 msec vs 30 msec).

The sharing of Tpre with 2 or more slices subsequently creates a large gain in sequence efficiency, defined here as net time of ADC signal encoding per total sequence time. Another gain in efficiency in SIR is by the sharing of the many gradient switchings, Tsw. MRI scanners with slower gradient switching capabilities or gradient slew rates (i.e. longer Tsw), and lower resolution imaging with shorter Tr, are more efficiently performed with higher SIR factors. The time gains for the different SIR/MB combinations for a 3T Siemens Trio system are summarized in **Table S1**.

SNR in Multiband (MB)

The combined SNR reduction of PPA and MB are: $SNR_{red} = SNR_{full}(g_{MB} g_{PE} \sqrt{R_{PE}}\ )^{-1}$, where g_{PE} and g_{MB} are the g-factors due to coil encoding and unaliasing capabilities on the in-plane axes and the slice axis, respectively. The SNR is spatially varying, since the loss due to image separation (g-factors) are calculated using the Pseudo-Replica method (2) to capture the spatial distribution, and the estimated relative SNR is obtained simultaneously. Additionally, this was also validated with image based ROI methods. For this, the standard deviation of the background was used to estimate the noise, and the ratio of the mean signal in the brain to the noise was used as a measure of relative SNR. Such a measure does not capture the spatial distribution of the noise in the brain, but is a common technique. G-factors will be both coil and static field dependent, improving with the number of channels available and the geometry of the distribution of the individual elements. Although

not trivial, due to the tight FOV employed, controlled aliasing approaches can be explored to possibly reduce g-factors (3) in multibanded sequences. **Table S2** provides $g_{MB}g_{PE}$ values for 3T with the 32 channel coil employed for these studies.

Supplementary References:

1. Reese TG, Benner T, Wang R, Feinberg DA, & Wedeen VJ (2009) Halving imaging time of whole brain diffusion spectrum imaging and diffusion tractography using simultaneous image refocusing in EPI. *J Magn Reson Imaging* 29(3):517-522.
2. Robson PM, *et al.* (2008) Comprehensive quantification of signal-to-noise ratio and g-factor for image-based and k-space-based parallel imaging reconstructions. *Magn Reson Med* 60(4):895-907.
3. Breuer FA, *et al.* (2005) Controlled aliasing in parallel imaging results in higher acceleration (CAIPIRINHA) for multi-slice imaging. *Magn Reson Med* 53(3):684-691.
